# 1980s–2010s: The world's largest mangrove ecosystem is becoming homogeneous

**DOI:** 10.1016/j.biocon.2019.05.011

**Published:** 2019-08

**Authors:** Swapan Kumar Sarker, Jason Matthiopoulos, Sonia N. Mitchell, Zahir Uddin Ahmed, Md. Bashirul Al Mamun, Richard Reeve

**Affiliations:** a Boyd Orr Centre for Population and Ecosystem Health, Institute of Biodiversity, Animal Health and Comparative Medicine, College of Medical Veterinary and Life Sciences, University of Glasgow, Glasgow G12 8QQ, United Kingdom; b Planning Wing, Bangladesh Forest Department, Ban Bhaban, Agargaon, Dhaka 1207, Bangladesh; c Sundarbans West Forest Division, Bangladesh Forest Department, Khulna 9100, Bangladesh

**Keywords:** Alpha diversity, Beta diversity, Biotic homogenization, Gamma diversity, Protected area, Species composition

## Abstract

Knowledge gaps in spatiotemporal changes in mangrove diversity and composition have obstructed mangrove conservation programs across the tropics, but particularly in the Sundarbans (10,017 km^2^), the world's largest remaining natural mangrove ecosystem. Using mangrove tree data collected from Earth's largest permanent sample plot network at four historical time points (1986, 1994, 1999 and 2014), this study establishes spatially explicit baseline biodiversity information for the Sundarbans. We determined the spatial and temporal differences in alpha, beta, and gamma diversity in three ecological zones (hypo-, meso-, and hypersaline) and also uncovered changes in the mangroves' overall geographic range and abundances therein. Spatially, the hyposaline mangrove communities were the most diverse and heterogeneous in species composition while the hypersaline communities were the least diverse and most homogeneous at all historical time points. Since 1986, we detect an increasing trend of compositional homogeneity (between-site similarity in species composition) and a significant spatial contraction of distinct and diverse areas over the entire ecosystem. Temporally, the western and southern hypersaline communities have undergone radical shifts in species composition due to population increase and range expansion of the native invasive species *Ceriops decandra* and local extinction or range contraction of specialists including the globally endangered *Heritiera fomes*. The surviving biodiversity hotspots are distributed outside the legislated protected area network. In addition to suggesting the immediate coverage of these hotspots under protected area management, our novel biodiversity insights and spatial maps can form the basis for spatial conservation planning, biodiversity monitoring and protection initiatives for the Sundarbans.

## Highlights

• We uncover spatiotemporal changes (1986–2014) in mangrove biodiversity in the Sundarbans.
• Biotic homogenization has been underway in the Sundarbans.
• Drastic loss of biomass-productive species suggests declining ecosystem carbon stock.
• Hypersaline areas experienced highest turnover in species composition.
• The Protected Area Network does not include the biodiversity hotspots, and hence requires reconfiguration.

## 1 Introduction

Historical anthropogenic pressures and rapid environmental changes have turned tropical and sub-tropical mangrove forests into one of Earth's most threatened ecosystems, causing worldwide loss of coastal livelihoods and ecosystem services (Huxham et al., 2017). Since 1950, we have lost nearly 50% of global mangrove coverage (Feller et al., 2010). The current rate of mangrove deforestation is 1–2% per year (Alongi, 2015). Such losses may become further accelerated due to predicted sea level rise (SLR) (Gilman et al., 2008; Ward et al., 2016). Despite the drastic nature of these losses, we have a restricted understanding of how mangrove diversity and composition have changed across space and through time. Such knowledge gaps have obstructed mangrove conservation programs across the tropics (Romañach et al., 2018; Sarker et al., 2016).

Different aspects of biodiversity such as alpha, beta and gamma diversity, represent different fundamental aspects of natural communities which can be of particular conservation interest (Socolar et al., 2015). For example, spatial maps of species richness (a measure of alpha diversity) can guide us in locating the biodiversity hotspots while analyses of long-term changes in species composition (beta diversity) and overall diversity (gamma) can provide insights on species invasion, extinction and biotic homogenization (Smart et al., 2006; Gaston and Fuller, 2008; Jarnevich and Reynolds, 2010). Therefore, to serve long-term conservation and protection of threatened flora, fauna and habitats, we need to look at spatial and temporal changes in all aspects of biodiversity.

However, mangrove ecologists have mostly relied on alpha diversity, in particular, the species richness index (Ellison, 2001; Record et al., 2013; Osland et al., 2017) that does not account for the abundance-related heterogeneity in vegetation structure. In fact, the ‘biodiversity anomaly’ question – explaining why mangrove tree species richness decreases along the latitudinal gradient (Ricklefs et al., 2006) – has dominated the mangrove biodiversity literature for the last two decades. While such global studies offer a broader insight into biodiversity patterns, management and conservation programs are essentially implemented based on regional or local needs.

This study focused on the mangrove tree communities of the world's largest mangrove ecosystem – the Sundarbans – which supports the livelihood of 4.5 million people in Bangladesh and India, protects them against cyclones and tidal surges, and acts as a safe haven for many globally endangered plant and animal species (Sandilyan and Kathiresan, 2012). It was designated a Ramsar site under the Ramsar Convention in 1992 and United Nations Educational, Scientific and Cultural Organization (UNESCO) declared the Bangladesh Sundarbans a World Heritage Site in 1997, because of its ‘Outstanding Universal Value’ (Gopal and Chauhan, 2006). However, this global priority ecosystem has been gradually subjected to high levels of stress by a historical and ongoing reduction in freshwater flows in the river system and salinity intrusion (Ellison et al., 2000; Islam and Bhuiyan, 2018; Wahid et al., 2007). The population sizes of many endangered tree species have substantially declined, primarily because of increasing salinity stress (Sarker et al., 2016). Further deterioration of the ecosystem through SLR is certain to influence the remaining populations of the mangroves and their spatial distributions.

Based on soil salinity, in the 1980s the Bangladesh Sundarbans was divided into three ecological zones, the hyposaline (<2 dS m^−1^), mesosaline (2–4 dS m^−1^) and hypersaline zone (>4 dS m^−1^) (Siddiqi, 2001). Management and conservation decisions are made based on the status of tree growth and forest stock in these ecological zones (Iftekhar and Saenger, 2008). But since the construction of the Farakka barrage in India in 1975, the freshwater supply into the Sundarbans has declined by 90% and the salinity level has increased by 60% (Aziz and Paul, 2015). As a result, hyposaline areas are transforming into mesosaline areas and mesosaline areas are transforming into hypersaline areas (Ghosh et al., 2016). Recently, Sarker et al., 2019, Sarker et al., 2016, using 2014 data, have reported salinity, siltation, disease outbreak and historical harvesting as the key stressors causing population decline of many specialists (including the globally endangered *Heritiera fomes*) and overall loss of mangrove biodiversity in the Sundarbans. A number of other studies (Iftekhar and Saenger, 2008; Islam et al., 2016, Islam et al., 2014) have also tried to describe the overall biodiversity of the Sundarbans. Despite these attempts, we still lack a spatially explicit understanding of how the different aspects of biodiversity (alpha, beta and gamma) and how the geographic range and abundance of mangroves species have changed in the ecological zones since 1986.

We used mangrove tree data spanning 28 years, collected in 1986, 1994, 1999, and 2014, from a network of 110 permanent sample plots (PSPs) covering the entire Sundarbans. Our main goal was to understand the spatial structure of the biodiversity components – within-plot (alpha), between-plot (beta), and total (gamma) diversity – at these four historical time points and to uncover the temporal dynamics in species composition both within the ecological zones and across the whole Sundarbans ecosystem. More precisely, we asked the following questions: Which ecological (i.e. salinity) zone supports the most/least diverse mangrove communities? Is the most diverse ecological zone also the most heterogeneous (i.e. variable between plots) in species composition? How has compositional heterogeneity in the broader ecological zones developed over the last 28 years? How has the geographic range and abundance of mangroves changed since 1986? We also developed spatial alpha, beta and gamma diversity maps to answer the following questions: Where are the historical and contemporary biodiversity hotspots located? Which habitats have changed most in species composition over time? Finally, we demonstrated the potential applications of these new maps and insights in the ongoing and future mangrove enhancement, restoration and protection initiatives in the Sundarbans.

## 2 Materials and methods

### 2.1 Study system

**Fig. 1:**
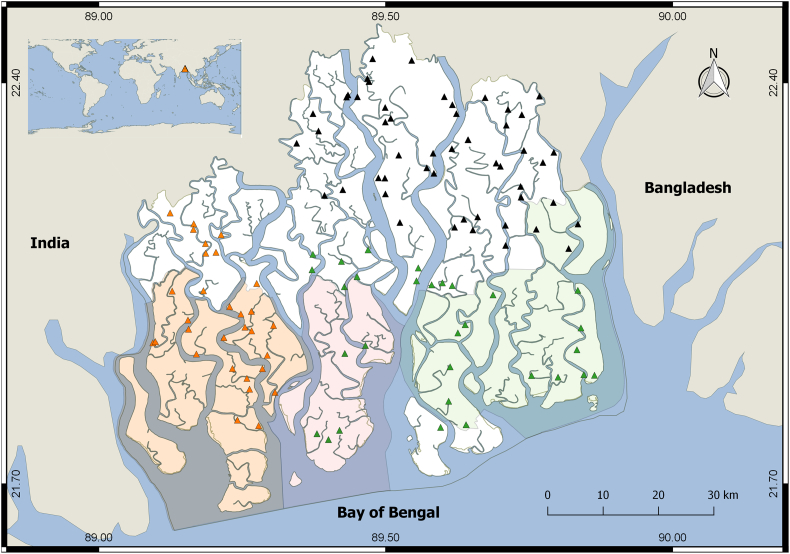
Permanent sample plots (PSPs) in the Sundarbans, Bangladesh. Black, green, and orange triangles represent the PSPs located within the hypo-, meso- and hypersaline ecological zones, respectively. Blue areas represent water bodies. Areas shaded by orange, pink and green colored polygons represent the three protected areas: Sundarbans West, South and East Wildlife Sanctuaries, respectively. (For interpretation of the references to color in this figure legend, the reader is referred to the web version of this article.)

The Bangladesh Sundarbans (21°30′-22°30′N, 89° 00′-89°55′E, Fig. 1) is part of Ganges-Brahmaputra delta, the world's largest estuary. The soil type is silty–clay-loam. The soil of the hyposaline areas (eastern and north-eastern regions) is relatively fertile than the meso- (central and southern regions) and hypersaline (western and south-western regions) zones (Siddiqi, 2001). There are two high and two low tides, and the tidal amplitudes are higher (~5 m) in the hypersaline habitats than the hypo- and mesosaline habitats (3–4 m) (Chowdhury et al., 2016). Regional hydrology depends on the freshwater flows from the Ganges and the saltwater influx from the Bay of Bengal. The climate is humid tropical. The average annual precipitation is 1700 mm, and the average temperature in pre-monsoon, monsoon, post-monsoon and dry winter is 29, 30, 26 and 20 °C, respectively (Chowdhury et al., 2016).

### 2.2 Tree data

Tree data were collected from the PSP network (Fig. 1) in the Sundarbans during four complete forest censuses: 1986, 1994, 1999 and 2014. Every tree with d.b.h (diameter at breast height – 1.3 m from the ground) ≥4.6 cm was tagged with a unique tree number and identified. The network consists of 110 equal-sized plots (0.2 ha, 100 × 20 m) and represents the forest types in the ecological zones (see Iftekhar and Saenger, 2008). The hyposaline zone comprises 50 PSPs representing the *Heritiera fomes*, *H. fomes – Excoecaria agallocha*, and *E. agallocha – H. fomes* forest types. The mesosaline zone comprises 30 PSPs representing the *H. fomes – E. agallocha*, and *E. agallocha – H. fomes* forest types. The hypersaline zone comprises 30 PSPs representing the *E. agallocha – Ceriops decandra* and the *C. decandra – E. agallocha* forest types.

### 2.3 Diversity partitioning

We used the unified framework developed by Reeve et al. (2016) for partitioning diversity because it allows us to investigate the hierarchical structure of diversity in a highly complex ecosystem such as the Sundarbans by assessing diversity at three different spatial scales, at the scale of the ecosystem as a whole, of the three ecological zones, and of the 110 individual PSPs, and also, at four different time points (1986, 1994, 1999, and 2014). This framework, based on Rényi's notion of generalised relative entropy (Rényi, 1961), and extending Hill (1973), Jost, 2006, Jost, 2007 and Leinster and Cobbold's (2012) notions of ecosystem diversity, partitions ecosystem diversity in a way that helps us to understand the true subcommunity alpha, beta and gamma diversity structure and dynamics. These individual subcommunity measures provide biological insights into the importance of individual communities across an ecosystem, allowing us to directly compare subcommunities across the Sundarbans or an individual subcommunity over time.

**Fig. 2:**
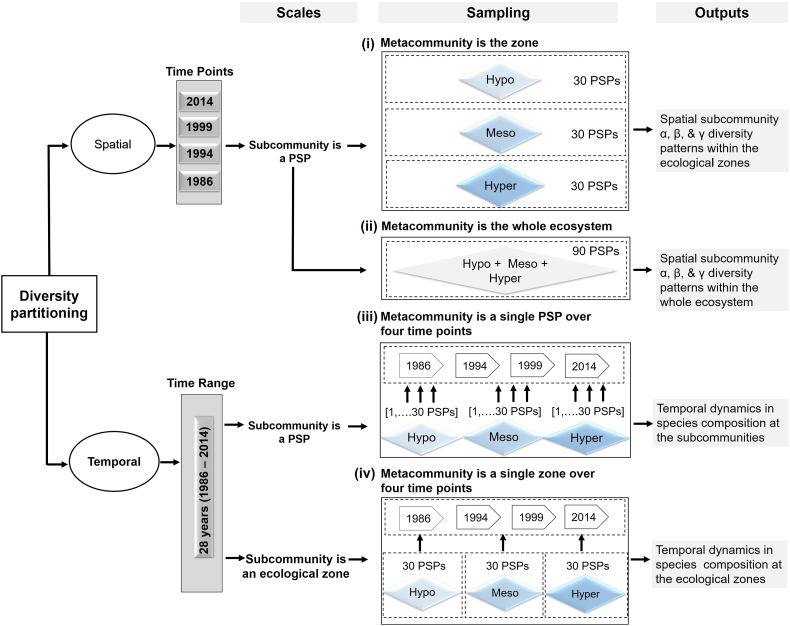
Biodiversity partitioning scheme used in this study to explain spatial subcommunity (SC) alpha, beta, and gamma diversity structures across (i) the ecological zones (i.e. hypo-, meso-, and hypersaline zones) and (ii) the whole ecosystem (Sundarbans) in four historical time points (in 1986, 1994, 1999 and 2014), and to investigate temporal dynamics in species composition across (iii) the individual subcommunities as well as (iv) the individual ecological zones over the last 28 years.

All of the analyses are based on the comparison between the diversity of a larger area (called a *metacommunity*) with the diversity of its subcomponents (called *subcommunities*). The definition of the *metacommunity* (hereafter, MC) and its partitioning into *subcommunities* (hereafter, SCs) is done in a way suitable to the question being asked. For example, in spatial analysis, we look at a single timepoint, and an MC will be either the ecosystem or a single ecological zone, with the SC being a single PSP at that timepoint. In a temporal analysis, on the other hand, a single location – either a PSP or a whole zone forms a SC, one for each timepoint, with the MC being formed of that PSP or zone at all four time points combined. For the spatial analyses, we used two MC levels: (1) ecological zone and (2) the whole Sundarbans ecosystem — to investigate how the diversity components (i.e. alpha, beta and gamma) in each SC varies both within its ecological zone and in relation to the whole ecosystem. To avoid bias from the uneven distribution of PSPs among the ecological zones (50 in hyposaline, 30 in mesosaline, and 30 in the hypersaline zone), we repeatedly subsampled 30 PSPs from the hyposaline zone at random in the analyses (100 iterations). Thus, in analyses, each ecological zone comprised 30 PSPs i.e. SCs, and the whole Sundarbans ecosystem comprised 90 PSPs, 30 from each of the three ecological zones. Fig. 2 presents how we adapted Reeve et al.'s (2016) framework to investigate spatial and temporal diversity patterns in the Sundarbans based on our long-term (28-year) dataset.

This diversity framework, which extends and enhances existing approaches, allowed us to use identical diversity and partitioning analyses to address all of our spatial and temporal questions to ensure consistency across the whole study. In particular, we were able to make spatial and temporal comparisons of the PSPs and zones to identify which were the most diverse individually (alpha diversity), which were the most representative of the MC in terms of having similar species composition – or, conversely, which were the most distinct individually and heterogeneous as a whole (a kind of beta diversity), and which contribute the most to overall MC diversity (SC gamma diversity).

We used the normalized alpha diversity (denoted $\bar{\alpha }$) which, at the subcommunity level, represents the diversity of a single SC in isolation – a single PSP (i, ii, iii) or a single ecological zone (iv) at a single timepoint – to identify the zones with the richest local biodiversity, averaging over the PSPs as necessary to determine the most diverse zones.

The normalized beta diversity measure, $\bar{\rho }$, measures representativeness and assesses how well a SC represents the species composition of its whole MC, where a MC can be composed of a single PSP (or zone) over time (for temporal representativeness) or multiple PSPs at a single timepoint over space (for spatial representativeness). For a SC, it takes its lowest value when every tree is completely dissimilar to every other tree in the rest of the MC, at which point its value is the proportion of the total number of trees in that SC, reflecting the fact that the SC represents only itself. It is maximised (with value 1) only when the species distribution of the SC is identical to that of the MC, since it represents the whole MC perfectly (though in the limit at *q* = 0, we ignore abundance and the SC only has to contain the same species). For spatial analyses (i, ii), low representativeness therefore reflects high spatial heterogeneity in species composition within the MC, and high representativeness reflects spatial homogeneity; for temporal analyses (iii, iv) high and low turnover, respectively.

For spatial analyses at the MC level, the gamma diversity, γ, is the conventional gamma diversity (Hill, 1973; Jost, 2006; Leinster and Cobbold, 2012), reflecting the total species diversity in a single ecological zone (i) or the whole unpartitioned ecosystem (ii), while for temporal analyses, it reflects the total diversity found over all of the timepoints in each PSP (iii) or zone (iv). The spatial SC (PSP) gamma diversity (i, ii) measures each PSP's average contribution to (or influence on) the MC diversity per tree, combining the alpha diversity of a SC with its beta diversity to form an assessment of the overall contribution of the SC to the MC (Reeve et al., 2016); the temporal SC gamma diversity measures the PSP (iii) and zonal (iv) contribution to overall MC diversity per tree at different timepoints.

The values of all these diversity measures are moderated by a viewpoint parameter (as Hill numbers, Hill, 1973), *q*, which takes a value between 0 and ∞. This parameter determines how conservative our assessments are. Larger values of *q* give increasingly conservative appraisals of diversity (see Reeve et al., 2016 for details). This is achieved by reducing the importance given to outliers, such as rare species, in the case of $\bar{\alpha }\;\text{and}\;\gamma$, or focusing on the unrepresentative species, in a SC when measuring $\bar{\rho }$, to calculate a conservatively low estimate of how representative the SC is. When *q* is zero, $\bar{\alpha }$ and *γ*, measure species richness. This measure is *anti-conservative*, attributing the same importance to rare species as to common ones by counting only their presence or absence. At *q* = 1, we have a measure related to Shannon entropy (Shannon and Weaver, 1949), a measure of information gained by the next encounter. At *q* = 2, our measure is related to Simpson's concentration index. Both of these measures put more weight on the more dominant species than species richness. Therefore, for all of our analyses, we report the results using the above three values (0, 1, and 2) of *q*, writing them as α¯0,ρ¯1,γ2, etc.

### 2.4 Spatial and temporal diversity analyses

The four spatial snapshots (species counts over the plot matrix obtained in 1986, 1994, 1999 and 2014) were analysed individually to determine spatial SC level $\bar{\alpha }$, $\bar{\rho }$ and γ diversity relative to our two MC levels (Fig. 2): the ecological zone level (i) and the whole-ecosystem level (ii). We then averaged the $\bar{\alpha }$, $\bar{\rho }$ and γ diversity values of the SCs and calculated the 95% confidence intervals, using the means from the original SC calculations (arithmetic, harmonic and geometric for *q* = 0, 1 and 2, respectively (Reeve et al., 2016)). We also conducted one-way repeated measures analyses of variance (ANOVA) to determine to what extent subcommunity alpha, beta and gamma diversity (for *q* = 0, 1 and 2, and for our two MC levels) varied in the ecological zones of the Sundarbans over the last 28 years. We then performed post-hoc tests using multiple pairwise comparisons between the subcommunity alpha, beta and gamma diversity of the ecological zones to see how significantly all the diversity components differed within and between the hypo-, meso- and hypersaline zones over the four historical time points: 1986, 1994, 1999 and 2014. Repeated measures ANOVA and all post-hoc tests were conducted using the ‘psycho’ package version 0.4.0 (Makowski, 2018) in R statistical software, version 5.3.1 (R Core Team, 2018).

To understand long-term dynamics in species composition across the SCs, we estimated their temporal representativeness ($\bar{\rho }$). Following Reeve et al. (2016), here the composition of each PSP (iii) or zone (iv) summed over the four census times (1986–2014) formed the MC and each PSP/zone composition in each census time was the SC. We calculated the temporal MC $\bar{\rho }$ following the same method as for the spatial analysis. Temporal $\bar{\rho }$ values of the PSPs of each of the ecological zones were then averaged (and confidence intervals calculated) to understand which zones contained PSPs that changed the most in terms of their composition (seen as low representativeness). We also pooled the species data of the SCs (30 PSPs) of each zone and estimated temporal $\bar{\rho }$ for each of the hypo-, meso-, and hypersaline zones as a whole to understand how each ecological zone changed in species composition over the last 28 years. All the diversity analyses were performed using the ‘rdiversity’ package version 1.0 (Mitchell and Reeve, 2017) in R.

### 2.5 Biodiversity mapping

Here we considered the MC comprising all 110 PSPs surveyed at the four time points to ensure maximum area coverage and estimated the SC alpha, beta and gamma diversity. Using ordinary kriging (OK), we then interpolated these SC level values to develop spatial maps of each of the diversity facets for the four census points. The size of each grid cell of the interpolated surfaces was 625m^2^. We fitted Spherical, Exponential and Gaussian semivariogram models to each biodiversity measure and selected the model with least sum of squared errors. The Spherical model offered the best fit for all the diversity measures. Semivariograms are presented in Figs. A1, A2 and A3. To measure the uncertainty around the kriged predictions of the diversity indices under all historical time points (1986, 1994, 1999 and 2014), we used the normalized root mean square error (NRMSE) statistic derived from a leave-one-out cross-validation procedure. For normalization, the root mean square error statistic was divided by the range of the actual diversity values. To see to what spatial extent the values of the diversity indices have changed between 1986 and 2014, we calculated the number of grid cells where the values have changed in the appropriate direction and divided it by the total number of the grid cells. OK was performed using the ‘gstat’ package version 1.1-5 (Pebesma, 2004) and the spatial maps were constructed using the ‘raster’ package version 2.5-8 (Hijmans, 2017) in R. We followed a similar mapping procedure to build surfaces for temporal beta diversity ($\bar{\rho }$). This approach of mapping temporal $\bar{\rho }$ in a spatial context helps to identify areas with high temporal dynamics in species composition.

An extensive protected area network (PAN), covering three wildlife sanctuaries are operational in the Bangladesh Sundarbans. We superimposed the PAN (Fig. 1) on our spatial mangrove diversity maps to examine its ability to support the historical and current biodiversity hotspots in the Sundarbans.

### 2.6 Mangrove abundance change and range analyses

In Sundarbans, many specialists and rare endemic tree species are facing extinction (Sarker et al., 2016). Information on historical and current abundances of these critical species is essential for their protection and conservation. Hence, to derive their zone-wise historical and current abundances, we summed the PSP-level counts of each species of each ecological zone for the first (1986) and the last census (2014). Then, to calculate the percentage contribution of each species to the total composition of each ecological zone at both times, we divided the sum of the PSP-level counts of each species by the sum of the PSP-level counts of all species. Finally, to derive the zone-wise percentage composition change (% CC) for each species from 1986 to 2014, we deducted the percentage contribution of each species in each ecological zone in 1986 from that of in 2014 and the resulted value was then standardized by the sum of the percentage contributions at both times. This % CC calculation ranges from −100% (extinction) to +100% (introduction) while controlling for variation in overall mangrove abundance between dates.

Following Gillette et al. (2012), we determined how the mangrove species' range expanded or contracted in the hypo-, meso- and hypersaline zones over the last 28 years. Historical and concurrent environmental changes in the Sundarbans may promote the geographic expansion of some native invasive or generalists, and geographic contraction of habitat specialists (Biswas et al., 2018). This disproportionate expansion of generalist or invasive species relative to specialists is reported to be the key mechanism behind biotic homogenization (McKinney and Lockwood, 1999). To test this, we deducted the number of PSPs in each ecological zone at which a mangrove species occurred in 1986 from the number of PSPs at which it occurred in 2014. The resulted value was then standardized by the number of total PSPs at which the species was present at both times. This value lies between −1 to 1 (positive values indicating range expansion). New species (i.e. introduction) in the ecological zone get the highest value (=1), and the species that disappeared from the zone (i.e. local extinction) get the lowest value (=−1). A species gets 0 value if it occurs exactly in the same PSPs both in 1986 and 2014, meaning that the species range remains stable.

## 3 Results

### 3.1 Spatial and temporal dynamics

**Fig. 3:**
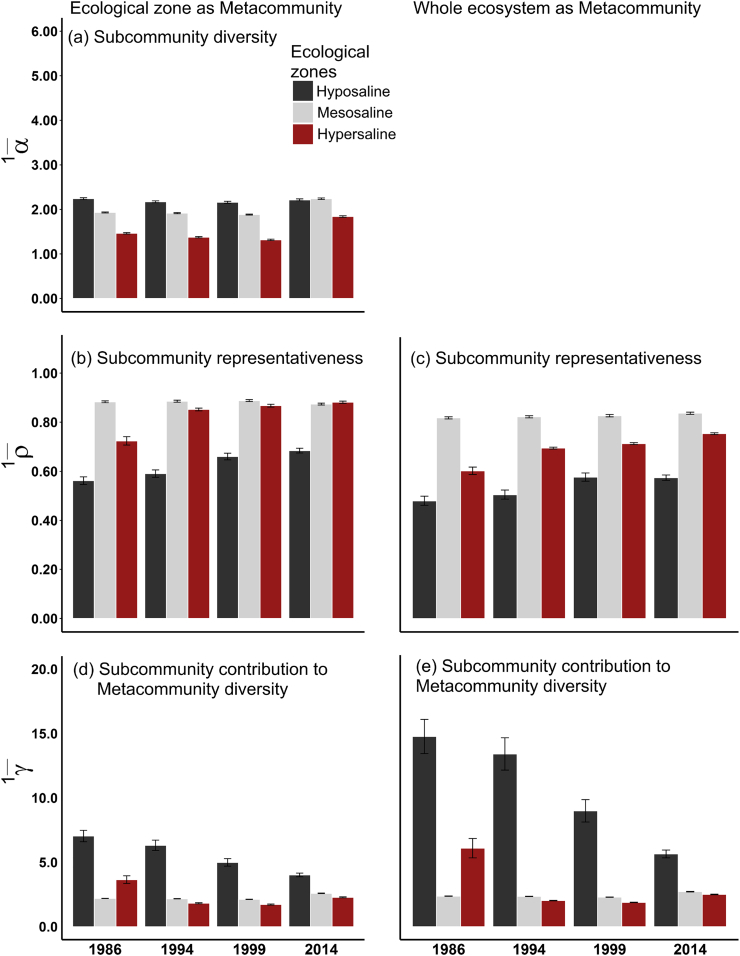
Bar charts show the spatial (a) alpha (subcommunity diversity), (b & c) beta (subcommunity representativeness), and (d & e) gamma (subcommunity contribution to metacommunity diversity) diversities at *q* = 1 level for two metacommunity levels – ecological zone and the whole ecosystem – for the four censuses: 1986, 1994, 1999 and 2014. Each permanent sample plot (PSP) is the subcommunity. Each zone as a metacommunity comprises 30 PSPs. The whole ecosystem as a metacommunity comprises 90 PSPs (30 PSPs from each of the three ecological zones). Each bar represents the mean diversity value of the PSPs in each zone or the whole ecosystem and the 95% confidence intervals.

Subcommunity (SC) alpha, beta and gamma diversity (for *q* = 0, 1 and 2) varied significantly in all the ecological zones of the Sundarbans over the last 28 years (*P* < 0.001, Table A1). Spatially, the SCs of the hyposaline zone were the most diverse (α¯1, Fig. 3a) and simultaneously the most heterogeneous in species composition (lowest representativeness, ρ¯1, Fig. 3b, c) in all historical time points (except α¯1 in 2014, *P* = 0.94), leading to the hyposaline SCs being the largest contributors to the overall diversity (per tree, ^1^*γ*, Fig. 3d, e) of the ecosystem over the last 28 years (see Table A2 for details on how all the diversity components differed within and between the ecological zones in 1986, 1994, 1999 and 2014). The hypersaline SCs were the least diverse in all historical time points although alpha diversity increased in this zone (also in the mesosaline zone) in the last 15 years (as a result of the invasion of the disturbance specialist *C. decandra*).

The SCs of the mesosaline zone were spatially the most homogeneous in species composition and stable over time (i.e. high representativeness). In contrast, since 1986, the SCs of both the hypo- and hyper-saline zones showed significant trends of increasing representativeness, $\bar{\rho }$, (i.e. decreasing compositional heterogeneity) and decreasing contribution, γ, to the overall diversity of the ecosystem (Table A2), indicating biotic homogenization and increasing dominance of generalists. Similar patterns were observed when rare species were given the same importance as the dominant species (*q* = 0, Table A2 & Fig. A4) and when relative abundances were considered (*q* = 2, Fig. A5).

The SCs of the hypersaline zone and the zone itself (sample plots pooled) had the highest temporal beta diversity (lowest representativeness, $\bar{\rho }$, Fig. A6) for all values of *q* over the last 28 years (except the zone at *q* = 0), due to drastic changes in relative abundances of the species in the hypersaline communities and especially the increasing dominance of a few generalists (e.g., *Excoecaria agallocha*) and invasion of the disturbance specialist *Ceriops decandra*.

### 3.2 Mangrove tree diversity maps

**Fig. 4:**
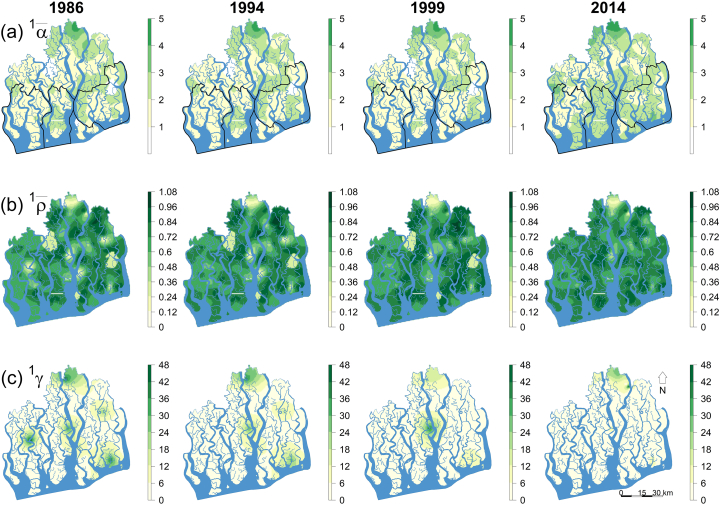
Spatial distributions of subcommunity alpha, beta and gamma diversities (for *q* = 1) over the entire Sundarbans generated through ordinary kriging. Higher values of $\bar{\alpha }$ and γ indicate greater species diversity and community contribution to the overall diversity of the ecosystem. Lower values of $\bar{\rho }$ indicate greater heterogeneity in species composition (i.e. community distinctness from the metacommunity) and higher values of $\bar{\rho }$ represent greater representativeness (i.e. homogeneity) in species composition. The black contours represent the three protected areas.

**Fig. 5:**
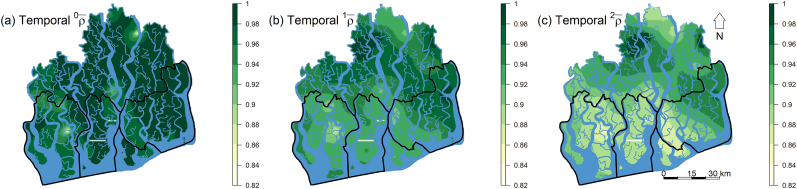
Spatial maps showing the distributions of temporal change in subcommunity representativeness (i.e. $\bar{\rho }$ at q = 0, 1, and 2) during 1986–2014 generated through ordinary kriging. Low representativeness indicates a high temporal turnover (or heterogeneity) in species composition while high representativeness suggests stable species composition over time. The black contours represent the three protected areas.

**Fig. 6:**
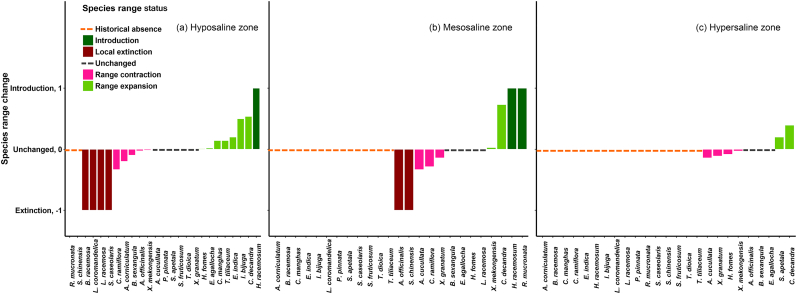
Mangrove species range change in Sundarbans' hypo-, meso- and hypersaline zones during 1986–2014. The values in the y axis lie between −1 to 1 and are derived from deducting the number of PSPs in each ecological zone at which a mangrove species occurred in 1986 from the number of PSPs at which it occurred in 2014 and then standardizing the resulted values by the number of total PSPs at which the species was present at both times. Positive values indicate range expansion and negative values indicate range contraction. New species (i.e. introduction) in the ecological zone get the highest value (=1), and the species that disappeared from the zone (i.e. local extinction) get the lowest value (=−1). A species gets 0 value if it occurs exactly in the same PSPs both in 1986 and 2014, meaning that the species range remains unchanged.

Both historically and currently, the hyposaline zone support the most biodiverse (α¯1, Fig. 4a) SCs. While alpha diversity (α¯1) has increased in 82% of the areas (grid cells, Table A1) of the Sundarbans (due to range expansion and abundance increase of the generalists *Excoecaria agallocha* and *Ceriops decandra*, see Table 1 & Fig. 6) over the last 28 years, spatial extent of the unrepresentative (i.e. distinct) SCs (ρ¯1, Fig. 4b) and also those SCs contributing most to the overall diversity of the ecosystem (^1^*γ*, Fig. 4c) have declined by 82% and 78%, respectively, and are now only restricted to the northern hyposaline habitats. The legislated protected area network does not cover these biodiversity hotspots. The spatial diversity maps for *q* = 0 and *q* = 2 showed similar patterns (Figs. A7 & A8, respectively). Temporally, the sea-dominated western and southern habitats in the hypersaline and mesosaline zones have undergone radical shifts in species composition since 1986 (Fig. 5).

**Table 1:** Mangroves' abundance (zone-wise total counts of each species) change during 1986–2014. Numbers in parentheses denote the percentage contribution of each species counts to the total composition (zone-wise total counts of all species). % CC (percentage compositional change) represents the difference between the percentage contribution of each species between 1986 and 2014 (for details see Section 2.6).

Species	Hyposaline zone			Mesosaline zone			Hypersaline zone		
	Absolute (relative) abundance		% CC	Absolute (relative) abundance		% CC	Absolute (relative) abundance		% CC
	1986	2014		1986	2014		1986	2014	
*Excoecaria agallocha*	9572 (46.97%)	8635 (46.59%)	−0.41%	7338 (47.95%)	9301 (56.34%)	+8.04%	9505 (85.32%)	11,558 (80.45%)	−2.94%
*Heritiera fomes*	8525 (41.43%)	8253 (44.53%)	+3.12%	7754 (50.67%)	5998 (36.33%)	−16.48%	1215 (10.91%)	1009 (7.02%)	−21.66%
*Avicennia officinalis*	568 (2.79%)	193 (1.04%)	−45.60%	1 (0.01%)	–	−100%	6 (0.05%)	4 (0.03%)	−31.84%
*Sonneratia apetala*	436 (2.14%)	116 (0.63%)	−54.73%	–	–	–	5 (0.04%)	3 (0.02%)	−36.49%
*Amoora cucullata*	350 (1.72%)	337 (1.82%)	+2.85%	51 (0.33%)	33 (0.20%)	−25.02%	57 (0.51%)	41 (0.29%)	−28.39%
*Bruguiera sexangula*	290 (1.42%)	339 (1.83%)	+12.49%	1 (0.01%)	1 (0.01%)	−3.79%	4 (0.04%)	5 (0.03%)	−1.56%
*Xylocarpus moluccensis*	198 (0.97%)	297 (1.60%)	+24.51%	38 (0.25%)	41 (0.25%)	+0.01%	76 (0.68%)	69 (0.48%)	−17.37%
*Cynometra ramiflora*	180 (0.88%)	14 (0.08%)	−84.24%	71 (0.46%)	19 (0.12%)	−60.25%	–	–	–
*Cerbera manghas*	130 (0.64%)	21 (0.11%)	−69.83%	–	–	–	–	–	–
*Talipariti tiliaceum*	59 (0.29%)	31 (0.17%)	−26.76%	–	–	–	–	–	–
*Aegiceras corniculatum*	33 (0.16%)	15 (0.08%)	−33.35%	–	–	–	–	–	–
*Excoecaria indica*	8 (0.04%)	4 (0.02%)	−29.05%	–	–	–	–	–	–
*Tamarix dioica*	8 (0.04%)	3 (0.02%)	−41.61%	–	–	–	–	–	–
*Barringtonia racemosa*	7 (0.03%)	0 (0.00%)	−100%	–	–	–	–	–	–
*Ceriops decandra*	4 (0.02%)	258 (1.39%)	+97.22%	20 (0.13%)	1098 (6.65%)	+96.15%	257 (2.31%)	1669 (11.62%)	+66.87%
*Sonneratia caseolaris*	3 (0.01%)	0 (0.00%)	−100%	–	–	–	–	–	–
*Intsia bijuga*	2 (0.01%)	3 (0.02%)	+24.51%	–	–	–	–	–	–
*Lannea coromandelica*	2 (0.01%)	0 (0.00%)	−100%	–	–	–	–	–	–
*Xylocarpus granatum*	1 (0.005%)	9 (0.05%)	+81.64%	14 (0.09%)	12 (0.07%)	−11.45%	16 (0.14%)	9 (0.06%)	−39.26%
*Pongamia pinnata*	1 (0.005%)	2 (0.01%)	+37.48%	–	–	–	–	–	–
*Syzygium fruticosum*	1 (0.005%)	1 (0.006%)	+4.75%	–	–	–	–	–	–
*Hypobathrum racemosum*	–	3 (0.02%)	+100%	0 (0.00%)	4 (0.02%)	+100%	–	–	–
*Salacia chinensis*	–	–	–	12 (0.08%)	0 (0.00%)	−100%	–	–	–
*Rhizophora mucronata*	–	–	–	0 (0.00%)	1 (0.01%)	+100%	–	–	–
*Lumnitzera racemosa*	–	–	–	3 (0.02%)	1 (0.01%)	−52.79%	–	–	–
Totals	20,378 (100%)	18,533 (100%)		15,303 (100%)	16,509 (100%)		11,141 (100%)	14,367 (100%)

### 3.3 Mangrove abundance and range dynamics

With 21 historical species presences, the hyposaline zone was the most species-rich ecological zone (Table 1). Since 1986, the percentage composition declined for all species in the hypersaline zone, except *Ceriops decandra*. Between 1986 and 2014, the contribution of *C. decandra* to the total composition increased by about 97% in the hyposaline, 96% in the mesosaline, and 67% in the hypersaline zone, indicating invasive nature of this native species. Conversely, the contribution of the specialist *Heritiera fomes* substantially declined in the meso (−16.48%) and hypersaline (−21.66%) zones.

Over the last 28 years, *C. decandra* and *Intsia bijuga* substantially expanded their range, *Cynometra ramiflora* contracted its range, *Barringtonia racemosa*, *Sonneratia caseolaris*, and *Lannea coromandelica* faced local extinction, and *Hypobathrum racemosum* newly arrived in the hyposaline zone (Fig. 6). In the mesosaline zone, *H. racemosum* and *Rhizophora mucronata* recently arrived, *C. decandra* widely expanded its range, *Amoora cucullata* and *C. ramiflora* experienced substantial range contraction, and *Avicennia officinalis* and *Salacia chinensis* faced local extinction. The range of the highly salt tolerant *C. decandra* and the pioneer species *Sonneratia apetala* considerably expanded in the hypersaline zone while the range of the salt intolerant *H. fomes* and *A. cucullata* contracted over time.

## 4 Discussion

### 4.1 Spatial structure and temporal dynamics

Salinity limits mangrove establishment, growth and development and mangrove species occupy distinct positions of coastal areas due to differential salt tolerance ability (Parida and Das, 2005). In Sundarbans, the most diverse (alpha) and distinct (least representative) mangrove communities are distributed in the hyposaline zone (Figs. 3, A4 & A5) where the soil salinity level usually stays below 2 dS m^−1^ because of adequate fresh water supply from the nearby Baleswar-Passur river system. Sarker et al. (2016) and Krauss and Ball (2013) found this salinity level suitable for coexistence of both non-halophytes (species that survive optimally in hyposaline habitats, but are likely to die at mesosaline conditions, e.g., *A. cucullata*, *I. bijuga*, *C. ramiflora* and *T. tiliaceum*) and facultative halophytes (species that grow well in hyposaline and mesosaline habitats but unable to survive at hypersaline conditions, e.g., *H. fomes*, *B. sexangula* and *X. moluccensis*). In turn, two obligate halophytes — *C. decandra* and *E. agallocha* (species that show optimum growth and reproduction in hypersaline conditions) show super-dominance in the hypersaline areas (salinity > 4 dS m^−1^). Therefore, the relatively higher alpha diversity in the hyposaline areas may be closely related to historical coexistence of both facultative and opportunistic non-halophytes under benign habitat conditions.

We detect a significant trend of increasing representativeness (decreasing heterogeneity) in the hyposaline and hypersaline SCs since 1986 (Table A2 & Figs. 3, A4 & A5). In line with that, the contribution of these SCs to the overall diversity of the ecological zones has been also declining substantially. These results indicate that the previously distinct SCs of two extreme environmental settings (low and high saline conditions) are becoming homogeneous in species composition over time. This pattern might be closely related to increasing salinity and historical industrial logging (Islam and Bhuiyan, 2018) which have resulted in local extinction, range contraction and abundance decline of many specialists (including the climax species *H. fomes*), and abundance increase and range expansion of many native obligate halophytes (i.e. native invasive, Biswas et al., 2018), particularly, *C. decandra* and *E. agallocha* (Table 1, Fig. 6). Recently, Ghosh et al. (2016) have also reported ecosystem-wide abundance decline of *H. fomes* and increasing dominance of *C. decandra* and *E. agallocha* in the degraded Indian Sundarbans since 1977.

Surprisingly, in the last 15 years, alpha diversity sharply increased in the SCs of all the ecological zones (Fig. 3a), particularly in the southern meso- and western hypersaline areas (Fig. 4a) where salinity fluctuation is highest and often the salinity level remains high for longer in the dry season (November–April), inhibiting the regeneration process and inducing disease outbreaks in many specialists such as *H. fomes* and *A. cucullata* (Mukhopadhyay et al., 2015). Our temporal beta diversity analyses (Fig. 6a–c) further detected that these areas have experienced radical shifts in species composition since 1986, mostly due to the abundance increase and range expansion of the native invasive species *C. decandra* (Table 1, Fig. 6). This scenario is in agreement with the basic homogenization theory that assumes that changes in alpha diversity can be independent of between-habitat homogenization because replacement of locally distinct SCs (containing rare species) with generalists or invasive species would reduce heterogeneity, but the SCs may remain the same/increase in species number (Olden and Poff, 2003; Smart et al., 2006). Over the four historical time points, the SCs of the mesosaline zone shows stable and higher representativeness (i.e. homogeneity in species composition) compared to the SCs of the other ecological zones (Table A2). This compositional stability may be due to prevailing intermediate environmental conditions in this zone which support both facultative and obligate halophytes. Previous studies (Flowers et al., 2010; Krauss and Ball, 2013) on other mangrove systems also reported stable forest structure at moderate salinity concentrations.

### 4.2 Spatial diversity maps

Our diversity maps (Figs. 4, A7 & A8) indicate that both historical and current (alpha) biodiversity hotspots are confined to the northern hyposaline habitats (specifically the Kalabogi region). These upstream habitats are only inundated by spring high tides so receive the lowest amount of saltwater from the Bay of Bengal. These habitats now also support the most diverse (alpha) and unrepresentative (i.e. distinct) SCs comprising the remaining assemblages of the unique *H. fomes – B. sexangula – X. moluccensis* forest type (Islam et al., 2016). Except for the upstream northern habitats, the mangrove assemblages of the rest of the ecosystem are currently homogeneous which may be related to a gradual decline in spatial coverage of the distinct communities since 1986.

Note that the semivariograms of the biodiversity indices (Figs. A1, A2, and A3) and cross validation of our kriged predictions (Table A2) indicate that the variation in alpha diversity between sampling locations is relatively better explained by their proximity to each other and the prediction accuracy of the alpha diversity indices is fairly consistent under different time points (1986, 1994, 1999 and 2014), compared to the beta and gamma diversity indices, suggesting a fair amount of uncertainty remains in predicting beta and gamma diversity using direct interpolation technique. Therefore, future studies should consider incorporating fine-scale variability in habitat conditions to produce more reliable beta and gamma diversity maps for the region.

### 4.3 Conservation and management implications

The world's largest mangrove protected area network (PAN) comprising three wildlife sanctuaries are operational in the Bangladesh Sundarbans, covering 52.84% (3179.50 km^2^) of the ecosystem. Our maps (Figs. 4, A7 & A8) reveal that the mangrove tree diversity hotspots are located outside the PAN. Although the wildlife sanctuaries were established under the Wildlife (Conservation and Security) Act, 2012 to confirm completely undisturbed habitats for wildlife and plants, the key focus was to protect the Royal Bengal tiger, the Sundarbans's flagship species, and its prey populations. Opportunistic felling of valuable timber-yielding species such as *H. fomes* and *X. moluccensis* is common (Iftekhar and Saenger, 2008). This forest exploitation is directly associated with the habitat loss of many mangrove-dwelling animals including the Royal Bengal tiger. Hence, in addition to protecting animals, serious consideration should be given to protect the threatened mangrove trees via bringing the surviving biodiversity hotspots under protected area management. The ability of the legislated PAN to conserve biodiversity has already been criticized because of inadequate funds and patrolling workforce (Hossain et al., 2016). Therefore, the success of the current and future biodiversity protection measures in the Sundarbans is likely to depend on the legislative reformation and logistics support provided by the government.

Mangrove enhancement (reducing biotic and abiotic stresses that caused mangroves' population decline) and mangrove restoration (restoring specific areas where certain mangrove species previously existed) initiatives are regularly taken in the tropical coastal regions to enhance species resistance and resilience to climate change and to offset predicted losses from climate change impacts (Lewis, 2005). However, an inadequate understanding of spatial and temporal dynamics in mangrove diversity and composition under different environmental conditions has resulted in unsuccessful mangrove enhancement and restoration projects in many countries (Romañach et al., 2018), including the Sundarbans (Islam et al., 2014). Sea level rise is likely to have severe impacts on the Sundarbans and the ecosystem may lose 10% - 23% of its present area by 2100 (Payo et al., 2016) with alteration to coastal geomorphology and hydrology (Banerjee et al., 2017; Wahid et al., 2007). Therefore, given the severity of these future environmental impacts on the Sundarbans, tracking how mangrove communities have changed over time and identifying the existing and future environmental stressors of mangrove diversity and community composition are important. Recently, Sarker et al. (2019) provided a quantitative understanding of the drivers shaping spatial distributions of mangrove biodiversity in the Sundarbans. They found that several environmental drivers, biotic interactions and historical events have combined effects on the spatial variability in mangrove diversity and community composition. Specifically, they identified salinity intrusion, historical harvesting, siltation, tree disease and increasing soil alkalinity as the dominant stressors responsible for mangrove biodiversity loss in the Sundarbans. We believe that Sarker et al.'s (2019) findings and our biodiversity maps – containing detailed information about which areas have lost biodiversity since 1986 and which areas (sea-dominated western and southern habitats) have experienced radical shifts in species composition due to invasion of salt-tolerant generalists – can together guide the BFD in designing science-driven mangrove enhancement and restoration plans. Our species-specific results on the abundance and range dynamics (1986–2014) in the hypo-, meso-, and hypersaline habitats and Sarker et al.'s (2016) habitat suitability models and maps for Sundarbans' mangroves, together, can further help the BFD in selecting appropriate species and suitable habitats for future reforestation initiatives. We also suggest prioritisation of rare endemic species in the future mangrove enrichment plantation and ANR (Assisted Natural Regeneration) programs.

We detect that already many mangrove tree species have faced local extinction and the abundance and geographic range of many of the remaining species have substantially declined over the last three decades (Table 1 & Fig. 6). Hence, we suggest that ongoing and future mangrove protection and conservation initiatives should immediately focus on the threatened (e.g., *H. fomes* and *X. moluccensis*) and the rare endemics (e.g., *C. ramiflora*, *Cerbera manghas* and *A. cucullata*) because further exploitation may push them to the brink of extinction. Further, the existing PSP network does not cover a number of mangrove tree species (e.g., *Avicennia marina*, *Bruguiera gymnorhiza*) recorded earlier (Aziz and Paul, 2015). Therefore, we suggest for an extension of the existing PSP network to include the habitats of these missing tree species.

Bangladesh has signed and ratified the Convention on Biological Diversity, World Heritage Convention, and Ramsar Convention and also enacted the ‘Bangladesh Biodiversity Act 2017’ to minimize biodiversity loss. To improve natural resource governance and biodiversity conservation, BFD has also introduced the co-management approach involving all the stakeholders within the Sundarbans Impact Zone (a 10 km band impact zone surrounding the Sundarbans with a human settlement of 3.5 million partly forest-dependent people, Aziz and Paul, 2015). Despite a full logging ban being in operation since 1989, opportunistic felling of valuable tree species is common in the Sundarbans. Bangladesh has recently developed the Biodiversity National Assessment and Program of Action 2020 to halt further degradation of biological resources. The BFD has started implementing a SMART (Spatial Monitoring and Reporting Tool) patrol management system in the Sundarbans for adequate forest protection. We believe our mangrove diversity maps may guide these valuable protection and monitoring initiatives of BFD to combat illicit logging through recording mangrove biodiversity changes or predicting changes and identifying areas (or species) that may be most affected by future human interventions.

Highly productive mangrove ecosystems are well known for providing a variety of ecosystem services. The Sundarbans offers a range of ecosystem services including provisioning services (e.g., timber and food products such as fish), regulatory services (e.g., carbon sequestration and protection from cyclones) and cultural services (e.g., mangrove tourism) (Islam et al., 2018; Uddin et al., 2013). Our result – that Sundarbans' mangrove communities have been becoming homogenous in species composition since 1986 (Figs. 3, A4 & A5) due to the local extinction, range contraction and abundance decline of many specialists, and the increases in the abundance and range of a few generalists (Table 1 and Fig. 6) – have serious consequences for these valuable ecosystem services. For example, Rahman et al. (2015) found *H. fomes*-dominated forest communities store the highest amount of ecosystem carbon (360.1 ± 22.71 Mg C ha^−1^), while the *E. agallocha* – *C. decandra*-dominated communities store the least amount of ecosystem carbon (159.49 ± 6.86 Mg C ha^−1^). We therefore believe that the reduction in abundances of the high timber-yielding and specialist *H. fomes* in the mesosaline and hypersaline zones over the last 28 years (Table 1) may have resulted in a significant carbon stock loss in these zones. Indeed, in terms of the salinity zones, the hyposaline zone shows a twofold increase in ecosystem carbon stock (336.09 ± 14.74 Mg C ha^−1^) than the hypersaline zone (Rahman et al., 2015). Sarker et al. (2016) also observed a sharp negative response of *H. fomes* abundance and a sharp positive response of *E. agallocha* abundance and *C. decandra* abundance to increasing salinity. Their species-specific density maps show a limited expanse of the highly biomass-productive *H. fomes* in the eastern hyposaline zone, a widespread distribution of the less productive *E. agallocha* in the entire Sundarbans and super-dominance of the dwarf mangrove species *C. decandra* in the western and southern hypersaline zones. All these findings lead us to conclude that further degradation (e.g., increased salinity, siltation, disease-outbreaks) of the ecosystem under the projected sea level rise (Karim and Mimura, 2008) could push the highly productive specialists (e.g., *H. fomes*) over the brink of extinction, and may provide opportunities for the less productive generalists/disturbance specialists (e.g., *E. agallocha* and *C. decandra*) to colonize the degraded habitats (Sarker et al., 2016). This could transform the remaining heterogeneous mangrove communities into species-poor homogeneous patches which may result in an ecosystem-wide drop in carbon stock (Rahman et al., 2015) and fish production (Ahmed et al., 2017), a limited ability of the ecosystem to provide buffer against cyclones and tsunamis (Sandilyan and Kathiresan, 2015), and habitat loss of many globally endangered animals including the Royal Bengal tiger (Aziz et al., 2013).

Bangladesh Forestry Master Plan 2017–2036 (BFD, 2016), the existing Integrated Resource Management Plan (IRMP) 2010–2020 for the entire Sundarbans (BFD, 2010), and the specific five-year management plans for the wildlife sanctuaries (Sundarbans East and South) mainly aim to help BFD in sustaining multiple functions and ecosystem services simultaneously through protecting and enhancing biodiversity. However, these plans have been criticized for an incomprehensive representation of biodiversity information and functional role of mangroves (Islam et al., 2016). Our spatial biodiversity maps (Fig. 4, Fig. 5, A7 & A8) and species level analysis of abundance and range dynamics (Table 1 & Fig. 6), collectively, pinpoint the areas that experienced biodiversity loss/gain and became homogenous/heterogenous, and also detail how population size and geographic coverage of the species have changed across the ecological zones since 1986. Given the severe consequences of historical and future homogenization of Sundarbans's mangrove communities on ecosystem functions and services (Plas et al., 2016), these baseline information can guide BFD to develop a more comprehensive management plan for the Sundarbans. The Bangladeshi government has been looking at new ways to protect and conserve Sundarbans through different climate change mitigation approaches such as ‘Reducing Emissions from Deforestation and forest Degradation (REDD^+^)’ (Ahmed and Glaser, 2016). A baseline assessment of biodiversity is essential in order to participate in the UNFCCC's REDD^+^ program (Ahmed et al., 2017). Recently, the government has also initiated a Collaborative REDD/IFM (Improved Forest Management) Sundarbans Project (CRISP) to conserve mangrove forests for a total CO_2_ emissions reduction of about 6.4 million tons over a 30-year period (Ahmed et al., 2017). Our spatially-explicit baseline biodiversity information can contribute to successful implementation of these initiatives for enhancing carbon stock (through biodiversity conservation) and also for generating economic benefit for the country (Razzaque, 2017).

## 5 Conclusions

Using extensive data from 28 years of mangrove surveys, we created the first baseline mangrove tree diversity maps for the world's largest mangrove ecosystem – the Sundarbans, and uncovered that this world heritage ecosystem is becoming homogenous in species composition because of spatial contraction of the distinct and diverse mangrove communities over time. Our maps reveal that the surviving mangrove biodiversity hotspots are located outside the existing PAN. We advocate bringing these hotspots under protected area management. We detect the highest turnover in species composition in the western and southern hypersaline areas because of abundance increase and range expansion of the invasive species *Ceriops decandra* and local extinction or range contraction of endemics including the globally endangered *Heritiera fomes*. These novel results can guide future mangrove research, biodiversity assessment and monitoring programs in the Sundarbans. The BFD can readily use our tree diversity maps with complementary information on the abundance and range dynamics of the species in their ongoing and future mangrove enhancement, restoration and protection initiatives, and also use the spatial biodiversity information to develop a more comprehensive management plan for the Sundarbans. The presence of many rare tree species and the absence of a number of previously recorded tree species in our long-term datasets also advocate for the extension of the current PSP network. Due to the unavailability of long-term environmental data for the Sundarbans, we used purely spatial technique (kriging) to make predictions for the diversity measures. The projected sea level rise may alter the regional hydrology and habitat conditions with associated changes in species composition. We therefore recommend accounting for spatial and temporal changes in habitat conditions in future biodiversity studies to improve our understanding of how mangrove community composition and abundance may shift under future environmental conditions.

## Appendix A Supplementary data

Supplementary material Image 1
