# Supplementary material for: 1980s–2010s: The world's largest mangrove ecosystem is becoming homogeneous
Source: Biol Conserv. 2019 Aug;236:79–91. doi: 10.1016/j.biocon.2019.05.011 (PMC6716549; doi:10.1016/j.biocon.2019.05.011)
Supplement: Supplementary file 1 — Supplementary material [file mmc1.docx]

**Appendices**

***Table A1.*** *Test-statistics for the One-way repeated measures analyses of variance (ANOVA) representing to what extent subcommunity alpha, beta and gamma diversity (for q = 0, 1 and 2, and for two metacommunity levels: ecological zone as metacommunity and whole ecosystem as metacommunity) varied in the ecological zones of the Sundarbans over the last 28 years.*

| Diversity types | Ecological zone as Metacommunity | | | | |
| --- | --- | --- | --- | --- | --- |
|  | Source | Sum of squares | Mean Squares | F value | P - value |
| ${}^{0}\overline{\alpha}$ | Years | 3727.08 | 1242.36 | 984.18 | < 0.001 |
|  | Ecological zones | 7365.12 | 3682.56 | 2917.28 | < 0.001 |
|  | Years : Ecological zones | 275.22 | 45.87 | 36.34 | < 0.001 |
| ${}^{1}\overline{\alpha}$ | Years | 555.56 | 185.19 | 873.85 | < 0.001 |
|  | Ecological zones | 3139.38 | 1569.69 | 7406.96 | < 0.001 |
|  | Years : Ecological zones | 243.69 | 40.61 | 191.65 | < 0.001 |
| ${}^{2}\overline{\alpha}$ | Years | 229.28 | 76.43 | 482.17 | < 0.001 |
|  | Ecological zones | 2138.43 | 1069.22 | 6745.61 | < 0.001 |
|  | Years : Ecological zones | 121.35 | 20.22 | 127.59 | < 0.001 |
| ${}^{0}\overline{\rho}$ | Years | 2.36 | 0.79 | 156.29 | < 0.001 |
|  | Ecological zones | 46.78 | 23.39 | 4641.23 | < 0.001 |
|  | Years : Ecological zones | 3.80 | 0.63 | 125.56 | < 0.001 |
| ${}^{1}\overline{\rho}$ | Years | 11.33 | 3.78 | 157.07 | < 0.001 |
|  | Ecological zones | 242.22 | 121.11 | 5037.43 | < 0.001 |
|  | Years : Ecological zones | 7.53 | 1.26 | 52.21 | < 0.001 |
| ${}^{2}\overline{\rho}$ | Years | 12.23 | 4.08 | 102.73 | < 0.001 |
|  | Ecological zones | 323.33 | 161.66 | 4072.72 | < 0.001 |
|  | Years : Ecological zones | 5.04 | 0.84 | 21.15 | < 0.001 |
| ${}^{0}\gamma$ | Years | 62663.60 | 20887.87 | 50.03 | < 0.001 |
|  | Ecological zones | 868079.68 | 434039.84 | 1039.57 | < 0.001 |
|  | Years : Ecological zones | 74700.92 | 12450.15 | 29.82 | < 0.001 |
| ${}^{1}\gamma$ | Years | 10782.79 | 3594.26 | 117.14 | < 0.001 |
|  | Ecological zones | 85638.06 | 42819.03 | 1395.53 | < 0.001 |
|  | Years : Ecological zones | 13132.82 | 2188.80 | 71.34 | < 0.001 |
| ${}^{2}\gamma$ | Years | 3888.72 | 1296.24 | 120.33 | < 0.001 |
|  | Ecological zones | 27544.63 | 13772.31 | 1278.46 | < 0.001 |
|  | Years : Ecological zones | 5115.91 | 852.65 | 79.15 | < 0.001 |
|  |  | Whole ecosystem as Metacommunity | | | |
| ${}^{0}\overline{\rho}$ | Years | 0.53 | 0.18 | 17.79 | < 0.001 |
|  | Ecological zones | 29.80 | 14.90 | 1503.74 | < 0.001 |
|  | Years : Ecological zones | 7.32 | 1.22 | 123.20 | < 0.001 |
| ${}^{1}\overline{\rho}$ | Years | 6.59 | 2.20 | 86.21 | < 0.001 |
|  | Ecological zones | 201.20 | 100.60 | 3950.12 | < 0.001 |
|  | Years : Ecological zones | 11.44 | 1.91 | 74.87 | < 0.001 |
| ${}^{2}\overline{\rho}$ | Years | 8.33 | 2.78 | 73.51 | < 0.001 |
|  | Ecological zones | 298.18 | 149.09 | 3945.22 | < 0.001 |
|  | Years : Ecological zones | 19.46 | 3.24 | 85.84 | < 0.001 |
| ${}^{0}\gamma$ | Years | 394317.67 | 131439.22 | 44.74 | < 0.001 |
|  | Ecological zones | 14665665.21 | 7332832.61 | 2496.06 | < 0.001 |
|  | Years : Ecological zones | 549522.63 | 91587.10 | 31.18 | < 0.001 |
| ${}^{1}\gamma$ | Years | 88793.91 | 29597.97 | 110.95 | < 0.001 |
|  | Ecological zones | 501023.69 | 250511.85 | 939.02 | < 0.001 |
|  | Years : Ecological zones | 102786.47 | 17131.08 | 64.21 | < 0.001 |
| ${}^{2}\gamma$ | Years | 11717.40 | 3905.80 | 67.70 | < 0.001 |
|  | Ecological zones | 68557.55 | 34278.77 | 594.15 | < 0.001 |
|  | Years : Ecological zones | 15916.45 | 2652.74 | 45.98 | < 0.001 |

***Table A2.*** *Results of* *post-hoc tests using multiple pairwise comparisons between the subcommunity alpha, beta and gamma diversity (for q = 0, 1 and 2, and for two metacommunity levels: ecological zone as metacommunity and whole ecosystem as metacommunity) representing to what extent the diversity components differed within and between the ecological zones (i.e., hypo-, meso- and hypersaline) in the Sundarbans over the four historical time points: 1986, 1994, 1999 and 2014.*

|  | Ecological zone as Metacommunity | | | | |
| --- | --- | --- | --- | --- | --- |
| Diversity types |  | Ecological zones in four census periods | Mean difference | t – ratio | P – value |
| ${}^{0}\overline{\alpha}$ | 1 | 1986,Hypersaline - 1994,Hypersaline | 0.47 | 16.09 | < 0.001 |
|  | 2 | 1986,Hypersaline - 1999,Hypersaline | 0.67 | 22.98 | < 0.001 |
|  | 3 | 1986,Hypersaline - 2014,Hypersaline | -0.33 | -11.49 | < 0.001 |
|  | 4 | 1986,Hypersaline - 1986,Hyposaline | -0.98 | -33.75 | < 0.001 |
|  | 5 | 1986,Hypersaline - 1994,Hyposaline | -0.55 | -19.04 | < 0.001 |
|  | 6 | 1986,Hypersaline - 1999,Hyposaline | -0.51 | -17.50 | < 0.001 |
|  | 7 | 1986,Hypersaline - 2014,Hyposaline | -1.00 | -34.44 | < 0.001 |
|  | 8 | 1986,Hypersaline - 1986,Mesosaline | 0.10 | 3.45 | < 0.05 |
|  | 9 | 1986,Hypersaline - 1994,Mesosaline | 0.43 | 14.94 | < 0.001 |
|  | 10 | 1986,Hypersaline - 1999,Mesosaline | 0.60 | 20.68 | < 0.001 |
|  | 11 | 1986,Hypersaline - 2014,Mesosaline | -0.33 | -11.49 | < 0.001 |
|  | 12 | 1994,Hypersaline - 1999,Hypersaline | 0.20 | 6.89 | < 0.001 |
|  | 13 | 1994,Hypersaline - 2014,Hypersaline | -0.80 | -27.58 | < 0.001 |
|  | 14 | 1994,Hypersaline - 1986,Hyposaline | -1.45 | -49.83 | < 0.001 |
|  | 15 | 1994,Hypersaline - 1994,Hyposaline | -1.02 | -35.13 | < 0.001 |
|  | 16 | 1994,Hypersaline - 1999,Hyposaline | -0.97 | -33.59 | < 0.001 |
|  | 17 | 1994,Hypersaline - 2014,Hyposaline | -1.47 | -50.52 | < 0.001 |
|  | 18 | 1994,Hypersaline - 1986,Mesosaline | -0.37 | -12.64 | < 0.001 |
|  | 19 | 1994,Hypersaline - 1994,Mesosaline | -0.03 | -1.15 | 0.992 |
|  | 20 | 1994,Hypersaline - 1999,Mesosaline | 0.13 | 4.60 | < 0.01 |
|  | 21 | 1994,Hypersaline - 2014,Mesosaline | -0.80 | -27.58 | < 0.001 |
|  | 22 | 1999,Hypersaline - 2014,Hypersaline | -1.00 | -34.47 | < 0.001 |
|  | 23 | 1999,Hypersaline - 1986,Hyposaline | -1.65 | -56.73 | < 0.001 |
|  | 24 | 1999,Hypersaline - 1994,Hyposaline | -1.22 | -42.02 | < 0.001 |
|  | 25 | 1999,Hypersaline - 1999,Hyposaline | -1.17 | -40.48 | < 0.001 |
|  | 26 | 1999,Hypersaline - 2014,Hyposaline | -1.67 | -57.42 | < 0.001 |
|  | 27 | 1999,Hypersaline - 1986,Mesosaline | -0.57 | -19.53 | < 0.001 |
|  | 28 | 1999,Hypersaline - 1994,Mesosaline | -0.23 | -8.04 | < 0.001 |
|  | 29 | 1999,Hypersaline - 1999,Mesosaline | -0.07 | -2.30 | 0.478 |
|  | 30 | 1999,Hypersaline - 2014,Mesosaline | -1.00 | -34.47 | < 0.001 |
|  | 31 | 2014,Hypersaline - 1986,Hyposaline | -0.65 | -22.26 | < 0.001 |
|  | 32 | 2014,Hypersaline - 1994,Hyposaline | -0.22 | -7.55 | < 0.001 |
|  | 33 | 2014,Hypersaline - 1999,Hyposaline | -0.17 | -6.01 | < 0.001 |
|  | 34 | 2014,Hypersaline - 2014,Hyposaline | -0.67 | -22.95 | < 0.001 |
|  | 35 | 2014,Hypersaline - 1986,Mesosaline | 0.43 | 14.94 | < 0.001 |
|  | 36 | 2014,Hypersaline - 1994,Mesosaline | 0.77 | 26.43 | < 0.001 |
|  | 37 | 2014,Hypersaline - 1999,Mesosaline | 0.93 | 32.17 | < 0.001 |
|  | 38 | 2014,Hypersaline - 2014,Mesosaline | 0.00 | 0.00 | 0.925 |
|  | 39 | 1986,Hyposaline - 1994,Hyposaline | 0.43 | 14.71 | < 0.001 |
|  | 40 | 1986,Hyposaline - 1999,Hyposaline | 0.47 | 16.25 | < 0.001 |
|  | 41 | 1986,Hyposaline - 2014,Hyposaline | -0.02 | -0.69 | 0.965 |
|  | 42 | 1986,Hyposaline - 1986,Mesosaline | 1.08 | 37.19 | < 0.001 |
|  | 43 | 1986,Hyposaline - 1994,Mesosaline | 1.41 | 48.69 | < 0.001 |
|  | 44 | 1986,Hyposaline - 1999,Mesosaline | 1.58 | 54.43 | < 0.001 |
|  | 45 | 1986,Hyposaline - 2014,Mesosaline | 0.65 | 22.26 | < 0.001 |
|  | 46 | 1994,Hyposaline - 1999,Hyposaline | 0.04 | 1.54 | 0.983 |
|  | 47 | 1994,Hyposaline - 2014,Hyposaline | -0.45 | -15.40 | < 0.001 |
|  | 48 | 1994,Hyposaline - 1986,Mesosaline | 0.65 | 22.49 | < 0.001 |
|  | 49 | 1994,Hyposaline - 1994,Mesosaline | 0.99 | 33.98 | < 0.001 |
|  | 50 | 1994,Hyposaline - 1999,Mesosaline | 1.15 | 39.72 | < 0.001 |
|  | 51 | 1994,Hyposaline - 2014,Mesosaline | 0.22 | 7.55 | < 0.001 |
|  | 52 | 1999,Hyposaline - 2014,Hyposaline | -0.49 | -16.94 | < 0.001 |
|  | 53 | 1999,Hyposaline - 1986,Mesosaline | 0.61 | 20.95 | < 0.001 |
|  | 54 | 1999,Hyposaline - 1994,Mesosaline | 0.94 | 32.44 | < 0.001 |
|  | 55 | 1999,Hyposaline - 1999,Mesosaline | 1.11 | 38.18 | < 0.001 |
|  | 56 | 1999,Hyposaline - 2014,Mesosaline | 0.17 | 6.01 | < 0.001 |
|  | 57 | 2014,Hyposaline - 1986,Mesosaline | 1.10 | 37.88 | < 0.001 |
|  | 58 | 2014,Hyposaline - 1994,Mesosaline | 1.43 | 49.37 | < 0.001 |
|  | 59 | 2014,Hyposaline - 1999,Mesosaline | 1.60 | 55.12 | < 0.001 |
|  | 60 | 2014,Hyposaline - 2014,Mesosaline | 0.67 | 22.95 | < 0.001 |
|  | 61 | 1986,Mesosaline - 1994,Mesosaline | 0.33 | 11.49 | < 0.001 |
|  | 62 | 1986,Mesosaline - 1999,Mesosaline | 0.50 | 17.24 | < 0.001 |
|  | 63 | 1986,Mesosaline - 2014,Mesosaline | -0.43 | -14.94 | < 0.001 |
|  | 64 | 1994,Mesosaline - 1999,Mesosaline | 0.17 | 5.75 | < 0.001 |
|  | 65 | 1994,Mesosaline - 2014,Mesosaline | -0.77 | -26.43 | < 0.001 |
|  | 66 | 1999,Mesosaline - 2014,Mesosaline | -0.93 | -32.17 | < 0.001 |
| ${}^{1}\overline{\alpha}$ | 1 | 1986,Hypersaline - 1994,Hypersaline | 0.09 | 7.72 | < 0.001 |
|  | 2 | 1986,Hypersaline - 1999,Hypersaline | 0.15 | 12.93 | < 0.001 |
|  | 3 | 1986,Hypersaline - 2014,Hypersaline | -0.38 | -31.75 | < 0.001 |
|  | 4 | 1986,Hypersaline - 1986,Hyposaline | -0.79 | -66.42 | < 0.001 |
|  | 5 | 1986,Hypersaline - 1994,Hyposaline | -0.72 | -60.22 | < 0.001 |
|  | 6 | 1986,Hypersaline - 1999,Hyposaline | -0.72 | -60.61 | < 0.001 |
|  | 7 | 1986,Hypersaline - 2014,Hyposaline | -0.76 | -64.34 | < 0.001 |
|  | 8 | 1986,Hypersaline - 1986,Mesosaline | -0.42 | -35.31 | < 0.001 |
|  | 9 | 1986,Hypersaline - 1994,Mesosaline | -0.41 | -34.29 | < 0.001 |
|  | 10 | 1986,Hypersaline - 1999,Mesosaline | -0.37 | -31.33 | < 0.001 |
|  | 11 | 1986,Hypersaline - 2014,Mesosaline | -0.75 | -62.99 | < 0.001 |
|  | 12 | 1994,Hypersaline - 1999,Hypersaline | 0.06 | 5.21 | < 0.001 |
|  | 13 | 1994,Hypersaline - 2014,Hypersaline | -0.47 | -39.47 | < 0.001 |
|  | 14 | 1994,Hypersaline - 1986,Hyposaline | -0.88 | -74.14 | < 0.001 |
|  | 15 | 1994,Hypersaline - 1994,Hyposaline | -0.81 | -67.94 | < 0.001 |
|  | 16 | 1994,Hypersaline - 1999,Hyposaline | -0.81 | -68.33 | < 0.001 |
|  | 17 | 1994,Hypersaline - 2014,Hyposaline | -0.86 | -72.06 | < 0.001 |
|  | 18 | 1994,Hypersaline - 1986,Mesosaline | -0.51 | -43.03 | < 0.001 |
|  | 19 | 1994,Hypersaline - 1994,Mesosaline | -0.50 | -42.01 | < 0.001 |
|  | 20 | 1994,Hypersaline - 1999,Mesosaline | -0.46 | -39.05 | < 0.001 |
|  | 21 | 1994,Hypersaline - 2014,Mesosaline | -0.84 | -70.71 | < 0.001 |
|  | 22 | 1999,Hypersaline - 2014,Hypersaline | -0.53 | -44.68 | < 0.001 |
|  | 23 | 1999,Hypersaline - 1986,Hyposaline | -0.94 | -79.36 | < 0.001 |
|  | 24 | 1999,Hypersaline - 1994,Hyposaline | -0.87 | -73.16 | < 0.001 |
|  | 25 | 1999,Hypersaline - 1999,Hyposaline | -0.87 | -73.54 | < 0.001 |
|  | 26 | 1999,Hypersaline - 2014,Hyposaline | -0.92 | -77.28 | < 0.001 |
|  | 27 | 1999,Hypersaline - 1986,Mesosaline | -0.57 | -48.25 | < 0.001 |
|  | 28 | 1999,Hypersaline - 1994,Mesosaline | -0.56 | -47.23 | < 0.001 |
|  | 29 | 1999,Hypersaline - 1999,Mesosaline | -0.53 | -44.26 | < 0.001 |
|  | 30 | 1999,Hypersaline - 2014,Mesosaline | -0.90 | -75.93 | < 0.001 |
|  | 31 | 2014,Hypersaline - 1986,Hyposaline | -0.41 | -34.67 | < 0.001 |
|  | 32 | 2014,Hypersaline - 1994,Hyposaline | -0.34 | -28.47 | < 0.001 |
|  | 33 | 2014,Hypersaline - 1999,Hyposaline | -0.34 | -28.86 | < 0.001 |
|  | 34 | 2014,Hypersaline - 2014,Hyposaline | -0.39 | -32.59 | < 0.001 |
|  | 35 | 2014,Hypersaline - 1986,Mesosaline | -0.04 | -3.56 | <0.05 |
|  | 36 | 2014,Hypersaline - 1994,Mesosaline | -0.03 | -2.54 | 0.315 |
|  | 37 | 2014,Hypersaline - 1999,Mesosaline | 0.01 | 0.42 | 0.834 |
|  | 38 | 2014,Hypersaline - 2014,Mesosaline | -0.37 | -31.24 | < 0.001 |
|  | 39 | 1986,Hyposaline - 1994,Hyposaline | 0.07 | 6.20 | < 0.001 |
|  | 40 | 1986,Hyposaline - 1999,Hyposaline | 0.07 | 5.82 | < 0.001 |
|  | 41 | 1986,Hyposaline - 2014,Hyposaline | 0.02 | 2.08 | 0.635 |
|  | 42 | 1986,Hyposaline - 1986,Mesosaline | 0.37 | 31.11 | < 0.001 |
|  | 43 | 1986,Hyposaline - 1994,Mesosaline | 0.38 | 32.13 | < 0.001 |
|  | 44 | 1986,Hyposaline - 1999,Mesosaline | 0.42 | 35.10 | < 0.001 |
|  | 45 | 1986,Hyposaline - 2014,Mesosaline | 0.04 | 3.43 | < 0.05 |
|  | 46 | 1994,Hyposaline - 1999,Hyposaline | 0.00 | -0.39 | 0.895 |
|  | 47 | 1994,Hyposaline - 2014,Hyposaline | -0.05 | -4.12 | < 0.01 |
|  | 48 | 1994,Hyposaline - 1986,Mesosaline | 0.30 | 24.91 | < 0.001 |
|  | 49 | 1994,Hyposaline - 1994,Mesosaline | 0.31 | 25.93 | < 0.001 |
|  | 50 | 1994,Hyposaline - 1999,Mesosaline | 0.34 | 28.90 | < 0.001 |
|  | 51 | 1994,Hyposaline - 2014,Mesosaline | -0.03 | -2.77 | 0.192 |
|  | 52 | 1999,Hyposaline - 2014,Hyposaline | -0.04 | -3.73 | < 0.05 |
|  | 53 | 1999,Hyposaline - 1986,Mesosaline | 0.30 | 25.29 | < 0.001 |
|  | 54 | 1999,Hyposaline - 1994,Mesosaline | 0.31 | 26.32 | < 0.001 |
|  | 55 | 1999,Hyposaline - 1999,Mesosaline | 0.35 | 29.28 | < 0.001 |
|  | 56 | 1999,Hyposaline - 2014,Mesosaline | -0.03 | -2.39 | 0.415 |
|  | 57 | 2014,Hyposaline - 1986,Mesosaline | 0.35 | 29.03 | < 0.001 |
|  | 58 | 2014,Hyposaline - 1994,Mesosaline | 0.36 | 30.05 | < 0.001 |
|  | 59 | 2014,Hyposaline - 1999,Mesosaline | 0.39 | 33.01 | < 0.001 |
|  | 60 | 2014,Hyposaline - 2014,Mesosaline | 0.02 | 1.35 | 0.942 |
|  | 61 | 1986,Mesosaline - 1994,Mesosaline | 0.01 | 1.02 | 0.933 |
|  | 62 | 1986,Mesosaline - 1999,Mesosaline | 0.05 | 3.99 | < 0.01 |
|  | 63 | 1986,Mesosaline - 2014,Mesosaline | -0.33 | -27.68 | < 0.001 |
|  | 64 | 1994,Mesosaline - 1999,Mesosaline | 0.04 | 2.97 | 0.119 |
|  | 65 | 1994,Mesosaline - 2014,Mesosaline | -0.34 | -28.70 | < 0.001 |
|  | 66 | 1999,Mesosaline - 2014,Mesosaline | -0.38 | -31.67 | < 0.001 |
| ${}^{2}\overline{\alpha}$ | 1 | 1986,Hypersaline - 1994,Hypersaline | 0.04 | 3.90 | < 0.01 |
|  | 2 | 1986,Hypersaline - 1999,Hypersaline | 0.08 | 7.42 | < 0.001 |
|  | 3 | 1986,Hypersaline - 2014,Hypersaline | -0.27 | -26.18 | < 0.001 |
|  | 4 | 1986,Hypersaline - 1986,Hyposaline | -0.63 | -61.34 | < 0.001 |
|  | 5 | 1986,Hypersaline - 1994,Hyposaline | -0.57 | -55.86 | < 0.001 |
|  | 6 | 1986,Hypersaline - 1999,Hyposaline | -0.59 | -57.60 | < 0.001 |
|  | 7 | 1986,Hypersaline - 2014,Hyposaline | -0.60 | -58.37 | < 0.001 |
|  | 8 | 1986,Hypersaline - 1986,Mesosaline | -0.44 | -42.63 | < 0.001 |
|  | 9 | 1986,Hypersaline - 1994,Mesosaline | -0.45 | -43.64 | < 0.001 |
|  | 10 | 1986,Hypersaline - 1999,Mesosaline | -0.42 | -41.28 | < 0.001 |
|  | 11 | 1986,Hypersaline - 2014,Mesosaline | -0.67 | -65.23 | < 0.001 |
|  | 12 | 1994,Hypersaline - 1999,Hypersaline | 0.04 | 3.52 | < 0.05 |
|  | 13 | 1994,Hypersaline - 2014,Hypersaline | -0.31 | -30.08 | < 0.001 |
|  | 14 | 1994,Hypersaline - 1986,Hyposaline | -0.67 | -65.24 | < 0.001 |
|  | 15 | 1994,Hypersaline - 1994,Hyposaline | -0.61 | -59.76 | < 0.001 |
|  | 16 | 1994,Hypersaline - 1999,Hyposaline | -0.63 | -61.50 | < 0.001 |
|  | 17 | 1994,Hypersaline - 2014,Hyposaline | -0.64 | -62.27 | < 0.001 |
|  | 18 | 1994,Hypersaline - 1986,Mesosaline | -0.48 | -46.53 | < 0.001 |
|  | 19 | 1994,Hypersaline - 1994,Mesosaline | -0.49 | -47.54 | < 0.001 |
|  | 20 | 1994,Hypersaline - 1999,Mesosaline | -0.46 | -45.18 | < 0.001 |
|  | 21 | 1994,Hypersaline - 2014,Mesosaline | -0.71 | -69.13 | < 0.001 |
|  | 22 | 1999,Hypersaline - 2014,Hypersaline | -0.35 | -33.60 | < 0.001 |
|  | 23 | 1999,Hypersaline - 1986,Hyposaline | -0.71 | -68.76 | < 0.001 |
|  | 24 | 1999,Hypersaline - 1994,Hyposaline | -0.65 | -63.28 | < 0.001 |
|  | 25 | 1999,Hypersaline - 1999,Hyposaline | -0.67 | -65.02 | < 0.001 |
|  | 26 | 1999,Hypersaline - 2014,Hyposaline | -0.68 | -65.79 | < 0.001 |
|  | 27 | 1999,Hypersaline - 1986,Mesosaline | -0.51 | -50.05 | < 0.001 |
|  | 28 | 1999,Hypersaline - 1994,Mesosaline | -0.52 | -51.06 | < 0.001 |
|  | 29 | 1999,Hypersaline - 1999,Mesosaline | -0.50 | -48.70 | < 0.001 |
|  | 30 | 1999,Hypersaline - 2014,Mesosaline | -0.75 | -72.65 | < 0.001 |
|  | 31 | 2014,Hypersaline - 1986,Hyposaline | -0.36 | -35.16 | < 0.001 |
|  | 32 | 2014,Hypersaline - 1994,Hyposaline | -0.31 | -29.68 | < 0.001 |
|  | 33 | 2014,Hypersaline - 1999,Hyposaline | -0.32 | -31.42 | < 0.001 |
|  | 34 | 2014,Hypersaline - 2014,Hyposaline | -0.33 | -32.19 | < 0.001 |
|  | 35 | 2014,Hypersaline - 1986,Mesosaline | -0.17 | -16.45 | < 0.001 |
|  | 36 | 2014,Hypersaline - 1994,Mesosaline | -0.18 | -17.46 | < 0.001 |
|  | 37 | 2014,Hypersaline - 1999,Mesosaline | -0.16 | -15.10 | < 0.001 |
|  | 38 | 2014,Hypersaline - 2014,Mesosaline | -0.40 | -39.05 | < 0.001 |
|  | 39 | 1986,Hyposaline - 1994,Hyposaline | 0.06 | 5.48 | < 0.001 |
|  | 40 | 1986,Hyposaline - 1999,Hyposaline | 0.04 | 3.74 | < 0.05 |
|  | 41 | 1986,Hyposaline - 2014,Hyposaline | 0.03 | 2.97 | 0.117 |
|  | 42 | 1986,Hyposaline - 1986,Mesosaline | 0.19 | 18.71 | < 0.001 |
|  | 43 | 1986,Hyposaline - 1994,Mesosaline | 0.18 | 17.70 | < 0.001 |
|  | 44 | 1986,Hyposaline - 1999,Mesosaline | 0.21 | 20.06 | < 0.001 |
|  | 45 | 1986,Hyposaline - 2014,Mesosaline | -0.04 | -3.89 | < 0.01 |
|  | 46 | 1994,Hyposaline - 1999,Hyposaline | -0.02 | -1.74 | 0.850 |
|  | 47 | 1994,Hyposaline - 2014,Hyposaline | -0.03 | -2.50 | 0.337 |
|  | 48 | 1994,Hyposaline - 1986,Mesosaline | 0.14 | 13.23 | < 0.001 |
|  | 49 | 1994,Hyposaline - 1994,Mesosaline | 0.13 | 12.22 | < 0.001 |
|  | 50 | 1994,Hyposaline - 1999,Mesosaline | 0.15 | 14.59 | < 0.001 |
|  | 51 | 1994,Hyposaline - 2014,Mesosaline | -0.10 | -9.37 | < 0.001 |
|  | 52 | 1999,Hyposaline - 2014,Hyposaline | -0.01 | -0.77 | 0.95 |
|  | 53 | 1999,Hyposaline - 1986,Mesosaline | 0.15 | 14.97 | < 0.001 |
|  | 54 | 1999,Hyposaline - 1994,Mesosaline | 0.14 | 13.96 | < 0.001 |
|  | 55 | 1999,Hyposaline - 1999,Mesosaline | 0.17 | 16.32 | < 0.001 |
|  | 56 | 1999,Hyposaline - 2014,Mesosaline | -0.08 | -7.63 | < 0.001 |
|  | 57 | 2014,Hyposaline - 1986,Mesosaline | 0.16 | 15.74 | < 0.001 |
|  | 58 | 2014,Hyposaline - 1994,Mesosaline | 0.15 | 14.72 | < 0.001 |
|  | 59 | 2014,Hyposaline - 1999,Mesosaline | 0.18 | 17.09 | < 0.001 |
|  | 60 | 2014,Hyposaline - 2014,Mesosaline | -0.07 | -6.87 | < 0.001 |
|  | 61 | 1986,Mesosaline - 1994,Mesosaline | -0.01 | -1.01 | 0.955 |
|  | 62 | 1986,Mesosaline - 1999,Mesosaline | 0.01 | 1.35 | 0.972 |
|  | 63 | 1986,Mesosaline - 2014,Mesosaline | -0.23 | -22.60 | < 0.001 |
|  | 64 | 1994,Mesosaline - 1999,Mesosaline | 0.02 | 2.37 | 0.430 |
|  | 65 | 1994,Mesosaline - 2014,Mesosaline | -0.22 | -21.59 | < 0.001 |
|  | 66 | 1999,Mesosaline - 2014,Mesosaline | -0.25 | -23.96 | < 0.001 |
| ${}^{0}\overline{\rho}$ | 1 | 1986,Hypersaline - 1994,Hypersaline | -0.01 | -4.28 | < 0.01 |
|  | 2 | 1986,Hypersaline - 1999,Hypersaline | 0.00 | -2.51 | 0.335 |
|  | 3 | 1986,Hypersaline - 2014,Hypersaline | -0.01 | -6.61 | < 0.001 |
|  | 4 | 1986,Hypersaline - 1986,Hyposaline | 0.09 | 47.65 | < 0.001 |
|  | 5 | 1986,Hypersaline - 1994,Hyposaline | 0.07 | 38.77 | < 0.001 |
|  | 6 | 1986,Hypersaline - 1999,Hyposaline | 0.03 | 17.95 | < 0.001 |
|  | 7 | 1986,Hypersaline - 2014,Hyposaline | 0.04 | 22.78 | < 0.001 |
|  | 8 | 1986,Hypersaline - 1986,Mesosaline | -0.03 | -14.58 | < 0.001 |
|  | 9 | 1986,Hypersaline - 1994,Mesosaline | -0.03 | -14.95 | < 0.001 |
|  | 10 | 1986,Hypersaline - 1999,Mesosaline | -0.03 | -16.47 | < 0.001 |
|  | 11 | 1986,Hypersaline - 2014,Mesosaline | -0.02 | -11.27 | < 0.001 |
|  | 12 | 1994,Hypersaline - 1999,Hypersaline | 0.00 | 1.77 | 0.932 |
|  | 13 | 1994,Hypersaline - 2014,Hypersaline | 0.00 | -2.33 | 0.455 |
|  | 14 | 1994,Hypersaline - 1986,Hyposaline | 0.10 | 51.94 | < 0.001 |
|  | 15 | 1994,Hypersaline - 1994,Hyposaline | 0.08 | 43.06 | < 0.001 |
|  | 16 | 1994,Hypersaline - 1999,Hyposaline | 0.04 | 22.23 | < 0.001 |
|  | 17 | 1994,Hypersaline - 2014,Hyposaline | 0.05 | 27.06 | < 0.001 |
|  | 18 | 1994,Hypersaline - 1986,Mesosaline | -0.02 | -10.30 | < 0.001 |
|  | 19 | 1994,Hypersaline - 1994,Mesosaline | -0.02 | -10.67 | < 0.001 |
|  | 20 | 1994,Hypersaline - 1999,Mesosaline | -0.02 | -12.19 | < 0.001 |
|  | 21 | 1994,Hypersaline - 2014,Mesosaline | -0.01 | -6.99 | < 0.001 |
|  | 22 | 1999,Hypersaline - 2014,Hypersaline | -0.01 | -4.11 | < 0.01 |
|  | 23 | 1999,Hypersaline - 1986,Hyposaline | 0.09 | 50.16 | < 0.001 |
|  | 24 | 1999,Hypersaline - 1994,Hyposaline | 0.08 | 41.28 | < 0.001 |
|  | 25 | 1999,Hypersaline - 1999,Hyposaline | 0.04 | 20.45 | < 0.001 |
|  | 26 | 1999,Hypersaline - 2014,Hyposaline | 0.05 | 25.29 | < 0.001 |
|  | 27 | 1999,Hypersaline - 1986,Mesosaline | -0.02 | -12.08 | < 0.001 |
|  | 28 | 1999,Hypersaline - 1994,Mesosaline | -0.02 | -12.44 | < 0.001 |
|  | 29 | 1999,Hypersaline - 1999,Mesosaline | -0.03 | -13.96 | < 0.001 |
|  | 30 | 1999,Hypersaline - 2014,Mesosaline | -0.02 | -8.77 | < 0.001 |
|  | 31 | 2014,Hypersaline - 1986,Hyposaline | 0.10 | 54.27 | < 0.001 |
|  | 32 | 2014,Hypersaline - 1994,Hyposaline | 0.08 | 45.39 | < 0.001 |
|  | 33 | 2014,Hypersaline - 1999,Hyposaline | 0.05 | 24.56 | < 0.001 |
|  | 34 | 2014,Hypersaline - 2014,Hyposaline | 0.05 | 29.39 | < 0.001 |
|  | 35 | 2014,Hypersaline - 1986,Mesosaline | -0.01 | -7.97 | < 0.001 |
|  | 36 | 2014,Hypersaline - 1994,Mesosaline | -0.02 | -8.33 | < 0.001 |
|  | 37 | 2014,Hypersaline - 1999,Mesosaline | -0.02 | -9.86 | < 0.001 |
|  | 38 | 2014,Hypersaline - 2014,Mesosaline | -0.01 | -4.66 | < 0.001 |
|  | 39 | 1986,Hyposaline - 1994,Hyposaline | -0.02 | -8.88 | < 0.001 |
|  | 40 | 1986,Hyposaline - 1999,Hyposaline | -0.05 | -29.71 | < 0.001 |
|  | 41 | 1986,Hyposaline - 2014,Hyposaline | -0.05 | -24.88 | < 0.001 |
|  | 42 | 1986,Hyposaline - 1986,Mesosaline | -0.11 | -62.24 | < 0.001 |
|  | 43 | 1986,Hyposaline - 1994,Mesosaline | -0.11 | -62.60 | < 0.001 |
|  | 44 | 1986,Hyposaline - 1999,Mesosaline | -0.12 | -64.13 | < 0.001 |
|  | 45 | 1986,Hyposaline - 2014,Mesosaline | -0.11 | -58.93 | < 0.001 |
|  | 46 | 1994,Hyposaline - 1999,Hyposaline | -0.04 | -20.83 | < 0.001 |
|  | 47 | 1994,Hyposaline - 2014,Hyposaline | -0.03 | -16.00 | < 0.001 |
|  | 48 | 1994,Hyposaline - 1986,Mesosaline | -0.10 | -53.36 | < 0.001 |
|  | 49 | 1994,Hyposaline - 1994,Mesosaline | -0.10 | -53.72 | < 0.001 |
|  | 50 | 1994,Hyposaline - 1999,Mesosaline | -0.10 | -55.25 | < 0.001 |
|  | 51 | 1994,Hyposaline - 2014,Mesosaline | -0.09 | -50.05 | < 0.001 |
|  | 52 | 1999,Hyposaline - 2014,Hyposaline | 0.01 | 4.83 | < 0.001 |
|  | 53 | 1999,Hyposaline - 1986,Mesosaline | -0.06 | -32.53 | < 0.001 |
|  | 54 | 1999,Hyposaline - 1994,Mesosaline | -0.06 | -32.89 | < 0.001 |
|  | 55 | 1999,Hyposaline - 1999,Mesosaline | -0.06 | -34.42 | < 0.001 |
|  | 56 | 1999,Hyposaline - 2014,Mesosaline | -0.05 | -29.22 | < 0.001 |
|  | 57 | 2014,Hyposaline - 1986,Mesosaline | -0.07 | -37.36 | < 0.001 |
|  | 58 | 2014,Hyposaline - 1994,Mesosaline | -0.07 | -37.73 | < 0.001 |
|  | 59 | 2014,Hyposaline - 1999,Mesosaline | -0.07 | -39.25 | < 0.001 |
|  | 60 | 2014,Hyposaline - 2014,Mesosaline | -0.06 | -34.05 | < 0.001 |
|  | 61 | 1986,Mesosaline - 1994,Mesosaline | 0.00 | -0.36 | 0.985 |
|  | 62 | 1986,Mesosaline - 1999,Mesosaline | 0.00 | -1.89 | 0.767 |
|  | 63 | 1986,Mesosaline - 2014,Mesosaline | 0.01 | 3.31 | 0.554 |
|  | 64 | 1994,Mesosaline - 1999,Mesosaline | 0.00 | -1.52 | 0.934 |
|  | 65 | 1994,Mesosaline - 2014,Mesosaline | 0.01 | 3.67 | 0.655 |
|  | 66 | 1999,Mesosaline - 2014,Mesosaline | 0.01 | 5.20 | 0.556 |
| ${}^{1}\overline{\rho}$ | 1 | 1986,Hypersaline - 1994,Hypersaline | -0.05 | -13.16 | < 0.001 |
|  | 2 | 1986,Hypersaline - 1999,Hypersaline | -0.07 | -16.91 | < 0.001 |
|  | 3 | 1986,Hypersaline - 2014,Hypersaline | -0.08 | -19.32 | < 0.001 |
|  | 4 | 1986,Hypersaline - 1986,Hyposaline | 0.14 | 35.75 | < 0.001 |
|  | 5 | 1986,Hypersaline - 1994,Hyposaline | 0.13 | 31.29 | < 0.001 |
|  | 6 | 1986,Hypersaline - 1999,Hyposaline | 0.08 | 20.75 | < 0.001 |
|  | 7 | 1986,Hypersaline - 2014,Hyposaline | 0.09 | 21.63 | < 0.001 |
|  | 8 | 1986,Hypersaline - 1986,Mesosaline | -0.08 | -19.02 | < 0.001 |
|  | 9 | 1986,Hypersaline - 1994,Mesosaline | -0.08 | -19.91 | < 0.001 |
|  | 10 | 1986,Hypersaline - 1999,Mesosaline | -0.08 | -20.47 | < 0.001 |
|  | 11 | 1986,Hypersaline - 2014,Mesosaline | -0.07 | -16.93 | < 0.001 |
|  | 12 | 1994,Hypersaline - 1999,Hypersaline | -0.02 | -3.75 | < 0.01 |
|  | 13 | 1994,Hypersaline - 2014,Hypersaline | -0.02 | -6.17 | < 0.001 |
|  | 14 | 1994,Hypersaline - 1986,Hyposaline | 0.20 | 48.91 | < 0.001 |
|  | 15 | 1994,Hypersaline - 1994,Hyposaline | 0.18 | 44.45 | < 0.001 |
|  | 16 | 1994,Hypersaline - 1999,Hyposaline | 0.14 | 33.91 | < 0.001 |
|  | 17 | 1994,Hypersaline - 2014,Hyposaline | 0.14 | 34.78 | < 0.001 |
|  | 18 | 1994,Hypersaline - 1986,Mesosaline | -0.02 | -5.86 | < 0.001 |
|  | 19 | 1994,Hypersaline - 1994,Mesosaline | -0.03 | -6.76 | < 0.001 |
|  | 20 | 1994,Hypersaline - 1999,Mesosaline | -0.03 | -7.31 | < 0.001 |
|  | 21 | 1994,Hypersaline - 2014,Mesosaline | -0.02 | -3.77 | < 0.01 |
|  | 22 | 1999,Hypersaline - 2014,Hypersaline | -0.01 | -2.41 | 0.397 |
|  | 23 | 1999,Hypersaline - 1986,Hyposaline | 0.21 | 52.66 | < 0.001 |
|  | 24 | 1999,Hypersaline - 1994,Hyposaline | 0.19 | 48.20 | < 0.001 |
|  | 25 | 1999,Hypersaline - 1999,Hyposaline | 0.15 | 37.66 | < 0.001 |
|  | 26 | 1999,Hypersaline - 2014,Hyposaline | 0.15 | 38.54 | < 0.001 |
|  | 27 | 1999,Hypersaline - 1986,Mesosaline | -0.01 | -2.11 | 0.615 |
|  | 28 | 1999,Hypersaline - 1994,Mesosaline | -0.01 | -3.00 | 0.108 |
|  | 29 | 1999,Hypersaline - 1999,Mesosaline | -0.01 | -3.56 | < 0.05 |
|  | 30 | 1999,Hypersaline - 2014,Mesosaline | 0.00 | -0.02 | 0.986 |
|  | 31 | 2014,Hypersaline - 1986,Hyposaline | 0.22 | 55.07 | < 0.001 |
|  | 32 | 2014,Hypersaline - 1994,Hyposaline | 0.20 | 50.62 | < 0.001 |
|  | 33 | 2014,Hypersaline - 1999,Hyposaline | 0.16 | 40.08 | < 0.001 |
|  | 34 | 2014,Hypersaline - 2014,Hyposaline | 0.16 | 40.95 | < 0.001 |
|  | 35 | 2014,Hypersaline - 1986,Mesosaline | 0.00 | 0.30 | 0.975 |
|  | 36 | 2014,Hypersaline - 1994,Mesosaline | 0.00 | -0.59 | 0.955 |
|  | 37 | 2014,Hypersaline - 1999,Mesosaline | 0.00 | -1.14 | 0.948 |
|  | 38 | 2014,Hypersaline - 2014,Mesosaline | 0.01 | 2.40 | 0.409 |
|  | 39 | 1986,Hyposaline - 1994,Hyposaline | -0.02 | -4.46 | < 0.001 |
|  | 40 | 1986,Hyposaline - 1999,Hyposaline | -0.06 | -15.00 | < 0.001 |
|  | 41 | 1986,Hyposaline - 2014,Hyposaline | -0.06 | -14.12 | < 0.001 |
|  | 42 | 1986,Hyposaline - 1986,Mesosaline | -0.22 | -54.77 | < 0.001 |
|  | 43 | 1986,Hyposaline - 1994,Mesosaline | -0.22 | -55.66 | < 0.001 |
|  | 44 | 1986,Hyposaline - 1999,Mesosaline | -0.23 | -56.21 | < 0.001 |
|  | 45 | 1986,Hyposaline - 2014,Mesosaline | -0.21 | -52.68 | < 0.001 |
|  | 46 | 1994,Hyposaline - 1999,Hyposaline | -0.04 | -10.54 | < 0.001 |
|  | 47 | 1994,Hyposaline - 2014,Hyposaline | -0.04 | -9.67 | < 0.001 |
|  | 48 | 1994,Hyposaline - 1986,Mesosaline | -0.20 | -50.31 | < 0.001 |
|  | 49 | 1994,Hyposaline - 1994,Mesosaline | -0.21 | -51.21 | < 0.001 |
|  | 50 | 1994,Hyposaline - 1999,Mesosaline | -0.21 | -51.76 | < 0.001 |
|  | 51 | 1994,Hyposaline - 2014,Mesosaline | -0.19 | -48.22 | < 0.001 |
|  | 52 | 1999,Hyposaline - 2014,Hyposaline | 0.00 | 0.87 | 0.955 |
|  | 53 | 1999,Hyposaline - 1986,Mesosaline | -0.16 | -39.77 | < 0.001 |
|  | 54 | 1999,Hyposaline - 1994,Mesosaline | -0.16 | -40.67 | < 0.001 |
|  | 55 | 1999,Hyposaline - 1999,Mesosaline | -0.17 | -41.22 | < 0.001 |
|  | 56 | 1999,Hyposaline - 2014,Mesosaline | -0.15 | -37.68 | < 0.001 |
|  | 57 | 2014,Hyposaline - 1986,Mesosaline | -0.16 | -40.65 | < 0.001 |
|  | 58 | 2014,Hyposaline - 1994,Mesosaline | -0.17 | -41.54 | < 0.001 |
|  | 59 | 2014,Hyposaline - 1999,Mesosaline | -0.17 | -42.09 | < 0.001 |
|  | 60 | 2014,Hyposaline - 2014,Mesosaline | -0.15 | -38.55 | < 0.001 |
|  | 61 | 1986,Mesosaline - 1994,Mesosaline | 0.00 | -0.89 | 0.966 |
|  | 62 | 1986,Mesosaline - 1999,Mesosaline | -0.01 | -1.45 | 0.954 |
|  | 63 | 1986,Mesosaline - 2014,Mesosaline | 0.01 | 2.09 | 0.628 |
|  | 64 | 1994,Mesosaline - 1999,Mesosaline | 0.00 | -0.55 | 0.952 |
|  | 65 | 1994,Mesosaline - 2014,Mesosaline | 0.01 | 2.99 | 0.113 |
|  | 66 | 1999,Mesosaline - 2014,Mesosaline | 0.01 | 3.54 | < 0.05 |
| ${}^{2}\overline{\rho}$ | 1 | 1986,Hypersaline - 1994,Hypersaline | -0.03 | -6.13 | < 0.001 |
|  | 2 | 1986,Hypersaline - 1999,Hypersaline | -0.07 | -13.01 | < 0.001 |
|  | 3 | 1986,Hypersaline - 2014,Hypersaline | -0.07 | -14.19 | < 0.001 |
|  | 4 | 1986,Hypersaline - 1986,Hyposaline | 0.17 | 33.48 | < 0.001 |
|  | 5 | 1986,Hypersaline - 1994,Hyposaline | 0.16 | 30.40 | < 0.001 |
|  | 6 | 1986,Hypersaline - 1999,Hyposaline | 0.12 | 22.59 | < 0.001 |
|  | 7 | 1986,Hypersaline - 2014,Hyposaline | 0.13 | 24.33 | < 0.001 |
|  | 8 | 1986,Hypersaline - 1986,Mesosaline | -0.06 | -12.39 | < 0.001 |
|  | 9 | 1986,Hypersaline - 1994,Mesosaline | -0.08 | -14.59 | < 0.001 |
|  | 10 | 1986,Hypersaline - 1999,Mesosaline | -0.08 | -15.93 | < 0.001 |
|  | 11 | 1986,Hypersaline - 2014,Mesosaline | -0.06 | -12.47 | < 0.001 |
|  | 12 | 1994,Hypersaline - 1999,Hypersaline | -0.04 | -6.89 | < 0.001 |
|  | 13 | 1994,Hypersaline - 2014,Hypersaline | -0.04 | -8.06 | < 0.001 |
|  | 14 | 1994,Hypersaline - 1986,Hyposaline | 0.20 | 39.61 | < 0.001 |
|  | 15 | 1994,Hypersaline - 1994,Hyposaline | 0.19 | 36.53 | < 0.001 |
|  | 16 | 1994,Hypersaline - 1999,Hyposaline | 0.15 | 28.72 | < 0.001 |
|  | 17 | 1994,Hypersaline - 2014,Hyposaline | 0.16 | 30.46 | < 0.001 |
|  | 18 | 1994,Hypersaline - 1986,Mesosaline | -0.03 | -6.26 | < 0.001 |
|  | 19 | 1994,Hypersaline - 1994,Mesosaline | -0.04 | -8.46 | < 0.001 |
|  | 20 | 1994,Hypersaline - 1999,Mesosaline | -0.05 | -9.80 | < 0.001 |
|  | 21 | 1994,Hypersaline - 2014,Mesosaline | -0.03 | -6.34 | < 0.001 |
|  | 22 | 1999,Hypersaline - 2014,Hypersaline | -0.01 | -1.17 | 0.976 |
|  | 23 | 1999,Hypersaline - 1986,Hyposaline | 0.24 | 46.49 | < 0.001 |
|  | 24 | 1999,Hypersaline - 1994,Hyposaline | 0.22 | 43.42 | < 0.001 |
|  | 25 | 1999,Hypersaline - 1999,Hyposaline | 0.18 | 35.61 | < 0.001 |
|  | 26 | 1999,Hypersaline - 2014,Hyposaline | 0.19 | 37.35 | < 0.001 |
|  | 27 | 1999,Hypersaline - 1986,Mesosaline | 0.00 | 0.63 | 0.964 |
|  | 28 | 1999,Hypersaline - 1994,Mesosaline | -0.01 | -1.58 | 0.918 |
|  | 29 | 1999,Hypersaline - 1999,Mesosaline | -0.01 | -2.91 | 0.137 |
|  | 30 | 1999,Hypersaline - 2014,Mesosaline | 0.00 | 0.55 | 0.968 |
|  | 31 | 2014,Hypersaline - 1986,Hyposaline | 0.25 | 47.67 | < 0.001 |
|  | 32 | 2014,Hypersaline - 1994,Hyposaline | 0.23 | 44.59 | < 0.001 |
|  | 33 | 2014,Hypersaline - 1999,Hyposaline | 0.19 | 36.78 | < 0.001 |
|  | 34 | 2014,Hypersaline - 2014,Hyposaline | 0.20 | 38.52 | < 0.001 |
|  | 35 | 2014,Hypersaline - 1986,Mesosaline | 0.01 | 1.80 | 0.818 |
|  | 36 | 2014,Hypersaline - 1994,Mesosaline | 0.00 | -0.40 | 0.988 |
|  | 37 | 2014,Hypersaline - 1999,Mesosaline | -0.01 | -1.74 | 0.850 |
|  | 38 | 2014,Hypersaline - 2014,Mesosaline | 0.01 | 1.72 | 0.859 |
|  | 39 | 1986,Hyposaline - 1994,Hyposaline | -0.02 | -3.08 | 0.087 |
|  | 40 | 1986,Hyposaline - 1999,Hyposaline | -0.06 | -10.89 | < 0.001 |
|  | 41 | 1986,Hyposaline - 2014,Hyposaline | -0.05 | -9.15 | < 0.001 |
|  | 42 | 1986,Hyposaline - 1986,Mesosaline | -0.24 | -45.87 | < 0.001 |
|  | 43 | 1986,Hyposaline - 1994,Mesosaline | -0.25 | -48.07 | < 0.001 |
|  | 44 | 1986,Hyposaline - 1999,Mesosaline | -0.25 | -49.41 | < 0.001 |
|  | 45 | 1986,Hyposaline - 2014,Mesosaline | -0.24 | -45.95 | < 0.001 |
|  | 46 | 1994,Hyposaline - 1999,Hyposaline | -0.04 | -7.81 | < 0.001 |
|  | 47 | 1994,Hyposaline - 2014,Hyposaline | -0.03 | -6.07 | < 0.001 |
|  | 48 | 1994,Hyposaline - 1986,Mesosaline | -0.22 | -42.79 | < 0.001 |
|  | 49 | 1994,Hyposaline - 1994,Mesosaline | -0.23 | -44.99 | < 0.001 |
|  | 50 | 1994,Hyposaline - 1999,Mesosaline | -0.24 | -46.33 | < 0.001 |
|  | 51 | 1994,Hyposaline - 2014,Mesosaline | -0.22 | -42.87 | < 0.001 |
|  | 52 | 1999,Hyposaline - 2014,Hyposaline | 0.01 | 1.74 | 0.850 |
|  | 53 | 1999,Hyposaline - 1986,Mesosaline | -0.18 | -34.98 | < 0.001 |
|  | 54 | 1999,Hyposaline - 1994,Mesosaline | -0.19 | -37.18 | < 0.001 |
|  | 55 | 1999,Hyposaline - 1999,Mesosaline | -0.20 | -38.52 | < 0.001 |
|  | 56 | 1999,Hyposaline - 2014,Mesosaline | -0.18 | -35.06 | < 0.001 |
|  | 57 | 2014,Hyposaline - 1986,Mesosaline | -0.19 | -36.72 | < 0.001 |
|  | 58 | 2014,Hyposaline - 1994,Mesosaline | -0.20 | -38.92 | < 0.001 |
|  | 59 | 2014,Hyposaline - 1999,Mesosaline | -0.21 | -40.26 | < 0.001 |
|  | 60 | 2014,Hyposaline - 2014,Mesosaline | -0.19 | -36.80 | < 0.001 |
|  | 61 | 1986,Mesosaline - 1994,Mesosaline | -0.01 | -2.20 | 0.547 |
|  | 62 | 1986,Mesosaline - 1999,Mesosaline | -0.02 | -3.54 | < 0.05 |
|  | 63 | 1986,Mesosaline - 2014,Mesosaline | 0.00 | -0.08 | 0.975 |
|  | 64 | 1994,Mesosaline - 1999,Mesosaline | -0.01 | -1.34 | 0.974 |
|  | 65 | 1994,Mesosaline - 2014,Mesosaline | 0.01 | 2.12 | 0.606 |
|  | 66 | 1999,Mesosaline - 2014,Mesosaline | 0.02 | 3.46 | < 0.05 |
| ${}^{0}\gamma$ | 1 | 1986,Hypersaline - 1994,Hypersaline | 0.52 | 0.98 | 0.975 |
|  | 2 | 1986,Hypersaline - 1999,Hypersaline | 0.19 | 0.35 | 0.966 |
|  | 3 | 1986,Hypersaline - 2014,Hypersaline | 0.70 | 1.32 | 0.976 |
|  | 4 | 1986,Hypersaline - 1986,Hyposaline | -14.90 | -28.25 | < 0.001 |
|  | 5 | 1986,Hypersaline - 1994,Hyposaline | -7.97 | -15.11 | < 0.001 |
|  | 6 | 1986,Hypersaline - 1999,Hyposaline | -7.16 | -13.56 | < 0.001 |
|  | 7 | 1986,Hypersaline - 2014,Hyposaline | -7.97 | -15.11 | < 0.001 |
|  | 8 | 1986,Hypersaline - 1986,Mesosaline | 0.66 | 1.25 | 0.985 |
|  | 9 | 1986,Hypersaline - 1994,Mesosaline | 1.75 | 3.31 | < 0.05 |
|  | 10 | 1986,Hypersaline - 1999,Mesosaline | 3.24 | 6.13 | < 0.001 |
|  | 11 | 1986,Hypersaline - 2014,Mesosaline | -0.04 | -0.08 | 0.988 |
|  | 12 | 1994,Hypersaline - 1999,Hypersaline | -0.33 | -0.63 | 0.975 |
|  | 13 | 1994,Hypersaline - 2014,Hypersaline | 0.18 | 0.34 | 0.968 |
|  | 14 | 1994,Hypersaline - 1986,Hyposaline | -15.42 | -29.23 | < 0.001 |
|  | 15 | 1994,Hypersaline - 1994,Hyposaline | -8.49 | -16.09 | < 0.001 |
|  | 16 | 1994,Hypersaline - 1999,Hyposaline | -7.67 | -14.55 | < 0.001 |
|  | 17 | 1994,Hypersaline - 2014,Hyposaline | -8.49 | -16.10 | < 0.001 |
|  | 18 | 1994,Hypersaline - 1986,Mesosaline | 0.14 | 0.27 | < 0.001 |
|  | 19 | 1994,Hypersaline - 1994,Mesosaline | 1.23 | 2.33 | 0.456 |
|  | 20 | 1994,Hypersaline - 1999,Mesosaline | 2.72 | 5.15 | < 0.001 |
|  | 21 | 1994,Hypersaline - 2014,Mesosaline | -0.56 | -1.06 | 0.972 |
|  | 22 | 1999,Hypersaline - 2014,Hypersaline | 0.51 | 0.97 | 0.974 |
|  | 23 | 1999,Hypersaline - 1986,Hyposaline | -15.09 | -28.60 | < 0.001 |
|  | 24 | 1999,Hypersaline - 1994,Hyposaline | -8.16 | -15.46 | < 0.001 |
|  | 25 | 1999,Hypersaline - 1999,Hyposaline | -7.34 | -13.92 | < 0.001 |
|  | 26 | 1999,Hypersaline - 2014,Hyposaline | -8.16 | -15.47 | < 0.001 |
|  | 27 | 1999,Hypersaline - 1986,Mesosaline | 0.48 | 0.90 | 0.955 |
|  | 28 | 1999,Hypersaline - 1994,Mesosaline | 1.56 | 2.96 | 0.121 |
|  | 29 | 1999,Hypersaline - 1999,Mesosaline | 3.05 | 5.78 | < 0.001 |
|  | 30 | 1999,Hypersaline - 2014,Mesosaline | -0.23 | -0.43 | 0.977 |
|  | 31 | 2014,Hypersaline - 1986,Hyposaline | -15.60 | -29.57 | < 0.001 |
|  | 32 | 2014,Hypersaline - 1994,Hyposaline | -8.67 | -16.43 | < 0.001 |
|  | 33 | 2014,Hypersaline - 1999,Hyposaline | -7.85 | -14.89 | < 0.001 |
|  | 34 | 2014,Hypersaline - 2014,Hyposaline | -8.67 | -16.44 | < 0.001 |
|  | 35 | 2014,Hypersaline - 1986,Mesosaline | -0.04 | -0.07 | 0.988 |
|  | 36 | 2014,Hypersaline - 1994,Mesosaline | 1.05 | 1.99 | 0.702 |
|  | 37 | 2014,Hypersaline - 1999,Mesosaline | 2.54 | 4.81 | < 0.001 |
|  | 38 | 2014,Hypersaline - 2014,Mesosaline | -0.74 | -1.40 | 0.963 |
|  | 39 | 1986,Hyposaline - 1994,Hyposaline | 6.93 | 13.14 | < 0.001 |
|  | 40 | 1986,Hyposaline - 1999,Hyposaline | 7.75 | 14.69 | < 0.001 |
|  | 41 | 1986,Hyposaline - 2014,Hyposaline | 6.93 | 13.14 | < 0.001 |
|  | 42 | 1986,Hyposaline - 1986,Mesosaline | 15.57 | 29.50 | < 0.001 |
|  | 43 | 1986,Hyposaline - 1994,Mesosaline | 16.65 | 31.56 | < 0.001 |
|  | 44 | 1986,Hyposaline - 1999,Mesosaline | 18.14 | 34.39 | < 0.001 |
|  | 45 | 1986,Hyposaline - 2014,Mesosaline | 14.86 | 28.17 | < 0.001 |
|  | 46 | 1994,Hyposaline - 1999,Hyposaline | 0.82 | 1.54 | 0.928 |
|  | 47 | 1994,Hyposaline - 2014,Hyposaline | 0.00 | -0.01 | 0.985 |
|  | 48 | 1994,Hyposaline - 1986,Mesosaline | 8.63 | 16.36 | < 0.001 |
|  | 49 | 1994,Hyposaline - 1994,Mesosaline | 9.72 | 18.42 | < 0.001 |
|  | 50 | 1994,Hyposaline - 1999,Mesosaline | 11.21 | 21.24 | < 0.001 |
|  | 51 | 1994,Hyposaline - 2014,Mesosaline | 7.93 | 15.03 | < 0.001 |
|  | 52 | 1999,Hyposaline - 2014,Hyposaline | -0.82 | -1.55 | 0.926 |
|  | 53 | 1999,Hyposaline - 1986,Mesosaline | 7.82 | 14.82 | < 0.001 |
|  | 54 | 1999,Hyposaline - 1994,Mesosaline | 8.90 | 16.87 | < 0.001 |
|  | 55 | 1999,Hyposaline - 1999,Mesosaline | 10.39 | 19.70 | < 0.001 |
|  | 56 | 1999,Hyposaline - 2014,Mesosaline | 7.11 | 13.48 | < 0.001 |
|  | 57 | 2014,Hyposaline - 1986,Mesosaline | 8.64 | 16.37 | < 0.001 |
|  | 58 | 2014,Hyposaline - 1994,Mesosaline | 9.72 | 18.43 | < 0.001 |
|  | 59 | 2014,Hyposaline - 1999,Mesosaline | 11.21 | 21.25 | < 0.001 |
|  | 60 | 2014,Hyposaline - 2014,Mesosaline | 7.93 | 15.04 | < 0.001 |
|  | 61 | 1986,Mesosaline - 1994,Mesosaline | 1.09 | 2.06 | 0.653 |
|  | 62 | 1986,Mesosaline - 1999,Mesosaline | 2.58 | 4.88 | < 0.001 |
|  | 63 | 1986,Mesosaline - 2014,Mesosaline | -0.70 | -1.33 | 0.975 |
|  | 64 | 1994,Mesosaline - 1999,Mesosaline | 1.49 | 2.82 | 0.170 |
|  | 65 | 1994,Mesosaline - 2014,Mesosaline | -1.79 | -3.39 | < 0.05 |
|  | 66 | 1999,Mesosaline - 2014,Mesosaline | -3.28 | -6.21 | < 0.001 |
| ${}^{1}\gamma$ | 1 | 1986,Hypersaline - 1994,Hypersaline | 1.83 | 12.80 | < 0.001 |
|  | 2 | 1986,Hypersaline - 1999,Hypersaline | 1.93 | 13.53 | < 0.001 |
|  | 3 | 1986,Hypersaline - 2014,Hypersaline | 1.37 | 9.61 | < 0.001 |
|  | 4 | 1986,Hypersaline - 1986,Hyposaline | -3.38 | -23.64 | < 0.001 |
|  | 5 | 1986,Hypersaline - 1994,Hyposaline | -2.66 | -18.62 | < 0.001 |
|  | 6 | 1986,Hypersaline - 1999,Hyposaline | -1.34 | -9.35 | < 0.001 |
|  | 7 | 1986,Hypersaline - 2014,Hyposaline | -0.36 | -2.53 | 0.318 |
|  | 8 | 1986,Hypersaline - 1986,Mesosaline | 1.46 | 10.23 | < 0.001 |
|  | 9 | 1986,Hypersaline - 1994,Mesosaline | 1.48 | 10.37 | < 0.001 |
|  | 10 | 1986,Hypersaline - 1999,Mesosaline | 1.53 | 10.70 | < 0.001 |
|  | 11 | 1986,Hypersaline - 2014,Mesosaline | 1.06 | 7.43 | < 0.001 |
|  | 12 | 1994,Hypersaline - 1999,Hypersaline | 0.10 | 0.73 | 0.976 |
|  | 13 | 1994,Hypersaline - 2014,Hypersaline | -0.46 | -3.19 | 0.064 |
|  | 14 | 1994,Hypersaline - 1986,Hyposaline | -5.21 | -36.44 | < 0.001 |
|  | 15 | 1994,Hypersaline - 1994,Hyposaline | -4.49 | -31.41 | < 0.001 |
|  | 16 | 1994,Hypersaline - 1999,Hyposaline | -3.17 | -22.14 | < 0.001 |
|  | 17 | 1994,Hypersaline - 2014,Hyposaline | -2.19 | -15.33 | < 0.001 |
|  | 18 | 1994,Hypersaline - 1986,Mesosaline | -0.37 | -2.57 | 0.298 |
|  | 19 | 1994,Hypersaline - 1994,Mesosaline | -0.35 | -2.43 | 0.386 |
|  | 20 | 1994,Hypersaline - 1999,Mesosaline | -0.30 | -2.10 | 0.622 |
|  | 21 | 1994,Hypersaline - 2014,Mesosaline | -0.77 | -5.37 | < 0.001 |
|  | 22 | 1999,Hypersaline - 2014,Hypersaline | -0.56 | -3.91 | < 0.01 |
|  | 23 | 1999,Hypersaline - 1986,Hyposaline | -5.32 | -37.17 | < 0.001 |
|  | 24 | 1999,Hypersaline - 1994,Hyposaline | -4.60 | -32.14 | < 0.001 |
|  | 25 | 1999,Hypersaline - 1999,Hyposaline | -3.27 | -22.87 | < 0.001 |
|  | 26 | 1999,Hypersaline - 2014,Hyposaline | -2.30 | -16.06 | < 0.001 |
|  | 27 | 1999,Hypersaline - 1986,Mesosaline | -0.47 | -3.29 | < 0.05 |
|  | 28 | 1999,Hypersaline - 1994,Mesosaline | -0.45 | -3.16 | 0.070 |
|  | 29 | 1999,Hypersaline - 1999,Mesosaline | -0.40 | -2.83 | 0.168 |
|  | 30 | 1999,Hypersaline - 2014,Mesosaline | -0.87 | -6.09 | < 0.001 |
|  | 31 | 2014,Hypersaline - 1986,Hyposaline | -4.76 | -33.26 | < 0.001 |
|  | 32 | 2014,Hypersaline - 1994,Hyposaline | -4.04 | -28.23 | < 0.001 |
|  | 33 | 2014,Hypersaline - 1999,Hyposaline | -2.71 | -18.96 | < 0.001 |
|  | 34 | 2014,Hypersaline - 2014,Hyposaline | -1.74 | -12.15 | < 0.001 |
|  | 35 | 2014,Hypersaline - 1986,Mesosaline | 0.09 | 0.62 | 0.977 |
|  | 36 | 2014,Hypersaline - 1994,Mesosaline | 0.11 | 0.76 | 0.984 |
|  | 37 | 2014,Hypersaline - 1999,Mesosaline | 0.16 | 1.09 | 0.988 |
|  | 38 | 2014,Hypersaline - 2014,Mesosaline | -0.31 | -2.18 | 0.564 |
|  | 39 | 1986,Hyposaline - 1994,Hyposaline | 0.72 | 5.03 | < 0.001 |
|  | 40 | 1986,Hyposaline - 1999,Hyposaline | 2.04 | 14.30 | < 0.001 |
|  | 41 | 1986,Hyposaline - 2014,Hyposaline | 3.02 | 21.11 | < 0.001 |
|  | 42 | 1986,Hyposaline - 1986,Mesosaline | 4.84 | 33.87 | < 0.001 |
|  | 43 | 1986,Hyposaline - 1994,Mesosaline | 4.86 | 34.01 | < 0.001 |
|  | 44 | 1986,Hyposaline - 1999,Mesosaline | 4.91 | 34.34 | < 0.001 |
|  | 45 | 1986,Hyposaline - 2014,Mesosaline | 4.44 | 31.08 | < 0.001 |
|  | 46 | 1994,Hyposaline - 1999,Hyposaline | 1.33 | 9.27 | < 0.001 |
|  | 47 | 1994,Hyposaline - 2014,Hyposaline | 2.30 | 16.08 | < 0.001 |
|  | 48 | 1994,Hyposaline - 1986,Mesosaline | 4.13 | 28.85 | < 0.001 |
|  | 49 | 1994,Hyposaline - 1994,Mesosaline | 4.15 | 28.99 | < 0.001 |
|  | 50 | 1994,Hyposaline - 1999,Mesosaline | 4.19 | 29.31 | < 0.001 |
|  | 51 | 1994,Hyposaline - 2014,Mesosaline | 3.73 | 26.05 | < 0.001 |
|  | 52 | 1999,Hyposaline - 2014,Hyposaline | 0.97 | 6.81 | < 0.001 |
|  | 53 | 1999,Hyposaline - 1986,Mesosaline | 2.80 | 19.58 | < 0.001 |
|  | 54 | 1999,Hyposaline - 1994,Mesosaline | 2.82 | 19.72 | < 0.001 |
|  | 55 | 1999,Hyposaline - 1999,Mesosaline | 2.87 | 20.04 | < 0.001 |
|  | 56 | 1999,Hyposaline - 2014,Mesosaline | 2.40 | 16.78 | < 0.001 |
|  | 57 | 2014,Hyposaline - 1986,Mesosaline | 1.83 | 12.77 | < 0.001 |
|  | 58 | 2014,Hyposaline - 1994,Mesosaline | 1.85 | 12.91 | < 0.001 |
|  | 59 | 2014,Hyposaline - 1999,Mesosaline | 1.89 | 13.23 | < 0.001 |
|  | 60 | 2014,Hyposaline - 2014,Mesosaline | 1.43 | 9.97 | < 0.001 |
|  | 61 | 1986,Mesosaline - 1994,Mesosaline | 0.02 | 0.14 | 0.985 |
|  | 62 | 1986,Mesosaline - 1999,Mesosaline | 0.07 | 0.47 | 0.986 |
|  | 63 | 1986,Mesosaline - 2014,Mesosaline | -0.40 | -2.80 | 0.180 |
|  | 64 | 1994,Mesosaline - 1999,Mesosaline | 0.05 | 0.33 | 0.978 |
|  | 65 | 1994,Mesosaline - 2014,Mesosaline | -0.42 | -2.94 | 0.127 |
|  | 66 | 1999,Mesosaline - 2014,Mesosaline | -0.47 | -3.27 | 0.051 |
| ${}^{2}\gamma$ | 1 | 1986,Hypersaline - 1994,Hypersaline | 1.06 | 12.53 | < 0.001 |
|  | 2 | 1986,Hypersaline - 1999,Hypersaline | 1.10 | 13.02 | < 0.001 |
|  | 3 | 1986,Hypersaline - 2014,Hypersaline | 0.77 | 9.14 | < 0.001 |
|  | 4 | 1986,Hypersaline - 1986,Hyposaline | -2.21 | -26.09 | < 0.001 |
|  | 5 | 1986,Hypersaline - 1994,Hyposaline | -1.71 | -20.13 | < 0.001 |
|  | 6 | 1986,Hypersaline - 1999,Hyposaline | -1.07 | -12.60 | < 0.001 |
|  | 7 | 1986,Hypersaline - 2014,Hyposaline | -0.24 | -2.78 | 0.189 |
|  | 8 | 1986,Hypersaline - 1986,Mesosaline | 0.33 | 3.87 | < 0.01 |
|  | 9 | 1986,Hypersaline - 1994,Mesosaline | 0.33 | 3.93 | < 0.01 |
|  | 10 | 1986,Hypersaline - 1999,Mesosaline | 0.35 | 4.10 | < 0.01 |
|  | 11 | 1986,Hypersaline - 2014,Mesosaline | 0.12 | 1.40 | 0.964 |
|  | 12 | 1994,Hypersaline - 1999,Hypersaline | 0.04 | 0.49 | 0.985 |
|  | 13 | 1994,Hypersaline - 2014,Hypersaline | -0.29 | -3.39 | < 0.05 |
|  | 14 | 1994,Hypersaline - 1986,Hyposaline | -3.27 | -38.62 | < 0.001 |
|  | 15 | 1994,Hypersaline - 1994,Hyposaline | -2.77 | -32.66 | < 0.001 |
|  | 16 | 1994,Hypersaline - 1999,Hyposaline | -2.13 | -25.14 | < 0.001 |
|  | 17 | 1994,Hypersaline - 2014,Hyposaline | -1.30 | -15.31 | < 0.001 |
|  | 18 | 1994,Hypersaline - 1986,Mesosaline | -0.73 | -8.66 | < 0.001 |
|  | 19 | 1994,Hypersaline - 1994,Mesosaline | -0.73 | -8.60 | < 0.001 |
|  | 20 | 1994,Hypersaline - 1999,Mesosaline | -0.71 | -8.43 | < 0.001 |
|  | 21 | 1994,Hypersaline - 2014,Mesosaline | -0.94 | -11.13 | < 0.001 |
|  | 22 | 1999,Hypersaline - 2014,Hypersaline | -0.33 | -3.88 | < 0.01 |
|  | 23 | 1999,Hypersaline - 1986,Hyposaline | -3.31 | -39.11 | < 0.001 |
|  | 24 | 1999,Hypersaline - 1994,Hyposaline | -2.81 | -33.16 | < 0.001 |
|  | 25 | 1999,Hypersaline - 1999,Hyposaline | -2.17 | -25.63 | < 0.001 |
|  | 26 | 1999,Hypersaline - 2014,Hyposaline | -1.34 | -15.80 | < 0.001 |
|  | 27 | 1999,Hypersaline - 1986,Mesosaline | -0.78 | -9.15 | < 0.001 |
|  | 28 | 1999,Hypersaline - 1994,Mesosaline | -0.77 | -9.09 | < 0.001 |
|  | 29 | 1999,Hypersaline - 1999,Mesosaline | -0.76 | -8.92 | < 0.001 |
|  | 30 | 1999,Hypersaline - 2014,Mesosaline | -0.99 | -11.63 | < 0.001 |
|  | 31 | 2014,Hypersaline - 1986,Hyposaline | -2.99 | -35.23 | < 0.001 |
|  | 32 | 2014,Hypersaline - 1994,Hyposaline | -2.48 | -29.27 | < 0.001 |
|  | 33 | 2014,Hypersaline - 1999,Hyposaline | -1.84 | -21.75 | < 0.001 |
|  | 34 | 2014,Hypersaline - 2014,Hyposaline | -1.01 | -11.92 | < 0.001 |
|  | 35 | 2014,Hypersaline - 1986,Mesosaline | -0.45 | -5.27 | < 0.001 |
|  | 36 | 2014,Hypersaline - 1994,Mesosaline | -0.44 | -5.21 | < 0.001 |
|  | 37 | 2014,Hypersaline - 1999,Mesosaline | -0.43 | -5.04 | < 0.001 |
|  | 38 | 2014,Hypersaline - 2014,Mesosaline | -0.66 | -7.74 | < 0.001 |
|  | 39 | 1986,Hyposaline - 1994,Hyposaline | 0.50 | 5.95 | < 0.001 |
|  | 40 | 1986,Hyposaline - 1999,Hyposaline | 1.14 | 13.48 | < 0.001 |
|  | 41 | 1986,Hyposaline - 2014,Hyposaline | 1.98 | 23.31 | < 0.001 |
|  | 42 | 1986,Hyposaline - 1986,Mesosaline | 2.54 | 29.96 | < 0.001 |
|  | 43 | 1986,Hyposaline - 1994,Mesosaline | 2.54 | 30.02 | < 0.001 |
|  | 44 | 1986,Hyposaline - 1999,Mesosaline | 2.56 | 30.19 | < 0.001 |
|  | 45 | 1986,Hyposaline - 2014,Mesosaline | 2.33 | 27.48 | < 0.001 |
|  | 46 | 1994,Hyposaline - 1999,Hyposaline | 0.64 | 7.53 | < 0.001 |
|  | 47 | 1994,Hyposaline - 2014,Hyposaline | 1.47 | 17.35 | < 0.001 |
|  | 48 | 1994,Hyposaline - 1986,Mesosaline | 2.03 | 24.00 | < 0.001 |
|  | 49 | 1994,Hyposaline - 1994,Mesosaline | 2.04 | 24.06 | < 0.001 |
|  | 50 | 1994,Hyposaline - 1999,Mesosaline | 2.05 | 24.23 | < 0.001 |
|  | 51 | 1994,Hyposaline - 2014,Mesosaline | 1.82 | 21.53 | < 0.001 |
|  | 52 | 1999,Hyposaline - 2014,Hyposaline | 0.83 | 9.82 | < 0.001 |
|  | 53 | 1999,Hyposaline - 1986,Mesosaline | 1.40 | 16.47 | < 0.001 |
|  | 54 | 1999,Hyposaline - 1994,Mesosaline | 1.40 | 16.53 | < 0.001 |
|  | 55 | 1999,Hyposaline - 1999,Mesosaline | 1.42 | 16.71 | < 0.001 |
|  | 56 | 1999,Hyposaline - 2014,Mesosaline | 1.19 | 14.00 | < 0.001 |
|  | 57 | 2014,Hyposaline - 1986,Mesosaline | 0.56 | 6.65 | < 0.001 |
|  | 58 | 2014,Hyposaline - 1994,Mesosaline | 0.57 | 6.71 | < 0.001 |
|  | 59 | 2014,Hyposaline - 1999,Mesosaline | 0.58 | 6.88 | < 0.001 |
|  | 60 | 2014,Hyposaline - 2014,Mesosaline | 0.35 | 4.18 | < 0.01 |
|  | 61 | 1986,Mesosaline - 1994,Mesosaline | 0.01 | 0.06 | 0.988 |
|  | 62 | 1986,Mesosaline - 1999,Mesosaline | 0.02 | 0.23 | 0.985 |
|  | 63 | 1986,Mesosaline - 2014,Mesosaline | -0.21 | -2.47 | 0.358 |
|  | 64 | 1994,Mesosaline - 1999,Mesosaline | 0.01 | 0.17 | 0.989 |
|  | 65 | 1994,Mesosaline - 2014,Mesosaline | -0.21 | -2.53 | 0.319 |
|  | 66 | 1999,Mesosaline - 2014,Mesosaline | -0.23 | -2.70 | 0.224 |
| Whole ecosystem as metacommunity | | | | | |
| ${}^{0}\overline{\rho}$ | 1 | 1986,Hypersaline - 1994,Hypersaline | 0.01 | 5.39 | < 0.001 |
|  | 2 | 1986,Hypersaline - 1999,Hypersaline | 0.04 | 14.72 | < 0.001 |
|  | 3 | 1986,Hypersaline - 2014,Hypersaline | 0.01 | 2.39 | 0.411 |
|  | 4 | 1986,Hypersaline - 1986,Hyposaline | 0.00 | -0.68 | 0.965 |
|  | 5 | 1986,Hypersaline - 1994,Hyposaline | -0.02 | -6.98 | < 0.001 |
|  | 6 | 1986,Hypersaline - 1999,Hyposaline | -0.05 | -19.96 | < 0.001 |
|  | 7 | 1986,Hypersaline - 2014,Hyposaline | 0.00 | -1.00 | 0.975 |
|  | 8 | 1986,Hypersaline - 1986,Mesosaline | -0.05 | -17.82 | < 0.001 |
|  | 9 | 1986,Hypersaline - 1994,Mesosaline | -0.06 | -21.78 | < 0.001 |
|  | 10 | 1986,Hypersaline - 1999,Mesosaline | -0.06 | -25.22 | < 0.001 |
|  | 11 | 1986,Hypersaline - 2014,Mesosaline | -0.06 | -22.28 | < 0.001 |
|  | 12 | 1994,Hypersaline - 1999,Hypersaline | 0.02 | 9.32 | < 0.001 |
|  | 13 | 1994,Hypersaline - 2014,Hypersaline | -0.01 | -3.00 | 0.108 |
|  | 14 | 1994,Hypersaline - 1986,Hyposaline | -0.02 | -6.08 | < 0.001 |
|  | 15 | 1994,Hypersaline - 1994,Hyposaline | -0.03 | -12.38 | < 0.001 |
|  | 16 | 1994,Hypersaline - 1999,Hyposaline | -0.07 | -25.35 | < 0.001 |
|  | 17 | 1994,Hypersaline - 2014,Hyposaline | -0.02 | -6.39 | < 0.001 |
|  | 18 | 1994,Hypersaline - 1986,Mesosaline | -0.06 | -23.21 | < 0.001 |
|  | 19 | 1994,Hypersaline - 1994,Mesosaline | -0.07 | -27.18 | < 0.001 |
|  | 20 | 1994,Hypersaline - 1999,Mesosaline | -0.08 | -30.61 | < 0.001 |
|  | 21 | 1994,Hypersaline - 2014,Mesosaline | -0.07 | -27.67 | < 0.001 |
|  | 22 | 1999,Hypersaline - 2014,Hypersaline | -0.03 | -12.33 | < 0.001 |
|  | 23 | 1999,Hypersaline - 1986,Hyposaline | -0.04 | -15.40 | < 0.001 |
|  | 24 | 1999,Hypersaline - 1994,Hyposaline | -0.06 | -21.70 | < 0.001 |
|  | 25 | 1999,Hypersaline - 1999,Hyposaline | -0.09 | -34.67 | < 0.001 |
|  | 26 | 1999,Hypersaline - 2014,Hyposaline | -0.04 | -15.72 | < 0.001 |
|  | 27 | 1999,Hypersaline - 1986,Mesosaline | -0.08 | -32.53 | < 0.001 |
|  | 28 | 1999,Hypersaline - 1994,Mesosaline | -0.09 | -36.50 | < 0.001 |
|  | 29 | 1999,Hypersaline - 1999,Mesosaline | -0.10 | -39.93 | < 0.001 |
|  | 30 | 1999,Hypersaline - 2014,Mesosaline | -0.10 | -37.00 | < 0.001 |
|  | 31 | 2014,Hypersaline - 1986,Hyposaline | -0.01 | -3.08 | 0.088 |
|  | 32 | 2014,Hypersaline - 1994,Hyposaline | -0.02 | -9.37 | < 0.001 |
|  | 33 | 2014,Hypersaline - 1999,Hyposaline | -0.06 | -22.35 | < 0.001 |
|  | 34 | 2014,Hypersaline - 2014,Hyposaline | -0.01 | -3.39 | < 0.05 |
|  | 35 | 2014,Hypersaline - 1986,Mesosaline | -0.05 | -20.21 | < 0.001 |
|  | 36 | 2014,Hypersaline - 1994,Mesosaline | -0.06 | -24.17 | < 0.001 |
|  | 37 | 2014,Hypersaline - 1999,Mesosaline | -0.07 | -27.61 | < 0.001 |
|  | 38 | 2014,Hypersaline - 2014,Mesosaline | -0.06 | -24.67 | < 0.001 |
|  | 39 | 1986,Hyposaline - 1994,Hyposaline | -0.02 | -6.30 | < 0.001 |
|  | 40 | 1986,Hyposaline - 1999,Hyposaline | -0.05 | -19.27 | < 0.001 |
|  | 41 | 1986,Hyposaline - 2014,Hyposaline | 0.00 | -0.31 | < 0.001 |
|  | 42 | 1986,Hyposaline - 1986,Mesosaline | -0.04 | -17.13 | < 0.001 |
|  | 43 | 1986,Hyposaline - 1994,Mesosaline | -0.05 | -21.10 | < 0.001 |
|  | 44 | 1986,Hyposaline - 1999,Mesosaline | -0.06 | -24.53 | < 0.001 |
|  | 45 | 1986,Hyposaline - 2014,Mesosaline | -0.06 | -21.60 | < 0.001 |
|  | 46 | 1994,Hyposaline - 1999,Hyposaline | -0.03 | -12.98 | < 0.001 |
|  | 47 | 1994,Hyposaline - 2014,Hyposaline | 0.02 | 5.98 | < 0.001 |
|  | 48 | 1994,Hyposaline - 1986,Mesosaline | -0.03 | -10.83 | < 0.001 |
|  | 49 | 1994,Hyposaline - 1994,Mesosaline | -0.04 | -14.80 | < 0.001 |
|  | 50 | 1994,Hyposaline - 1999,Mesosaline | -0.05 | -18.24 | < 0.001 |
|  | 51 | 1994,Hyposaline - 2014,Mesosaline | -0.04 | -15.30 | < 0.001 |
|  | 52 | 1999,Hyposaline - 2014,Hyposaline | 0.05 | 18.96 | < 0.001 |
|  | 53 | 1999,Hyposaline - 1986,Mesosaline | 0.01 | 2.14 | 0.593 |
|  | 54 | 1999,Hyposaline - 1994,Mesosaline | 0.00 | -1.83 | 0.804 |
|  | 55 | 1999,Hyposaline - 1999,Mesosaline | -0.01 | -5.26 | < 0.001 |
|  | 56 | 1999,Hyposaline - 2014,Mesosaline | -0.01 | -2.32 | 0.459 |
|  | 57 | 2014,Hyposaline - 1986,Mesosaline | -0.04 | -16.82 | < 0.001 |
|  | 58 | 2014,Hyposaline - 1994,Mesosaline | -0.05 | -20.78 | < 0.001 |
|  | 59 | 2014,Hyposaline - 1999,Mesosaline | -0.06 | -24.22 | < 0.001 |
|  | 60 | 2014,Hyposaline - 2014,Mesosaline | -0.05 | -21.28 | < 0.001 |
|  | 61 | 1986,Mesosaline - 1994,Mesosaline | -0.01 | -3.97 | < 0.05 |
|  | 62 | 1986,Mesosaline - 1999,Mesosaline | -0.02 | -7.40 | < 0.001 |
|  | 63 | 1986,Mesosaline - 2014,Mesosaline | -0.01 | -4.46 | < 0.001 |
|  | 64 | 1994,Mesosaline - 1999,Mesosaline | -0.01 | -3.43 | < 0.05 |
|  | 65 | 1994,Mesosaline - 2014,Mesosaline | 0.00 | -0.50 | 0.975 |
|  | 66 | 1999,Mesosaline - 2014,Mesosaline | 0.01 | 2.94 | 0.128 |
| ${}^{1}\overline{\rho}$ | 1 | 1986,Hypersaline - 1994,Hypersaline | -0.03 | -7.72 | < 0.001 |
|  | 2 | 1986,Hypersaline - 1999,Hypersaline | -0.05 | -12.15 | < 0.001 |
|  | 3 | 1986,Hypersaline - 2014,Hypersaline | -0.09 | -21.66 | < 0.001 |
|  | 4 | 1986,Hypersaline - 1986,Hyposaline | 0.03 | 6.12 | < 0.001 |
|  | 5 | 1986,Hypersaline - 1994,Hyposaline | 0.01 | 3.00 | 0.108 |
|  | 6 | 1986,Hypersaline - 1999,Hyposaline | -0.02 | -3.81 | < 0.01 |
|  | 7 | 1986,Hypersaline - 2014,Hyposaline | 0.04 | 9.56 | < 0.001 |
|  | 8 | 1986,Hypersaline - 1986,Mesosaline | -0.16 | -37.69 | < 0.001 |
|  | 9 | 1986,Hypersaline - 1994,Mesosaline | -0.16 | -39.03 | < 0.001 |
|  | 10 | 1986,Hypersaline - 1999,Mesosaline | -0.17 | -40.24 | < 0.001 |
|  | 11 | 1986,Hypersaline - 2014,Mesosaline | -0.17 | -42.36 | < 0.001 |
|  | 12 | 1994,Hypersaline - 1999,Hypersaline | -0.02 | -4.43 | < 0.001 |
|  | 13 | 1994,Hypersaline - 2014,Hypersaline | -0.06 | -13.93 | < 0.001 |
|  | 14 | 1994,Hypersaline - 1986,Hyposaline | 0.06 | 13.85 | < 0.001 |
|  | 15 | 1994,Hypersaline - 1994,Hyposaline | 0.04 | 10.73 | < 0.001 |
|  | 16 | 1994,Hypersaline - 1999,Hyposaline | 0.02 | 3.92 | < 0.01 |
|  | 17 | 1994,Hypersaline - 2014,Hyposaline | 0.07 | 17.28 | < 0.001 |
|  | 18 | 1994,Hypersaline - 1986,Mesosaline | -0.12 | -29.97 | < 0.001 |
|  | 19 | 1994,Hypersaline - 1994,Mesosaline | -0.13 | -31.30 | < 0.001 |
|  | 20 | 1994,Hypersaline - 1999,Mesosaline | -0.13 | -32.52 | < 0.001 |
|  | 21 | 1994,Hypersaline - 2014,Mesosaline | -0.14 | -34.64 | < 0.001 |
|  | 22 | 1999,Hypersaline - 2014,Hypersaline | -0.04 | -9.51 | < 0.001 |
|  | 23 | 1999,Hypersaline - 1986,Hyposaline | 0.08 | 18.27 | < 0.001 |
|  | 24 | 1999,Hypersaline - 1994,Hyposaline | 0.06 | 15.15 | < 0.001 |
|  | 25 | 1999,Hypersaline - 1999,Hyposaline | 0.03 | 8.34 | < 0.001 |
|  | 26 | 1999,Hypersaline - 2014,Hyposaline | 0.09 | 21.71 | < 0.001 |
|  | 27 | 1999,Hypersaline - 1986,Mesosaline | -0.11 | -25.54 | < 0.001 |
|  | 28 | 1999,Hypersaline - 1994,Mesosaline | -0.11 | -26.88 | < 0.001 |
|  | 29 | 1999,Hypersaline - 1999,Mesosaline | -0.12 | -28.09 | < 0.001 |
|  | 30 | 1999,Hypersaline - 2014,Mesosaline | -0.12 | -30.21 | < 0.001 |
|  | 31 | 2014,Hypersaline - 1986,Hyposaline | 0.11 | 27.78 | < 0.001 |
|  | 32 | 2014,Hypersaline - 1994,Hyposaline | 0.10 | 24.66 | < 0.001 |
|  | 33 | 2014,Hypersaline - 1999,Hyposaline | 0.07 | 17.85 | < 0.001 |
|  | 34 | 2014,Hypersaline - 2014,Hyposaline | 0.13 | 31.22 | < 0.001 |
|  | 35 | 2014,Hypersaline - 1986,Mesosaline | -0.07 | -16.03 | < 0.001 |
|  | 36 | 2014,Hypersaline - 1994,Mesosaline | -0.07 | -17.37 | < 0.001 |
|  | 37 | 2014,Hypersaline - 1999,Mesosaline | -0.08 | -18.59 | < 0.001 |
|  | 38 | 2014,Hypersaline - 2014,Mesosaline | -0.09 | -20.70 | < 0.001 |
|  | 39 | 1986,Hyposaline - 1994,Hyposaline | -0.01 | -3.12 | 0.077 |
|  | 40 | 1986,Hyposaline - 1999,Hyposaline | -0.04 | -9.93 | < 0.001 |
|  | 41 | 1986,Hyposaline - 2014,Hyposaline | 0.01 | 3.43 | < 0.05 |
|  | 42 | 1986,Hyposaline - 1986,Mesosaline | -0.18 | -43.82 | < 0.001 |
|  | 43 | 1986,Hyposaline - 1994,Mesosaline | -0.19 | -45.15 | < 0.001 |
|  | 44 | 1986,Hyposaline - 1999,Mesosaline | -0.19 | -46.37 | < 0.001 |
|  | 45 | 1986,Hyposaline - 2014,Mesosaline | -0.20 | -48.48 | < 0.001 |
|  | 46 | 1994,Hyposaline - 1999,Hyposaline | -0.03 | -6.81 | < 0.001 |
|  | 47 | 1994,Hyposaline - 2014,Hyposaline | 0.03 | 6.56 | < 0.001 |
|  | 48 | 1994,Hyposaline - 1986,Mesosaline | -0.17 | -40.69 | < 0.001 |
|  | 49 | 1994,Hyposaline - 1994,Mesosaline | -0.17 | -42.03 | < 0.001 |
|  | 50 | 1994,Hyposaline - 1999,Mesosaline | -0.18 | -43.24 | < 0.001 |
|  | 51 | 1994,Hyposaline - 2014,Mesosaline | -0.19 | -45.36 | < 0.001 |
|  | 52 | 1999,Hyposaline - 2014,Hyposaline | 0.06 | 13.37 | < 0.001 |
|  | 53 | 1999,Hyposaline - 1986,Mesosaline | -0.14 | -33.88 | < 0.001 |
|  | 54 | 1999,Hyposaline - 1994,Mesosaline | -0.15 | -35.22 | < 0.001 |
|  | 55 | 1999,Hyposaline - 1999,Mesosaline | -0.15 | -36.44 | < 0.001 |
|  | 56 | 1999,Hyposaline - 2014,Mesosaline | -0.16 | -38.55 | < 0.001 |
|  | 57 | 2014,Hyposaline - 1986,Mesosaline | -0.19 | -47.25 | < 0.001 |
|  | 58 | 2014,Hyposaline - 1994,Mesosaline | -0.20 | -48.59 | < 0.001 |
|  | 59 | 2014,Hyposaline - 1999,Mesosaline | -0.21 | -49.80 | < 0.001 |
|  | 60 | 2014,Hyposaline - 2014,Mesosaline | -0.21 | -51.92 | < 0.001 |
|  | 61 | 1986,Mesosaline - 1994,Mesosaline | -0.01 | -1.34 | 0.974 |
|  | 62 | 1986,Mesosaline - 1999,Mesosaline | -0.01 | -2.55 | 0.308 |
|  | 63 | 1986,Mesosaline - 2014,Mesosaline | -0.02 | -4.67 | < 0.001 |
|  | 64 | 1994,Mesosaline - 1999,Mesosaline | -0.01 | -1.22 | 0.988 |
|  | 65 | 1994,Mesosaline - 2014,Mesosaline | -0.01 | -3.33 | < 0.05 |
|  | 66 | 1999,Mesosaline - 2014,Mesosaline | -0.01 | -2.12 | 0.611 |
| ${}^{2}\overline{\rho}$ | 1 | 1986,Hypersaline - 1994,Hypersaline | -0.03 | -5.67 | < 0.001 |
|  | 2 | 1986,Hypersaline - 1999,Hypersaline | -0.07 | -13.84 | < 0.001 |
|  | 3 | 1986,Hypersaline - 2014,Hypersaline | -0.11 | -21.78 | < 0.001 |
|  | 4 | 1986,Hypersaline - 1986,Hyposaline | 0.07 | 14.53 | < 0.001 |
|  | 5 | 1986,Hypersaline - 1994,Hyposaline | 0.06 | 11.45 | < 0.001 |
|  | 6 | 1986,Hypersaline - 1999,Hyposaline | 0.04 | 7.44 | < 0.001 |
|  | 7 | 1986,Hypersaline - 2014,Hyposaline | 0.10 | 19.99 | < 0.001 |
|  | 8 | 1986,Hypersaline - 1986,Mesosaline | -0.15 | -29.32 | < 0.001 |
|  | 9 | 1986,Hypersaline - 1994,Mesosaline | -0.15 | -30.16 | < 0.001 |
|  | 10 | 1986,Hypersaline - 1999,Mesosaline | -0.15 | -30.78 | < 0.001 |
|  | 11 | 1986,Hypersaline - 2014,Mesosaline | -0.17 | -33.87 | < 0.001 |
|  | 12 | 1994,Hypersaline - 1999,Hypersaline | -0.04 | -8.17 | < 0.001 |
|  | 13 | 1994,Hypersaline - 2014,Hypersaline | -0.08 | -16.11 | < 0.001 |
|  | 14 | 1994,Hypersaline - 1986,Hyposaline | 0.10 | 20.20 | < 0.001 |
|  | 15 | 1994,Hypersaline - 1994,Hyposaline | 0.09 | 17.12 | < 0.001 |
|  | 16 | 1994,Hypersaline - 1999,Hyposaline | 0.07 | 13.11 | < 0.001 |
|  | 17 | 1994,Hypersaline - 2014,Hyposaline | 0.13 | 25.66 | < 0.001 |
|  | 18 | 1994,Hypersaline - 1986,Mesosaline | -0.12 | -23.64 | < 0.001 |
|  | 19 | 1994,Hypersaline - 1994,Mesosaline | -0.12 | -24.49 | < 0.001 |
|  | 20 | 1994,Hypersaline - 1999,Mesosaline | -0.13 | -25.10 | < 0.001 |
|  | 21 | 1994,Hypersaline - 2014,Mesosaline | -0.14 | -28.19 | < 0.001 |
|  | 22 | 1999,Hypersaline - 2014,Hypersaline | -0.04 | -7.94 | < 0.001 |
|  | 23 | 1999,Hypersaline - 1986,Hyposaline | 0.14 | 28.37 | < 0.001 |
|  | 24 | 1999,Hypersaline - 1994,Hyposaline | 0.13 | 25.29 | < 0.001 |
|  | 25 | 1999,Hypersaline - 1999,Hyposaline | 0.11 | 21.28 | < 0.001 |
|  | 26 | 1999,Hypersaline - 2014,Hyposaline | 0.17 | 33.83 | < 0.001 |
|  | 27 | 1999,Hypersaline - 1986,Mesosaline | -0.08 | -15.47 | < 0.001 |
|  | 28 | 1999,Hypersaline - 1994,Mesosaline | -0.08 | -16.32 | < 0.001 |
|  | 29 | 1999,Hypersaline - 1999,Mesosaline | -0.08 | -16.93 | < 0.001 |
|  | 30 | 1999,Hypersaline - 2014,Mesosaline | -0.10 | -20.02 | < 0.001 |
|  | 31 | 2014,Hypersaline - 1986,Hyposaline | 0.18 | 36.32 | < 0.001 |
|  | 32 | 2014,Hypersaline - 1994,Hyposaline | 0.17 | 33.23 | < 0.001 |
|  | 33 | 2014,Hypersaline - 1999,Hyposaline | 0.15 | 29.22 | < 0.001 |
|  | 34 | 2014,Hypersaline - 2014,Hyposaline | 0.21 | 41.77 | < 0.001 |
|  | 35 | 2014,Hypersaline - 1986,Mesosaline | -0.04 | -7.53 | < 0.001 |
|  | 36 | 2014,Hypersaline - 1994,Mesosaline | -0.04 | -8.37 | < 0.001 |
|  | 37 | 2014,Hypersaline - 1999,Mesosaline | -0.05 | -8.99 | < 0.001 |
|  | 38 | 2014,Hypersaline - 2014,Mesosaline | -0.06 | -12.08 | < 0.001 |
|  | 39 | 1986,Hyposaline - 1994,Hyposaline | -0.02 | -3.08 | 0.086 |
|  | 40 | 1986,Hyposaline - 1999,Hyposaline | -0.04 | -7.09 | < 0.001 |
|  | 41 | 1986,Hyposaline - 2014,Hyposaline | 0.03 | 5.45 | < 0.001 |
|  | 42 | 1986,Hyposaline - 1986,Mesosaline | -0.22 | -43.85 | < 0.001 |
|  | 43 | 1986,Hyposaline - 1994,Mesosaline | -0.22 | -44.69 | < 0.001 |
|  | 44 | 1986,Hyposaline - 1999,Mesosaline | -0.23 | -45.31 | < 0.001 |
|  | 45 | 1986,Hyposaline - 2014,Mesosaline | -0.24 | -48.40 | < 0.001 |
|  | 46 | 1994,Hyposaline - 1999,Hyposaline | -0.02 | -4.01 | < 0.01 |
|  | 47 | 1994,Hyposaline - 2014,Hyposaline | 0.04 | 8.54 | < 0.001 |
|  | 48 | 1994,Hyposaline - 1986,Mesosaline | -0.20 | -40.76 | < 0.001 |
|  | 49 | 1994,Hyposaline - 1994,Mesosaline | -0.21 | -41.61 | < 0.001 |
|  | 50 | 1994,Hyposaline - 1999,Mesosaline | -0.21 | -42.23 | < 0.001 |
|  | 51 | 1994,Hyposaline - 2014,Mesosaline | -0.23 | -45.31 | < 0.001 |
|  | 52 | 1999,Hyposaline - 2014,Hyposaline | 0.06 | 12.55 | < 0.001 |
|  | 53 | 1999,Hyposaline - 1986,Mesosaline | -0.18 | -36.75 | < 0.001 |
|  | 54 | 1999,Hyposaline - 1994,Mesosaline | -0.19 | -37.60 | < 0.001 |
|  | 55 | 1999,Hyposaline - 1999,Mesosaline | -0.19 | -38.21 | < 0.001 |
|  | 56 | 1999,Hyposaline - 2014,Mesosaline | -0.21 | -41.30 | < 0.001 |
|  | 57 | 2014,Hyposaline - 1986,Mesosaline | -0.25 | -49.30 | < 0.001 |
|  | 58 | 2014,Hyposaline - 1994,Mesosaline | -0.25 | -50.15 | < 0.001 |
|  | 59 | 2014,Hyposaline - 1999,Mesosaline | -0.25 | -50.76 | < 0.001 |
|  | 60 | 2014,Hyposaline - 2014,Mesosaline | -0.27 | -53.85 | < 0.001 |
|  | 61 | 1986,Mesosaline - 1994,Mesosaline | 0.00 | -0.84 | 0.985 |
|  | 62 | 1986,Mesosaline - 1999,Mesosaline | -0.01 | -1.46 | 0.951 |
|  | 63 | 1986,Mesosaline - 2014,Mesosaline | -0.02 | -4.55 | < 0.001 |
|  | 64 | 1994,Mesosaline - 1999,Mesosaline | 0.00 | -0.62 | 0.982 |
|  | 65 | 1994,Mesosaline - 2014,Mesosaline | -0.02 | -3.71 | < 0.05 |
|  | 66 | 1999,Mesosaline - 2014,Mesosaline | -0.02 | -3.09 | 0.085 |
| ${}^{0}\gamma$ | 1 | 1986,Hypersaline - 1994,Hypersaline | 2.60 | 1.86 | 0.786 |
|  | 2 | 1986,Hypersaline - 1999,Hypersaline | 3.62 | 2.59 | 0.285 |
|  | 3 | 1986,Hypersaline - 2014,Hypersaline | 6.06 | 4.33 | < 0.001 |
|  | 4 | 1986,Hypersaline - 1986,Hyposaline | -53.77 | -38.42 | < 0.001 |
|  | 5 | 1986,Hypersaline - 1994,Hyposaline | -35.07 | -25.06 | < 0.001 |
|  | 6 | 1986,Hypersaline - 1999,Hyposaline | -31.87 | -22.77 | < 0.001 |
|  | 7 | 1986,Hypersaline - 2014,Hyposaline | -39.30 | -28.08 | < 0.001 |
|  | 8 | 1986,Hypersaline - 1986,Mesosaline | 3.20 | 2.28 | 0.488 |
|  | 9 | 1986,Hypersaline - 1994,Mesosaline | 4.18 | 2.99 | 0.112 |
|  | 10 | 1986,Hypersaline - 1999,Mesosaline | 3.61 | 2.58 | 0.289 |
|  | 11 | 1986,Hypersaline - 2014,Mesosaline | -0.79 | -0.56 | 0.989 |
|  | 12 | 1994,Hypersaline - 1999,Hypersaline | 1.03 | 0.73 | 0.982 |
|  | 13 | 1994,Hypersaline - 2014,Hypersaline | 3.46 | 2.47 | 0.356 |
|  | 14 | 1994,Hypersaline - 1986,Hyposaline | -56.37 | -40.28 | < 0.001 |
|  | 15 | 1994,Hypersaline - 1994,Hyposaline | -37.67 | -26.92 | < 0.001 |
|  | 16 | 1994,Hypersaline - 1999,Hyposaline | -34.47 | -24.63 | < 0.001 |
|  | 17 | 1994,Hypersaline - 2014,Hyposaline | -41.90 | -29.94 | < 0.001 |
|  | 18 | 1994,Hypersaline - 1986,Mesosaline | 0.60 | 0.43 | 0.992 |
|  | 19 | 1994,Hypersaline - 1994,Mesosaline | 1.58 | 1.13 | 0.993 |
|  | 20 | 1994,Hypersaline - 1999,Mesosaline | 1.02 | 0.73 | 0.994 |
|  | 21 | 1994,Hypersaline - 2014,Mesosaline | -3.38 | -2.42 | 0.393 |
|  | 22 | 1999,Hypersaline - 2014,Hypersaline | 2.44 | 1.74 | 0.849 |
|  | 23 | 1999,Hypersaline - 1986,Hyposaline | -57.39 | -41.01 | < 0.001 |
|  | 24 | 1999,Hypersaline - 1994,Hyposaline | -38.70 | -27.65 | < 0.001 |
|  | 25 | 1999,Hypersaline - 1999,Hyposaline | -35.50 | -25.36 | < 0.001 |
|  | 26 | 1999,Hypersaline - 2014,Hyposaline | -42.92 | -30.67 | < 0.001 |
|  | 27 | 1999,Hypersaline - 1986,Mesosaline | -0.43 | -0.31 | 0.988 |
|  | 28 | 1999,Hypersaline - 1994,Mesosaline | 0.56 | 0.40 | 9.975 |
|  | 29 | 1999,Hypersaline - 1999,Mesosaline | -0.01 | -0.01 | 0.956 |
|  | 30 | 1999,Hypersaline - 2014,Mesosaline | -4.41 | -3.15 | 0.071 |
|  | 31 | 2014,Hypersaline - 1986,Hyposaline | -59.83 | -42.75 | < 0.001 |
|  | 32 | 2014,Hypersaline - 1994,Hyposaline | -41.13 | -29.39 | < 0.001 |
|  | 33 | 2014,Hypersaline - 1999,Hyposaline | -37.93 | -27.11 | < 0.001 |
|  | 34 | 2014,Hypersaline - 2014,Hyposaline | -45.36 | -32.41 | < 0.001 |
|  | 35 | 2014,Hypersaline - 1986,Mesosaline | -2.86 | -2.05 | 0.661 |
|  | 36 | 2014,Hypersaline - 1994,Mesosaline | -1.88 | -1.34 | 0.973 |
|  | 37 | 2014,Hypersaline - 1999,Mesosaline | -2.45 | -1.75 | 0.846 |
|  | 38 | 2014,Hypersaline - 2014,Mesosaline | -6.85 | -4.89 | < 0.001 |
|  | 39 | 1986,Hyposaline - 1994,Hyposaline | 18.69 | 13.36 | < 0.001 |
|  | 40 | 1986,Hyposaline - 1999,Hyposaline | 21.90 | 15.65 | < 0.001 |
|  | 41 | 1986,Hyposaline - 2014,Hyposaline | 14.47 | 10.34 | < 0.001 |
|  | 42 | 1986,Hyposaline - 1986,Mesosaline | 56.96 | 40.70 | < 0.001 |
|  | 43 | 1986,Hyposaline - 1994,Mesosaline | 57.95 | 41.41 | < 0.001 |
|  | 44 | 1986,Hyposaline - 1999,Mesosaline | 57.38 | 41.00 | < 0.001 |
|  | 45 | 1986,Hyposaline - 2014,Mesosaline | 52.98 | 37.86 | < 0.001 |
|  | 46 | 1994,Hyposaline - 1999,Hyposaline | 3.20 | 2.29 | 0.486 |
|  | 47 | 1994,Hyposaline - 2014,Hyposaline | -4.23 | -3.02 | 0.102 |
|  | 48 | 1994,Hyposaline - 1986,Mesosaline | 38.27 | 27.34 | < 0.001 |
|  | 49 | 1994,Hyposaline - 1994,Mesosaline | 39.25 | 28.05 | < 0.001 |
|  | 50 | 1994,Hyposaline - 1999,Mesosaline | 38.69 | 27.64 | < 0.001 |
|  | 51 | 1994,Hyposaline - 2014,Mesosaline | 34.29 | 24.50 | < 0.001 |
|  | 52 | 1999,Hyposaline - 2014,Hyposaline | -7.43 | -5.31 | < 0.001 |
|  | 53 | 1999,Hyposaline - 1986,Mesosaline | 35.07 | 25.06 | < 0.001 |
|  | 54 | 1999,Hyposaline - 1994,Mesosaline | 36.05 | 25.76 | < 0.001 |
|  | 55 | 1999,Hyposaline - 1999,Mesosaline | 35.49 | 25.36 | < 0.001 |
|  | 56 | 1999,Hyposaline - 2014,Mesosaline | 31.09 | 22.21 | < 0.001 |
|  | 57 | 2014,Hyposaline - 1986,Mesosaline | 42.50 | 30.37 | < 0.001 |
|  | 58 | 2014,Hyposaline - 1994,Mesosaline | 43.48 | 31.07 | < 0.001 |
|  | 59 | 2014,Hyposaline - 1999,Mesosaline | 42.91 | 30.66 | < 0.001 |
|  | 60 | 2014,Hyposaline - 2014,Mesosaline | 38.51 | 27.52 | < 0.001 |
|  | 61 | 1986,Mesosaline - 1994,Mesosaline | 0.98 | 0.70 | 0.983 |
|  | 62 | 1986,Mesosaline - 1999,Mesosaline | 0.42 | 0.30 | 0.975 |
|  | 63 | 1986,Mesosaline - 2014,Mesosaline | -3.98 | -2.85 | 0.161 |
|  | 64 | 1994,Mesosaline - 1999,Mesosaline | -0.57 | -0.40 | 0.992 |
|  | 65 | 1994,Mesosaline - 2014,Mesosaline | -4.97 | -3.55 | < 0.05 |
|  | 66 | 1999,Mesosaline - 2014,Mesosaline | -4.40 | -3.14 | 0.072 |
| ${}^{1}\gamma$ | 1 | 1986,Hypersaline - 1994,Hypersaline | 4.05 | 9.60 | < 0.001 |
|  | 2 | 1986,Hypersaline - 1999,Hypersaline | 4.19 | 9.95 | < 0.001 |
|  | 3 | 1986,Hypersaline - 2014,Hypersaline | 3.58 | 8.48 | < 0.001 |
|  | 4 | 1986,Hypersaline - 1986,Hyposaline | -8.64 | -20.48 | < 0.001 |
|  | 5 | 1986,Hypersaline - 1994,Hyposaline | -7.29 | -17.28 | < 0.001 |
|  | 6 | 1986,Hypersaline - 1999,Hyposaline | -2.89 | -6.85 | < 0.001 |
|  | 7 | 1986,Hypersaline - 2014,Hyposaline | 0.45 | 1.06 | 0.994 |
|  | 8 | 1986,Hypersaline - 1986,Mesosaline | 3.71 | 8.79 | < 0.001 |
|  | 9 | 1986,Hypersaline - 1994,Mesosaline | 3.73 | 8.85 | < 0.001 |
|  | 10 | 1986,Hypersaline - 1999,Mesosaline | 3.78 | 8.97 | < 0.001 |
|  | 11 | 1986,Hypersaline - 2014,Mesosaline | 3.36 | 7.97 | < 0.001 |
|  | 12 | 1994,Hypersaline - 1999,Hypersaline | 0.15 | 0.35 | 0.994 |
|  | 13 | 1994,Hypersaline - 2014,Hypersaline | -0.47 | -1.12 | 0.994 |
|  | 14 | 1994,Hypersaline - 1986,Hyposaline | -12.68 | -30.08 | < 0.001 |
|  | 15 | 1994,Hypersaline - 1994,Hyposaline | -11.33 | -26.88 | < 0.001 |
|  | 16 | 1994,Hypersaline - 1999,Hyposaline | -6.94 | -16.45 | < 0.001 |
|  | 17 | 1994,Hypersaline - 2014,Hyposaline | -3.60 | -8.54 | < 0.001 |
|  | 18 | 1994,Hypersaline - 1986,Mesosaline | -0.34 | -0.81 | 0.992 |
|  | 19 | 1994,Hypersaline - 1994,Mesosaline | -0.32 | -0.75 | 0.994 |
|  | 20 | 1994,Hypersaline - 1999,Mesosaline | -0.26 | -0.63 | 0.994 |
|  | 21 | 1994,Hypersaline - 2014,Mesosaline | -0.69 | -1.63 | 0.898 |
|  | 22 | 1999,Hypersaline - 2014,Hypersaline | -0.62 | -1.47 | 0.949 |
|  | 23 | 1999,Hypersaline - 1986,Hyposaline | -12.83 | -30.42 | < 0.001 |
|  | 24 | 1999,Hypersaline - 1994,Hyposaline | -11.48 | -27.22 | < 0.001 |
|  | 25 | 1999,Hypersaline - 1999,Hyposaline | -7.08 | -16.80 | < 0.001 |
|  | 26 | 1999,Hypersaline - 2014,Hyposaline | -3.75 | -8.89 | < 0.001 |
|  | 27 | 1999,Hypersaline - 1986,Mesosaline | -0.49 | -1.16 | 0.992 |
|  | 28 | 1999,Hypersaline - 1994,Mesosaline | -0.46 | -1.10 | 0.995 |
|  | 29 | 1999,Hypersaline - 1999,Mesosaline | -0.41 | -0.97 | 0.992 |
|  | 30 | 1999,Hypersaline - 2014,Mesosaline | -0.83 | -1.98 | 0.709 |
|  | 31 | 2014,Hypersaline - 1986,Hyposaline | -12.21 | -28.96 | < 0.001 |
|  | 32 | 2014,Hypersaline - 1994,Hyposaline | -10.86 | -25.76 | < 0.001 |
|  | 33 | 2014,Hypersaline - 1999,Hyposaline | -6.46 | -15.33 | < 0.001 |
|  | 34 | 2014,Hypersaline - 2014,Hyposaline | -3.13 | -7.42 | < 0.001 |
|  | 35 | 2014,Hypersaline - 1986,Mesosaline | 0.13 | 0.31 | 0.994 |
|  | 36 | 2014,Hypersaline - 1994,Mesosaline | 0.15 | 0.37 | 0.992 |
|  | 37 | 2014,Hypersaline - 1999,Mesosaline | 0.21 | 0.49 | 0.994 |
|  | 38 | 2014,Hypersaline - 2014,Mesosaline | -0.22 | -0.51 | 0.995 |
|  | 39 | 1986,Hyposaline - 1994,Hyposaline | 1.35 | 3.20 | 0.062 |
|  | 40 | 1986,Hyposaline - 1999,Hyposaline | 5.75 | 13.63 | < 0.001 |
|  | 41 | 1986,Hyposaline - 2014,Hyposaline | 9.08 | 21.54 | < 0.001 |
|  | 42 | 1986,Hyposaline - 1986,Mesosaline | 12.34 | 29.27 | < 0.001 |
|  | 43 | 1986,Hyposaline - 1994,Mesosaline | 12.37 | 29.32 | < 0.001 |
|  | 44 | 1986,Hyposaline - 1999,Mesosaline | 12.42 | 29.45 | < 0.001 |
|  | 45 | 1986,Hyposaline - 2014,Mesosaline | 12.00 | 28.45 | < 0.001 |
|  | 46 | 1994,Hyposaline - 1999,Hyposaline | 4.40 | 10.43 | < 0.001 |
|  | 47 | 1994,Hyposaline - 2014,Hyposaline | 7.73 | 18.34 | < 0.001 |
|  | 48 | 1994,Hyposaline - 1986,Mesosaline | 10.99 | 26.07 | < 0.001 |
|  | 49 | 1994,Hyposaline - 1994,Mesosaline | 11.02 | 26.12 | < 0.001 |
|  | 50 | 1994,Hyposaline - 1999,Mesosaline | 11.07 | 26.25 | < 0.001 |
|  | 51 | 1994,Hyposaline - 2014,Mesosaline | 10.65 | 25.25 | < 0.001 |
|  | 52 | 1999,Hyposaline - 2014,Hyposaline | 3.34 | 7.91 | < 0.001 |
|  | 53 | 1999,Hyposaline - 1986,Mesosaline | 6.60 | 15.64 | < 0.001 |
|  | 54 | 1999,Hyposaline - 1994,Mesosaline | 6.62 | 15.70 | < 0.001 |
|  | 55 | 1999,Hyposaline - 1999,Mesosaline | 6.67 | 15.82 | < 0.001 |
|  | 56 | 1999,Hyposaline - 2014,Mesosaline | 6.25 | 14.82 | < 0.001 |
|  | 57 | 2014,Hyposaline - 1986,Mesosaline | 3.26 | 7.73 | < 0.001 |
|  | 58 | 2014,Hyposaline - 1994,Mesosaline | 3.28 | 7.79 | < 0.001 |
|  | 59 | 2014,Hyposaline - 1999,Mesosaline | 3.34 | 7.91 | < 0.001 |
|  | 60 | 2014,Hyposaline - 2014,Mesosaline | 2.91 | 6.91 | < 0.001 |
|  | 61 | 1986,Mesosaline - 1994,Mesosaline | 0.02 | 0.05 | 0.995 |
|  | 62 | 1986,Mesosaline - 1999,Mesosaline | 0.08 | 0.18 | 0.995 |
|  | 63 | 1986,Mesosaline - 2014,Mesosaline | -0.35 | -0.82 | 0.994 |
|  | 64 | 1994,Mesosaline - 1999,Mesosaline | 0.05 | 0.13 | 0.996 |
|  | 65 | 1994,Mesosaline - 2014,Mesosaline | -0.37 | -0.88 | 0.997 |
|  | 66 | 1999,Mesosaline - 2014,Mesosaline | -0.42 | -1.00 | 0.995 |
| ${}^{2}\gamma$ | 1 | 1986,Hypersaline - 1994,Hypersaline | 1.23 | 6.28 | < 0.001 |
|  | 2 | 1986,Hypersaline - 1999,Hypersaline | 1.30 | 6.62 | < 0.001 |
|  | 3 | 1986,Hypersaline - 2014,Hypersaline | 1.04 | 5.29 | < 0.001 |
|  | 4 | 1986,Hypersaline - 1986,Hyposaline | -3.63 | -18.51 | < 0.001 |
|  | 5 | 1986,Hypersaline - 1994,Hyposaline | -2.88 | -14.67 | < 0.001 |
|  | 6 | 1986,Hypersaline - 1999,Hyposaline | -1.92 | -9.79 | < 0.001 |
|  | 7 | 1986,Hypersaline - 2014,Hyposaline | 0.16 | 0.84 | 0.995 |
|  | 8 | 1986,Hypersaline - 1986,Mesosaline | 0.84 | 4.26 | < 0.01 |
|  | 9 | 1986,Hypersaline - 1994,Mesosaline | 0.87 | 4.43 | < 0.001 |
|  | 10 | 1986,Hypersaline - 1999,Mesosaline | 0.90 | 4.59 | < 0.001 |
|  | 11 | 1986,Hypersaline - 2014,Mesosaline | 0.72 | 3.68 | < 0.05 |
|  | 12 | 1994,Hypersaline - 1999,Hypersaline | 0.07 | 0.34 | 0.995 |
|  | 13 | 1994,Hypersaline - 2014,Hypersaline | -0.20 | -0.99 | 0.996 |
|  | 14 | 1994,Hypersaline - 1986,Hyposaline | -4.86 | -24.79 | < 0.001 |
|  | 15 | 1994,Hypersaline - 1994,Hyposaline | -4.11 | -20.95 | < 0.001 |
|  | 16 | 1994,Hypersaline - 1999,Hyposaline | -3.15 | -16.07 | < 0.001 |
|  | 17 | 1994,Hypersaline - 2014,Hyposaline | -1.07 | -5.44 | < 0.001 |
|  | 18 | 1994,Hypersaline - 1986,Mesosaline | -0.40 | -2.02 | 0.679 |
|  | 19 | 1994,Hypersaline - 1994,Mesosaline | -0.36 | -1.85 | 0.789 |
|  | 20 | 1994,Hypersaline - 1999,Mesosaline | -0.33 | -1.69 | 0.875 |
|  | 21 | 1994,Hypersaline - 2014,Mesosaline | -0.51 | -2.60 | 0.281 |
|  | 22 | 1999,Hypersaline - 2014,Hypersaline | -0.26 | -1.34 | 0.975 |
|  | 23 | 1999,Hypersaline - 1986,Hyposaline | -4.93 | -25.13 | < 0.001 |
|  | 24 | 1999,Hypersaline - 1994,Hyposaline | -4.17 | -21.29 | < 0.001 |
|  | 25 | 1999,Hypersaline - 1999,Hyposaline | -3.22 | -16.41 | < 0.001 |
|  | 26 | 1999,Hypersaline - 2014,Hyposaline | -1.13 | -5.78 | < 0.001 |
|  | 27 | 1999,Hypersaline - 1986,Mesosaline | -0.46 | -2.36 | 0.433 |
|  | 28 | 1999,Hypersaline - 1994,Mesosaline | -0.43 | -2.19 | 0.555 |
|  | 29 | 1999,Hypersaline - 1999,Mesosaline | -0.40 | -2.03 | 0.676 |
|  | 30 | 1999,Hypersaline - 2014,Mesosaline | -0.58 | -2.94 | 0.127 |
|  | 31 | 2014,Hypersaline - 1986,Hyposaline | -4.67 | -23.79 | < 0.001 |
|  | 32 | 2014,Hypersaline - 1994,Hyposaline | -3.91 | -19.95 | < 0.001 |
|  | 33 | 2014,Hypersaline - 1999,Hyposaline | -2.96 | -15.07 | < 0.001 |
|  | 34 | 2014,Hypersaline - 2014,Hyposaline | -0.87 | -4.45 | < 0.001 |
|  | 35 | 2014,Hypersaline - 1986,Mesosaline | -0.20 | -1.03 | 0.996 |
|  | 36 | 2014,Hypersaline - 1994,Mesosaline | -0.17 | -0.86 | 0.995 |
|  | 37 | 2014,Hypersaline - 1999,Mesosaline | -0.14 | -0.69 | 0.995 |
|  | 38 | 2014,Hypersaline - 2014,Mesosaline | -0.31 | -1.60 | 0.908 |
|  | 39 | 1986,Hyposaline - 1994,Hyposaline | 0.75 | 3.84 | < 0.01 |
|  | 40 | 1986,Hyposaline - 1999,Hyposaline | 1.71 | 8.72 | < 0.001 |
|  | 41 | 1986,Hyposaline - 2014,Hyposaline | 3.79 | 19.34 | < 0.001 |
|  | 42 | 1986,Hyposaline - 1986,Mesosaline | 4.46 | 22.77 | < 0.001 |
|  | 43 | 1986,Hyposaline - 1994,Mesosaline | 4.50 | 22.93 | < 0.001 |
|  | 44 | 1986,Hyposaline - 1999,Mesosaline | 4.53 | 23.10 | < 0.001 |
|  | 45 | 1986,Hyposaline - 2014,Mesosaline | 4.35 | 22.19 | < 0.001 |
|  | 46 | 1994,Hyposaline - 1999,Hyposaline | 0.96 | 4.88 | < 0.001 |
|  | 47 | 1994,Hyposaline - 2014,Hyposaline | 3.04 | 15.50 | < 0.001 |
|  | 48 | 1994,Hyposaline - 1986,Mesosaline | 3.71 | 18.93 | < 0.001 |
|  | 49 | 1994,Hyposaline - 1994,Mesosaline | 3.74 | 19.10 | < 0.001 |
|  | 50 | 1994,Hyposaline - 1999,Mesosaline | 3.78 | 19.26 | < 0.001 |
|  | 51 | 1994,Hyposaline - 2014,Mesosaline | 3.60 | 18.35 | < 0.001 |
|  | 52 | 1999,Hyposaline - 2014,Hyposaline | 2.08 | 10.62 | < 0.001 |
|  | 53 | 1999,Hyposaline - 1986,Mesosaline | 2.75 | 14.05 | < 0.001 |
|  | 54 | 1999,Hyposaline - 1994,Mesosaline | 2.79 | 14.21 | < 0.001 |
|  | 55 | 1999,Hyposaline - 1999,Mesosaline | 2.82 | 14.38 | < 0.001 |
|  | 56 | 1999,Hyposaline - 2014,Mesosaline | 2.64 | 13.47 | < 0.001 |
|  | 57 | 2014,Hyposaline - 1986,Mesosaline | 0.67 | 3.42 | < 0.05 |
|  | 58 | 2014,Hyposaline - 1994,Mesosaline | 0.70 | 3.59 | < 0.05 |
|  | 59 | 2014,Hyposaline - 1999,Mesosaline | 0.74 | 3.76 | < 0.01 |
|  | 60 | 2014,Hyposaline - 2014,Mesosaline | 0.56 | 2.85 | 0.161 |
|  | 61 | 1986,Mesosaline - 1994,Mesosaline | 0.03 | 0.17 | 0.995 |
|  | 62 | 1986,Mesosaline - 1999,Mesosaline | 0.07 | 0.34 | 0.996 |
|  | 63 | 1986,Mesosaline - 2014,Mesosaline | -0.11 | -0.58 | 0.995 |
|  | 64 | 1994,Mesosaline - 1999,Mesosaline | 0.03 | 0.17 | 0.997 |
|  | 65 | 1994,Mesosaline - 2014,Mesosaline | -0.15 | -0.75 | 0.985 |
|  | 66 | 1999,Mesosaline - 2014,Mesosaline | -0.18 | -0.91 | 0.998 |

***Table A3.*** *Spatial increase/decrease in the values of the diversity indices between 1986 and 2014. Values in the third column represents the amount of areas (in percentage) in the Sundarbans where alpha diversity increased, heterogeneity in composition (beta) and subcommunity gamma diversity decreased.*

| Diversity types | | 1986 – 2014 (%) |
| --- | --- | --- |
| Alpha | ${}^{0}\overline{\alpha}$ | 72 |
|  | ${}^{1}\overline{\alpha}$ | 82 |
|  | ${}^{2}\overline{\alpha}$ | 80 |
| Beta | ${}^{0}\overline{\rho}$ | 48 |
|  | ${}^{1}\overline{\rho}$ | 82 |
|  | ${}^{2}\overline{\rho}$ | 86 |
| Gamma | ${}^{0}\gamma$ | 43 |
|  | ${}^{1}\gamma$ | 78 |
|  | ${}^{2}\gamma$ | 98 |

***Table A4.*** *Predictive accuracy (through leave-one-out cross validation) of the kriged diversity models developed for the four time points (1986, 1994, 1999 and 2014) using normalized root-mean-square error (NRMSE) of the predicted versus the actual diversity values. NRMSE is expressed here as a percentage, where lower values indicate less residual variance.*

| Diversity types | | NRMSE (%) | | | |
| --- | --- | --- | --- | --- | --- |
|  |  | 1986 | 1994 | 1999 | 2014 |
| Alpha | ${}^{0}\overline{\alpha}$ | 17.47 | 17.44 | 17.24 | 18.37 |
|  | ${}^{1}\overline{\alpha}$ | 14.16 | 14.00 | 13.86 | 16.03 |
|  | ${}^{2}\overline{\alpha}$ | 14.20 | 13.89 | 14.74 | 16.25 |
| Beta | ${}^{0}\overline{\rho}$ | 14.83 | 14.66 | 27.66 | 24.78 |
|  | ${}^{1}\overline{\rho}$ | 24.72 | 23.39 | 21.32 | 18.90 |
|  | ${}^{2}\overline{\rho}$ | 27.42 | 26.73 | 24.76 | 23.17 |
| Gamma | ${}^{0}\gamma$ | 15.84 | 12.57 | 15.69 | 13.01 |
|  | ${}^{1}\gamma$ | 17.17 | 14.40 | 11.23 | 6.34 |
|  | ${}^{2}\gamma$ | 8.86 | 5.33 | 11.65 | 12.48 |

**
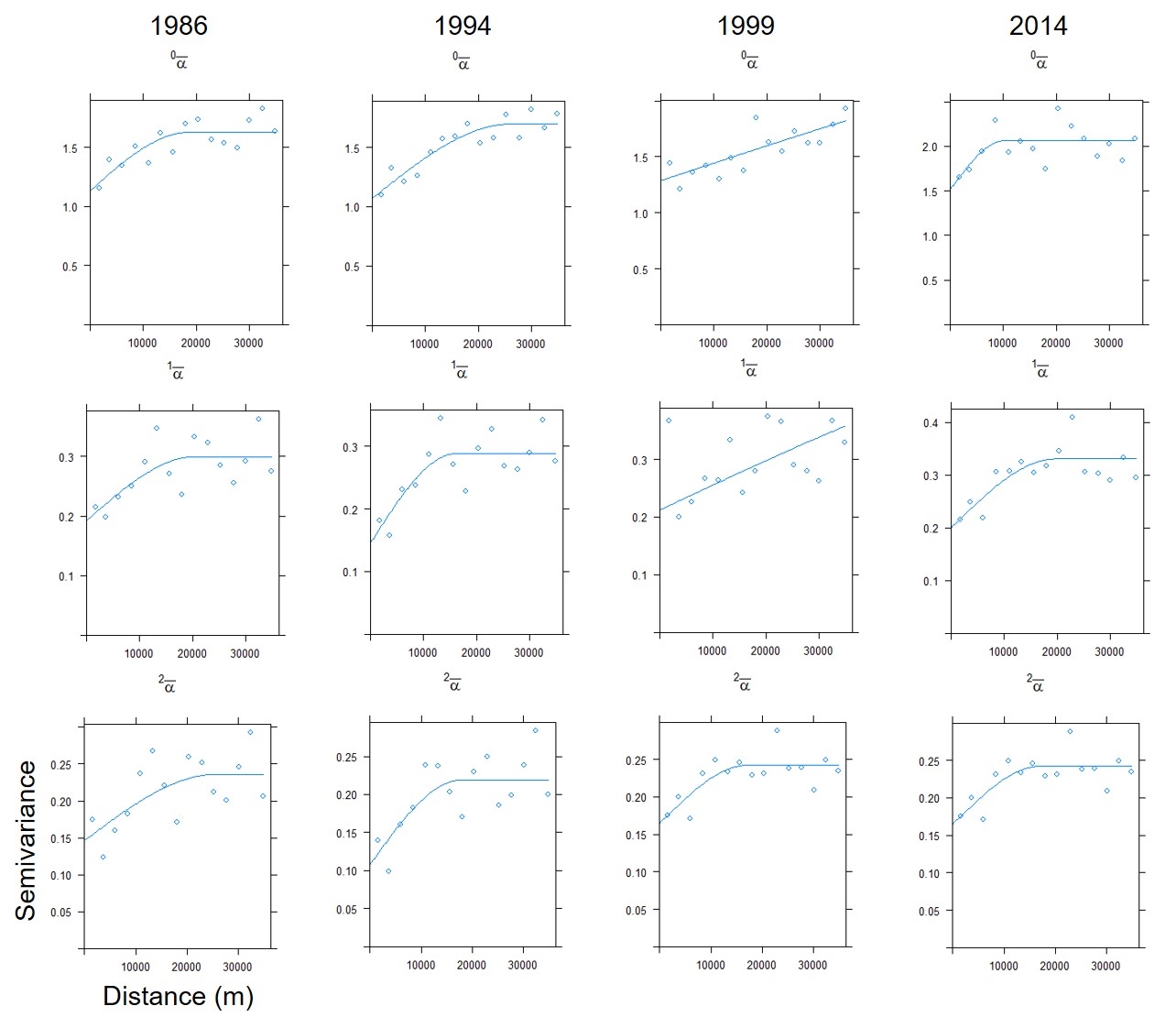
**

***Figure A1.*** *Semivariograms for alpha diversity (q = 0, 1 and 2) in the Sundarbans in four time points since 1986.*

**
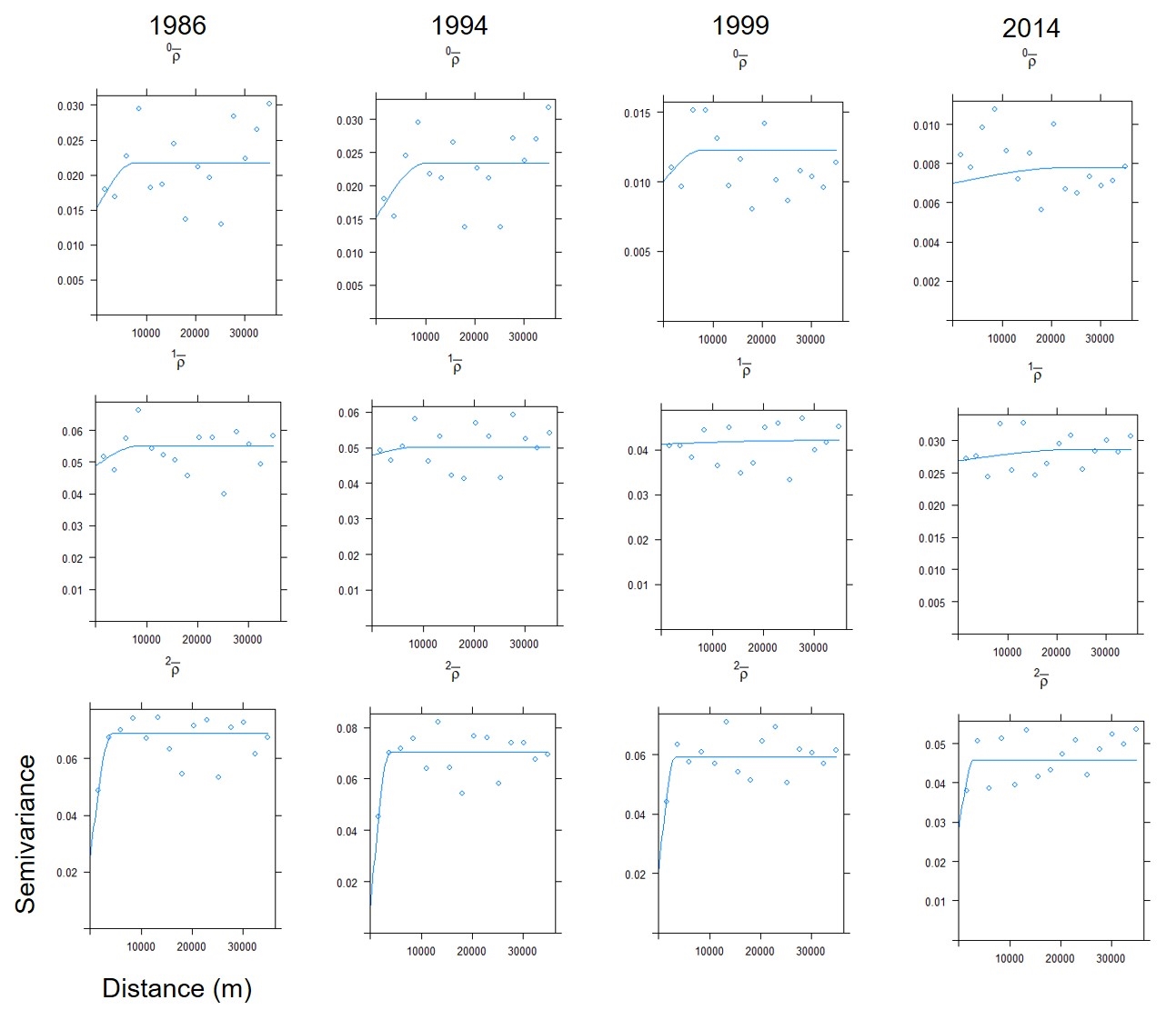
**

***Figure A2.*** *Semivariograms for beta diversity (q = 0, 1 and 2) in the Sundarbans in four time points since 1986.*

**
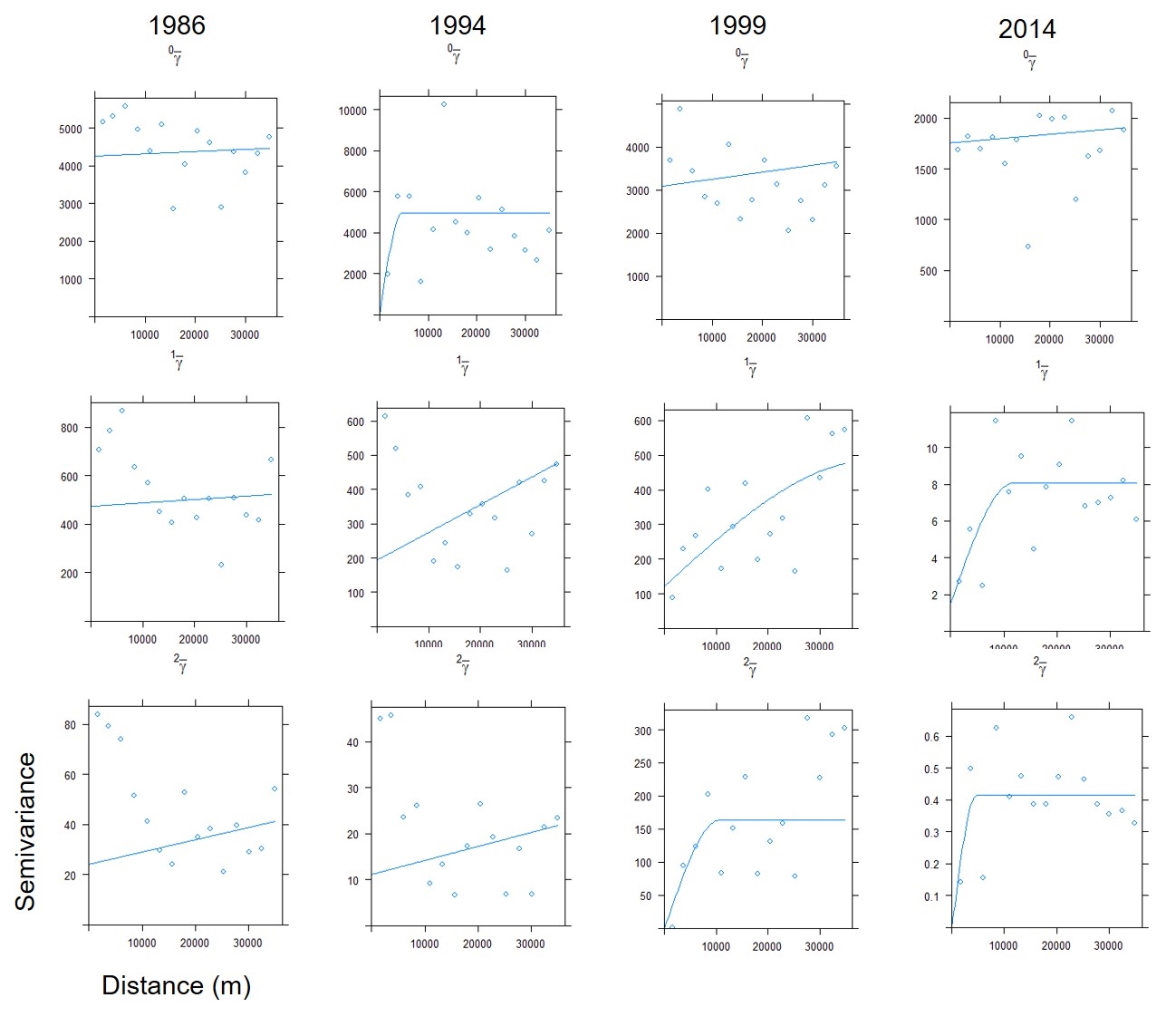
**

***Figure A3.*** *Semivariograms for subcommunity gamma diversity (q = 0, 1 and 2) in the Sundarbans in four time points since 1986.*


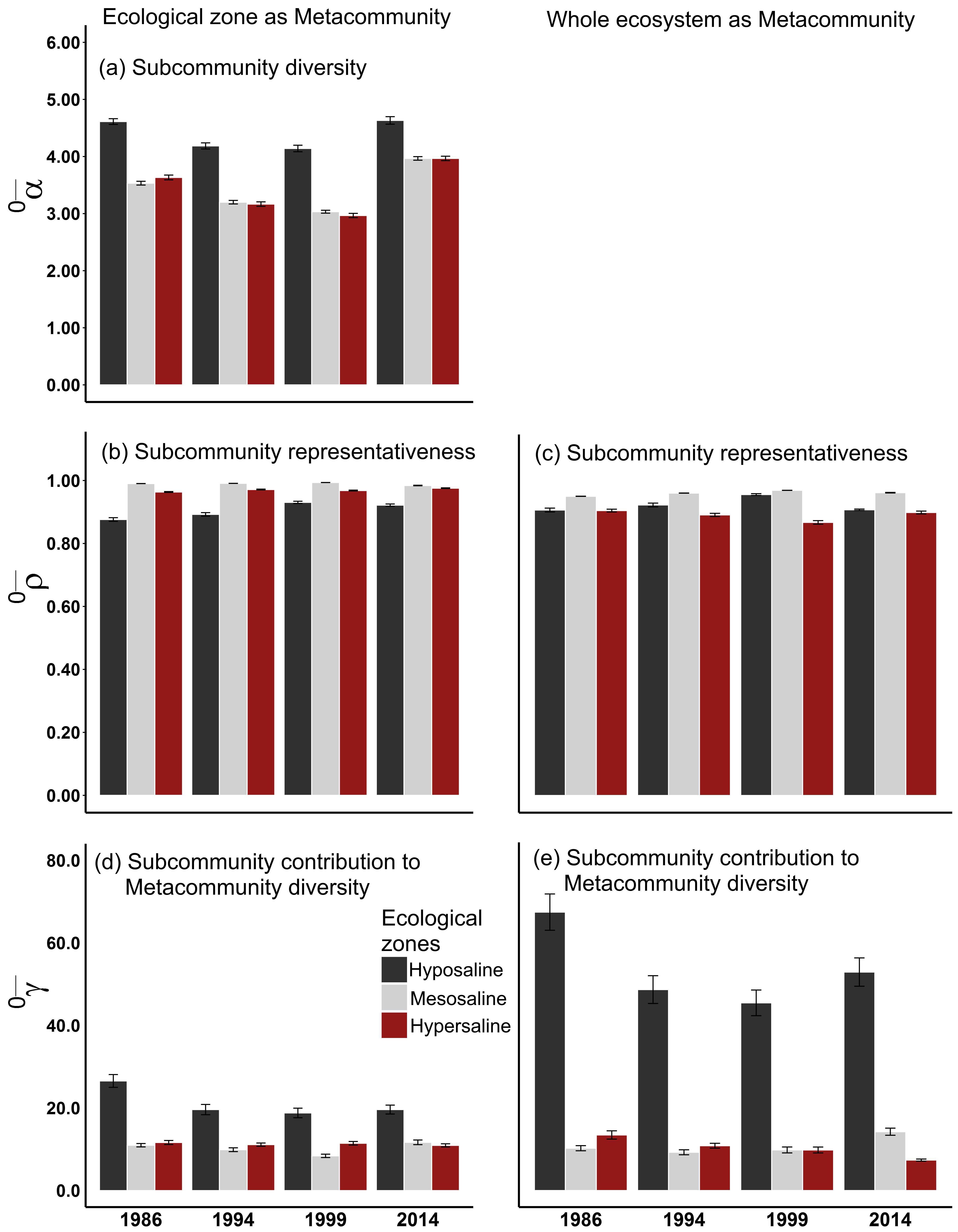


***Figure A4.*** *Bar charts show the spatial (a) alpha (subcommunity diversity), (b & c) beta (subcommunity representativeness), and (d & e) gamma (subcommunity contribution to metacommunity diversity) diversities at q = 0 level for two metacommunity levels – ecological zone and the whole ecosystem – for the four censuses: 1986, 1994, 1999 and 2014. Each permanent sample plot (PSP) is the subcommunity. Each zone as a metacommunity comprises 30 PSPs. The whole ecosystem as a metacommunity comprises 90 PSPs (30 PSPs from each of the three ecological zones). Each bar represents the mean diversity value of the PSPs in each zone or the whole ecosystem and the 95% confidence intervals.*


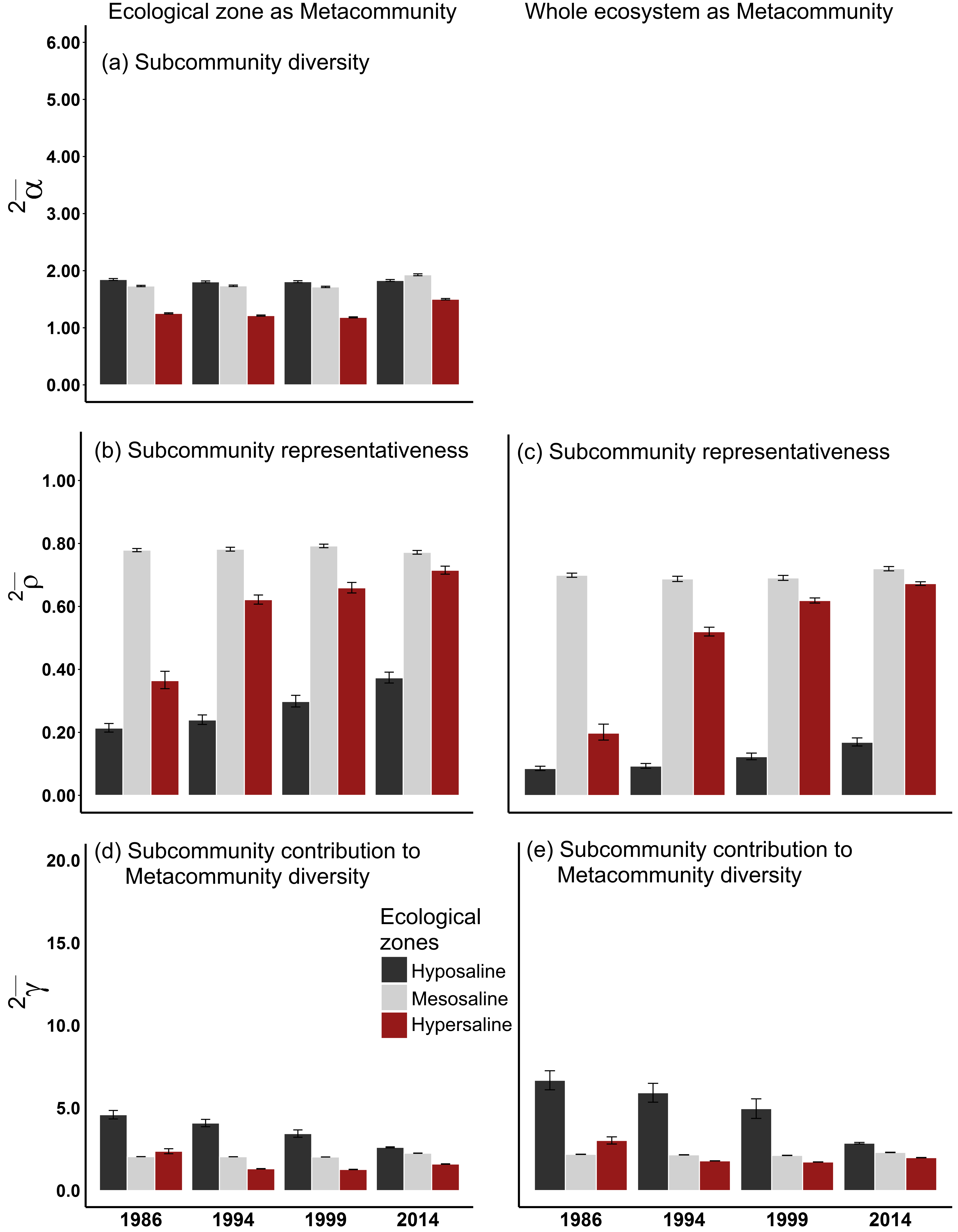


***Figure A5.*** *Bar charts show the spatial (a) alpha (subcommunity diversity), (b & c ) beta (subcommunity representativeness), and (d & e) gamma (subcommunity contribution to metacommunity diversity) diversities at q = 1 level for two metacommunity levels – ecological zone and the whole ecosystem – for the four censuses: 1986, 1994, 1999 and 2014. Each permanent sample plot (PSP) is the subcommunity. Each zone as a metacommunity comprises 30 PSPs. The whole ecosystem as a metacommunity comprises 90 PSPs (30 PSPs from each of the three ecological zones). Each bar represents the mean diversity value of the PSPs in each zone or the whole ecosystem and the 95% confidence intervals.*


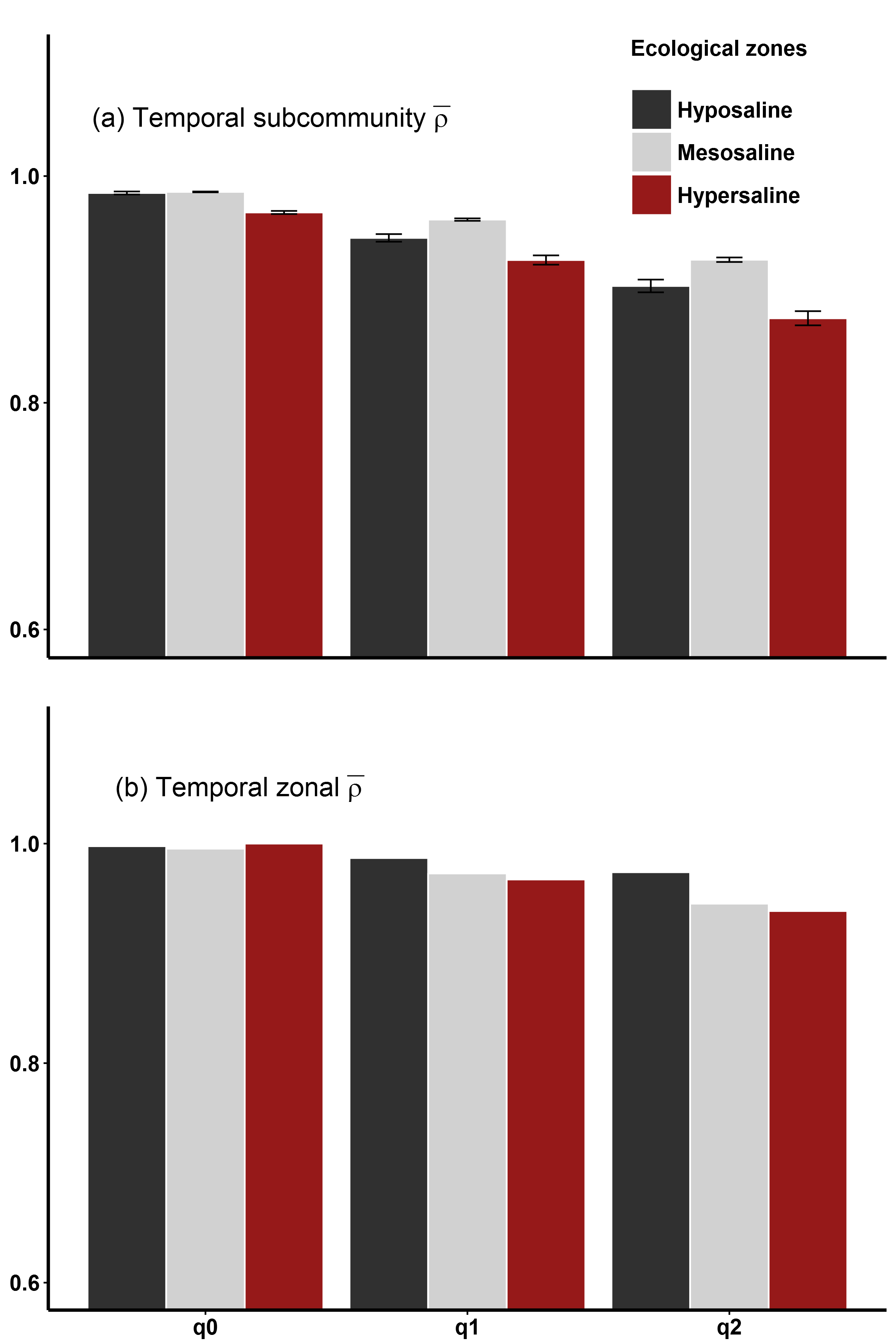


***Figure A6.*** *Bar charts show the temporal dynamics in (a) subcommunity and (b) zonal representativeness (*$\overline{\rho}$*) (for q = 0, 1, and 2) at the ecological zones in the Sundarbans* *over the four census times (1986 – 2014). Here the composition of each permanent sample plot (PSP i.e. subcommunity) or zone summed over 1986 – 2014 form the metacommunity and each PSP/zone composition in each census time is the SC. The calculation of the temporal* $\overline{\rho}$ *follows the same method as for the spatial analysis (see Methods). Each bar in (a) represents the mean representativeness (*$\overline{\rho}$*) of the PSP composition value with the 95% confidence intervals over 1986 – 2014. Here, low temporal representativeness (*$\overline{\rho}$*) and high temporal representativeness (*$\overline{\rho}$*) reflects high and low turnover in species composition, respectively.*





***Figure A7.*** *Spatial distributions of subcommunity alpha, beta and gamma diversities (for q = 0) over the entire Sundarbans generated through ordinary kriging. Higher values of* $\overline{\alpha}$ *and γ indicate greater species diversity and community contribution to the overall diversity of the ecosystem. Lower values of* $\bar{\rho}$ *indicate greater heterogeneity in species composition (i.e. community distinctness from the metacommunity) and higher values of* $\bar{\rho}$ *represent greater representativeness (i.e. homogeneity) in species composition. The black contours represent the three protected areas.*





***Figure A8.*** *Spatial distributions of subcommunity alpha, beta and gamma diversities (for q = 2) over the entire Sundarbans generated through ordinary kriging. Higher values of* $\overline{\alpha}$ *and γ indicate greater species diversity and community contribution to the overall diversity of the ecosystem. Lower values of* $\bar{\rho}$ *indicate greater heterogeneity in species composition (i.e. community distinctness from the metacommunity) and higher values of* $\bar{\rho}$ *represent greater representativeness (i.e. homogeneity) in species composition. The black contours represent the three protected areas.*
